# A Potent Leukocyte Transmigration Blocker: GT-73 Showed a Protective Effect against LPS-Induced ARDS in Mice

**DOI:** 10.3390/molecules26154583

**Published:** 2021-07-29

**Authors:** Eliav Blum, Raanan Margalit, Laura Levy, Tamar Getter, Ron Lahav, Sofia Zilber, Paul Bradfield, Beat A. Imhof, Evgenia Alpert, Arie Gruzman

**Affiliations:** 1 Department of Chemistry, Faculty of Exact Sciences, Campus Ramat-Gan, Bar-Ilan University, Ramat-Gan 5290002, Israel; eliavblum@gmail.com (E.B.); levylaura55@hotmail.com (L.L.); tamargetter25@gmail.com (T.G.); 2 Science in Action, 3 Pinchas Sapir Street, Weizmann Science Park, Ness-Ziona 7403650, Israel; raanan.margalit@gmail.com; 3 AltA-ZuZ Therapeutics, 3 Pinchas Sapir Street, Weizmann Science Park, Ness-Ziona 7403650, Israel; lahav.r@gmail.com; 4 Department of Pathology, Shaare Zedek Medical Center, 12 Shmuel Bait Street, Jerusalem 9103102, Israel; sofiazilber@szmc.org.il; 5 MesenFlow Technologies, Chemin des Aulx, 14, CH-1228 Geneva, Switzerland; paul.bradfield@mesenflow.com; 6 Department of Pathology and Immunology, University of Geneva, Rue Michel-Servet, CH-1211 Geneva, Switzerland; beat.imhof@unige.ch

**Keywords:** ARDS, leukocyte transmigration, cytokine storm, PECAM-1, covalent inhibitor

## Abstract

We recently developed a molecule (GT-73) that blocked leukocyte transendothelial migration from blood to the peripheral tissues, supposedly by affecting the platelet endothelial cell adhesion molecule (PECAM-1) function. GT-73 was tested in an LPS-induced acute respiratory distress syndrome (ARDS) mouse model. The rationale for this is based on the finding that the mortality of COVID-19 patients is partly caused by ARDS induced by a massive migration of leukocytes to the lungs. In addition, the role of *tert*-butyl and methyl ester moieties in the biological effect of GT-73 was investigated. A human leukocyte, transendothelial migration assay was applied to validate the blocking effect of GT-73 derivatives. Finally, a mouse model of LPS-induced ARDS was used to evaluate the histological and biochemical effects of GT-73. The obtained results showed that GT-73 has a unique structure that is responsible for its biological activity; two of its chemical moieties (*tert*-butyl and a methyl ester) are critical for this effect. GT-73 is a prodrug, and its lipophilic tail covalently binds to PECAM-1 via Lys536. GT-73 significantly decreased the number of infiltrating leukocytes in the lungs and reduced the inflammation level. Finally, GT-73 reduced the levels of IL-1β, IL-6, and MCP-1 in bronchoalveolar lavage fluid (BALF). In summary, we concluded that GT-73, a blocker of white blood cell transendothelial migration, has a favorable profile as a drug candidate for the treatment of ARDS in COVID-19 patients.

## 1. Introduction

COVID-19 is a new viral disease affecting many human organs, predominantly targeting the respiratory system and the lungs. All of us are currently trying to survive this deadly global pandemic that seriously challenges and limits public health systems around the world. In about 20% of patients, severe symptoms occur after a mean incubation period of five days. Furthermore, 5% of patients need intensive care therapy, including ventilation support [1,2,3]. 

The main way of obtaining COVID-19 treatment is based on established recommendations for treating patients with acute lung injury (ARDS) and “cytokine storm”. The IL-6 level, in particular, should be reduced dramatically to block the severe results of the “cytokine storm” [4]. Lung ventilation (protective and functional), anti-inflammatory therapy, diuretics, and an adequate management of organ failures constitute the milestones of therapy. In case of significant lung failure, venovenous extracorporeal membrane oxygenation may also be used [5].

ARDS is a major reason for mortality in COVID-19 patients [6,7]. In advanced and especially in the terminal stages of the disease, leukocytes accumulate in the lungs and occupy mainly the alveolar space, including the interstitium due to inflammation induced by viral infection. This first leads to a “cytokine storm”, followed by a devastating destruction of the alveolar tissues [8]. During this stage, the immune system mainly attacks the host tissue; the adaptive immune cells are not yet effective in fighting the virus by antibodies or the specific killing of infected host cells [9]. Thus, blocking this process may successfully treat and reverse the effects of ARDS in COVID-19 patients, similar to the results obtained with our in vivo animal model.

We recently developed a potent inhibitor of leukocyte transmigration from the blood to the tissues (GT-73). This compound was also able to increase the reverse transmigration of monocytes [10]. Compound GT-73, based on the scaffolding of barbituric acid, has already shown highly promising results as a drug candidate for the treatment of other inflammatory and autoimmune disorders (e.g., Crohn’s disease, multiple sclerosis, fatty liver disease, inflammatory bowel disease, and rheumatoid arthritis). This was achieved without any toxic effects on human endothelial cells in vitro. In addition, GT-73 was not toxic in vivo, even when it was used at suprapharmacological doses. 

Despite these promising preliminary results, GT-73’s suggested mechanism of action (blocking PECAM-1 activity) has not been elucidated. We previously reported that according to in silico calculations, GT-73 might affect PECAM-1 activity. PECAM-1 is one of the vascular adhesion molecules that play an important role in leukocyte transendothelial migration during inflammation and cancer [11,12,13]. PECAM-1 not only resides at endothelial junctions, but it is also a constituent of a recycling compartment of endothelial cells, termed the lateral border recycling compartment (LBRC), which most likely provides PECAM-1 molecules to junctions during leukocyte transmigration [14,15]. It was shown that high levels of PECAM-1 lead to increased transendothelial migration of immune cells from blood to organs [16,17,18,19].

Here, we describe the substantial therapeutic effect of GT-73 in LPS-induced ARDS in mice. GT-73 was administered intranasally and had a significant positive effect on the severity of the “cytokine storm” and on lung morphology. We also found that PECAM-1 is indeed a primary target of GT-73 activity. 

Finally, two critical residues for the biological effect of moieties on GT-73 were identified: *tert*-butyl and methyl ester. The absence of these functional groups in the structure of GT-73 completely abolishes its biological activity.

## 2. Results

### 2.1. Structure–Activity Relationships (SARs) and a Possible Mechanism Underlying GT-73 Action

In our previous publication, we reported the screening results (the blocking of leukocyte transendothelial migration) of a series of lipophilic compounds [10]. We carefully studied the structural differences between the nonblocking (GT-77) and the completely blocking (GT-73) compounds [10]. There are two differences between these molecules: (1) the addition of the *tert*-butyl group on the central aromatic ring and (2) the replacement of carboxylic acid with an ester moiety by the addition of a methoxy group. Thus, the importance of both of these chemical moieties in enhancing the biological activity was investigated. 

**Chart 1:**
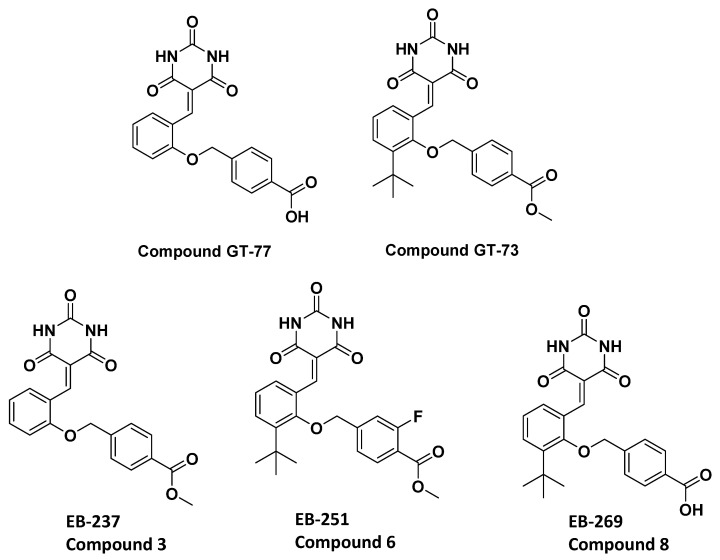
Structures of the synthesized compounds.

**Figure 1:**
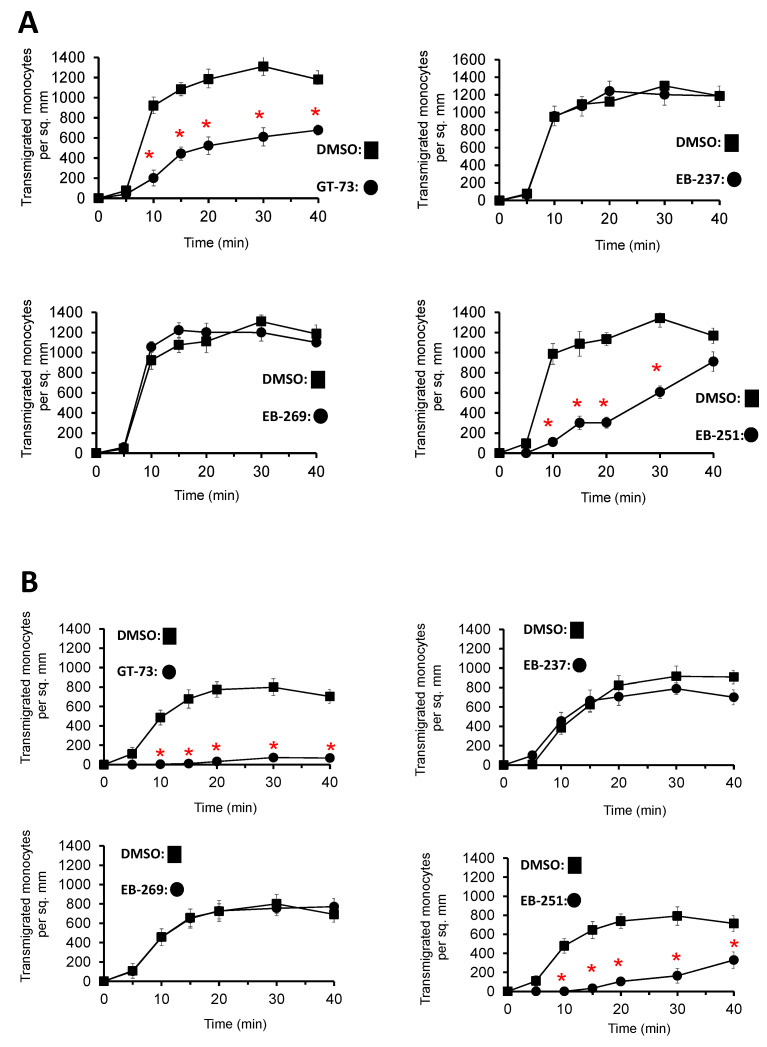
Monocyte transmigration (absolute number) on the surface of the activated HUVECs under flow. Primary human monocytes were cocultured on TNF-α-activated HUVECs. Test compounds at 30 µM or DMSO (0.1%) were added to cells for 45 min. Cocultures were kept under a flow of 0.05 Pa. Mean ± SE, * *p* ≤ 0.05. (**A**) First donor. (**B**) Second donor.

Two novel compounds (EB-237 and EB-269) were synthesized to determine the importance of the two structural moieties of GT-73 (methyl ester and *tert*-butyl) in enhancing the biological effect. The synthesis was initiated from the commercial materials 4-(chloromethyl) benzoic acid and 4-(bromomethyl) benzoic acid, respectively, using Knoevenagel condensation [20] to obtain EB-237 and EB-269, respectively, as described in Supplementary Schemes S1 and S2. Both compounds were tested for their ability to bridge the gap between GT-77 and GT-73 [10]. Therefore, we added to the basic scaffold (GT-77) only one of the supposed functional groups at that time to obtain compounds EB-237 and EB-269. Next, the effect of both compounds GT-73 and its fluorinated version termed EB-251 (Chart 1 and Supplementary Scheme S2) on the transendothelial migration of primary, human monocytes was investigated under flow conditions using inflammatory, TNF-α-activated HUVECs. GT-73 and EB-251 exhibited a significant time-dependent inhibition of monocyte transmigration from two independent donors (Figure 1). In contrast, the novel modified compounds EB-237 and EB-269 did not block this process (Figure 1).

Next, GT-73’s mechanism of action was investigated. According to in silico calculations, the protein affected by GT-73 during monocyte transmigration may be PECAM-1 [10]. Furthermore, we hypothesized that GT-73 could act as a prodrug that binds covalently to PECAM-1. Therefore, the nonstable bond between barbituric acid and the lipophilic moiety might be cleaved by a nucleophilic attack of the primary amine of a lysine located in PECAM-1. Such an attack will result in the formation of a covalent imine bond between the amino acid of the protein and the lipophilic moiety of GT-73 (Supplementary Chart S1). To test this hypothesis, we used mass spectroscopy. To this end, recombinant human PECAM-1 was incubated with GT-73. However, under these conditions, the imine bond would be cleaved, and identifying a covalently attached fragment to PECAM-1 would have been impossible. To avoid this scenario, we added sodium borohydride to the reaction mix before trypsinization, which induces the reduction of the imine bond to a secondary amine, as shown in Supplementary Chart S1. Secondary amines are stable under acidic pH conditions when PECAM-1 is trypsinized as a part of the sample preparation for the mass spec analysis. When inspecting the protein sequence of PECAM-1, we could identify a lipophilic tail of GT-73 as a covalent adduct to Lys^536^, as shown in Supplementary Figure S1.

### 2.2. In Vivo Investigation

**Figure 2:**
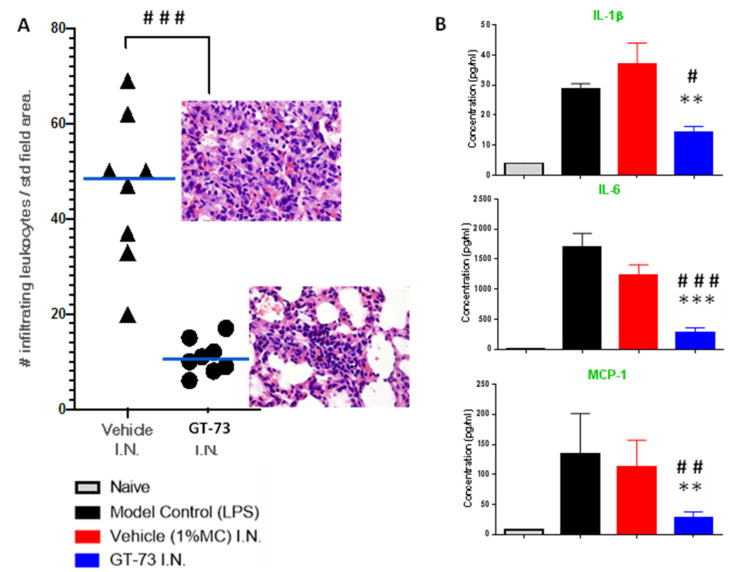
In vivo effect of GT-73. (**A**) Effect of GT-73 on the number of leukocytes in the lungs. Female BALB/c mice were anesthetized and orally intubated with a catheter and challenged with intratracheal instillation LPS (800 μg) in PBS. Mice were euthanized at 3 d after LPS challenge to collect tissues for analysis. All animals from the treatment group were administered GT-73 (5 mg/kg in 1% methyl cellulose) for 3 h after LPS application by the intranasal route 3 times a day. Lungs were collected in 10% buffered formalin, embedded in paraffin, and cut into sections. Sections were stained with hematoxylin/eosin, and images were taken with an Olympus BX53 microscope. (**B**) Samples of BALF were centrifuged. The supernatant was flash-frozen, and the cytokine levels were measured. *n* = 7, MEAN ± SD, significant difference between treated by GT-73 and vehicle groups, ^#^ *p* ≤ 0.004, ^##^ *p* ≤ 0.003, and ^###^ *p* ≤ 0.0001. Significant difference between treated by GT-73 and model control groups ** *p* ≤ 0.003, and *** *p* ≤ 0.0001.

Knowing that GT-73 can covalently bind to PECAM-1 and that it most likely affects its function, we tested GT-73 in a pathology model that leads to severe/terminal stages of COVID-19: ARDS. During the development of ARDS, leukocytes migrate into the lungs, which is accompanied by a “cytokine storm”. We used BALB/c female mice and induced ARDS with intratracheal instillation of LPS. Treatment with GT-73 and vehicle, also administered intratracheally, began 3 h after LPS application. The experiment was then terminated at 72 h. The effect of GT-73 on the number of infiltrating leukocytes in the lungs and the cytokine levels in bronchoalveolar lavage fluid (BALF) were analyzed, as shown in Figure 2A,B, respectively. GT-73 significantly reduced the number of infiltrating leukocytes and significantly reduced the levels of IL-1β, IL-6, and MCP-1 in BALF. 

**Figure 3:**
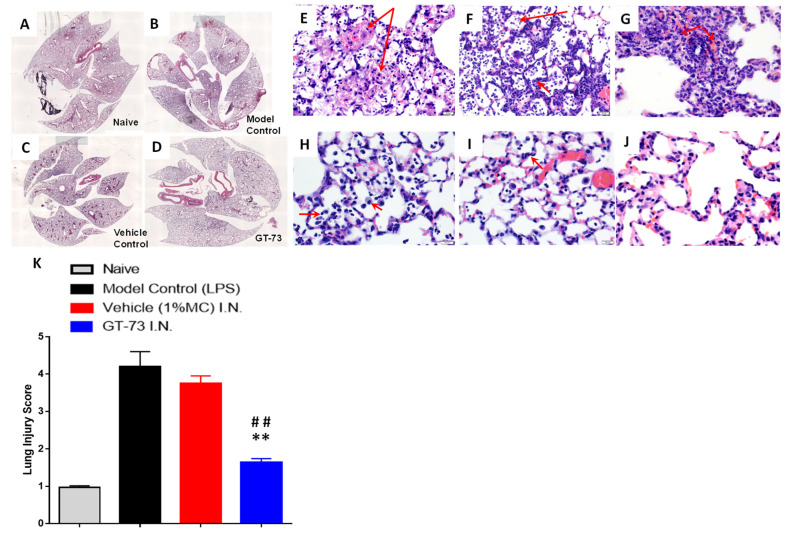
Histological evaluation of the lung inflammation level. Slides were prepared and processed as described in Figure 2. Images of whole-mount lungs were taken at low magnification (×20): (**A**) naive mice; (**B**) untreated model control (LPS); (**C**) vehicle-treated lungs; (**D**) GT-73-treated lungs. Images were taken at high magnification (×400). Upper panel, vehicle-treated lungs: (**E**) fibrin deposits; (**F**) septal and alveolar neutrophils infiltration. (**G**) neutrophilic debris deposits. Lower panel, GT-73-treated lungs: (**H**) alveolar neutrophil infiltration and foaming macrophages; (**I**) septal neutrophil infiltration. (**J**) normal lungs (naïve); (**K**) Lung injury histological score, *n* = 7, mean ± SD, significant difference between treated by GT-73 and the vehicle groups, ^##^ *p* ≤ 0.003. Significant difference between treated by GT-73 and the model control groups ** *p* ≤ 0.003.

To evaluate the potential role of GT-73 on the histopathological changes in the lungs, we produced paraffin-embedded tissue sections and stained them with hematoxylin/eosin. The inflammatory infiltrates, interalveolar septal thickening, and interstitial edema were evaluated (Figure 3A–D). Administration of GT-73 efficiently reduced the airspace inflammation. Furthermore, the severity of lung injury was also assessed using a semiquantitative histopathology score system, which evaluates lung injury according to four categories: alveolar septae, alveolar hemorrhage, intra-alveolar fibrin, and intra-alveolar infiltrates. 

Vehicle and LPS control groups exhibited a prominent neutrophilic infiltrate in the alveolar space and the interstitial septa, but the extent was variable both within and across lobes and in neutrophilic debris deposits, numerous lymphocytes, and macrophages (Figure 3E–K). The ARDS induction affected the air–blood barrier, leading to a progressive breakdown of the capillary walls and the epithelial integrity, which permitted leakage of protein-rich edema fluid (interstitial fluid and plasma proteins) into the alveoli. This proteinaceous fluid presented itself as a lightly eosinophilic material that ranges from homogeneous to fibrillar (i.e., fibrin strands) in appearance in the H&E-stained sections and is present to a variable extent in all specimens. Reactive pneumocytes showed desquamation into alveoli. All specimens collected from the GT-73-treated group showed a diffuse distribution of ill-defined low-density inflammatory lesions, with mild to moderate neutrophilic infiltrate into the alveolar space and interstitial septae, but without neutrophilic debris deposits or lymphocytes; however, there was a higher number of foaming macrophages (indicating the start of the repair phase). In contrast, specimens collected from nontreated mice exhibited huge amounts of neutrophilic debris, high numbers of lymphocytes, and almost no macrophages. In addition, in the treated mice we detected minimal numbers of focal reactive pneumocytes with desquamation into alveoli. Overall, we found that treatment with GT-73 significantly reduced the number of these lung injury scores (Figure 3K).

## 3. Discussion

The presented results substantially enhance our knowledge about GT-73’s mechanism of action and elucidate its function in a COVID-19-relevant inflammatory pathology. It was important to confirm that the chemical structure of GT-73 has two functional moieties: *tert*-butyl and methyl ester, both of which are essential for maintaining the biological activity. Only the replacement of hydrogen by a fluorine atom in the second aromatic ring left the activity intact. The absence of both functional groups in the GT-73 structure led to the complete loss of biological activity. These results might indicate that these two lipophilic groups are required for the binding between GT-73 and the targeted protein. This hypothesis will be investigated in future work. 

We determined, as an adduct to the PECAM-1 sequence, only part of GT-73. Therefore, GT-73 is a classical prodrug and disintegrates after the nucleophilic attack of lys536 in PECAM-1 into two molecules: one is a leaving group (barbituric acid), and the other is the lipophilic alkyl tail, which covalently binds to the lysine by forming an imine bond. Imine bonds can undergo hydrolysis; thus, the covalent inhibition of PECAM-1 is reversible. This may be of importance for therapy and it explains the total absence of toxicity, which was previously reported by our laboratories [10]. The covalent addition of a lipophilic moiety to the extracellular domain of PECAM-1 (lys536 is located extracellularly) may be critical for its function, and it should seriously interrupt the function of PECAM-1, an important adhesion molecule in inflammation-driven leukocyte migration. It might be the basis for the therapeutic effect of GT-73, which we observed in the treatment of ARDS and in other in vivo autoimmune disease models [10].

ARDS contributes to COVID-19-induced patient death. Older age and additional comorbidities increase the rate of mortality from ARDS [21]. ARDS in patients begins with an augmented permeability of alveolar capillaries for proteins such as albumin, fibrinogen, proinflammatory cytokines, and coagulation factors [22]. The next step is the massive transendothelial migration of inflammatory leukocytes, including neutrophils to the lungs. Such a massive accumulation of white blood cells leads to a severe local inflammatory response, a “cytokine storm”, or fibrin deposition; all these factors induce the progression of respiratory dysfunction and heart failure [23]. Recently, suppression of the accumulation of white blood cells in the lungs of COVID-19 patients by dexamethasone (a glucocorticoid that was recently shown to be the first life-saving drug in this disease) was recommended as a mandatory part of the treatment because no other therapies have shown a positive effect during the late or terminal stages of the disease [24]. Here and in our previous publication, we showed that GT-73 inhibits the transmigration of white blood cells [10]. This led to a significantly improved lung morphology after induction of ARDS by LPS. In treated mice, the lungs were cleared from neutrophils and cell debris. GT-73 caused a significant reduction in the number of infiltrated leukocytes but increased the number of macrophages. The latter indicates that the recovery stage in the local inflammatory response had already started. It is known that macrophages play different roles in inflammation, depending on their differentiation stages. In the first (the acute) phase, alveolar macrophages resemble an activated, inflammatory phenotype (M1 in humans); they secrete proinflammatory mediators and factors. In the later phase, the M1 phenotype changes to a rather M2 anti-inflammatory phenotype, and this change allows them to eliminate apoptotic cells, cell debris, and induces the replacement of the dead alveoli by connective tissues [25,26].

Cytokines are deeply involved in the development of inflammation [27,28]. During the onset of an antiviral response, cytokines such as interferons play an important role in the induction of an anti-inflammatory response, but at later stages, other types of cytokines in critical-condition patients can lead to severe tissue damage, as they induce the release of toxic reagents by leukocytes such as cytotoxic reactive oxygen species [29]. Therefore, reducing the extent of the “cytokine storm” is a mandatory step in the treatment of COVID-19 patients. It was recently reported that in patients with severe COVID-19 the levels of IL-2, IL-7, IL-10, GSCF, IP10, MCP-1, MIP1A, and TNF-α are dramatically elevated [30]. In addition, the proinflammatory cytokines IL-6 and IL-1β are also elevated in ARDS patients. This leads to a higher expression of cell adhesion molecules by vascular endothelium and VEGF secretion. The latter augments the vascular permeability of the lung endothelium, allowing quick viral dissemination; the former induces a massive transmigration of neutrophils and inflammatory monocytes from the blood to the lungs. IL-6 and IL-1β also stimulate the proliferation and differentiation of hematopoietic progenitors in bone marrow. As immature granulocytes, they then can go directly to the struggling lungs and further increase local inflammation, which can lead to ARDS. This chain of events in the lung-systemic loop is responsible for the cytokine storm syndrome [31]. GT-73 exhibited significant inhibitory effects on the cytokine level of three cytokines: IL-1β, IL-6, and MCP-1 in the lungs. However, this effect might turn into an important advantage in the fight against the cytokine storm associated with ARDS.

## 4. Materials and Methods

All chemical reagents, solvents, and acids were purchased from Acros Organic (Fair Lawn, NJ, USA), Alfa Aesar (Ward Hill, MA, USA), Bio-Lab Ltd. (Jerusalem, Israel), and Merck (Rehovot, Israel), and all were used as received. LPS from *Salmonella enteritidis* and methyl cellulose were from Merk (Rehovot, Israel). Anhydrous tetrahydrofuran (THF) was obtained by distillation from a boiled blue mix containing sodium (1% *w/v*) and benzophenone (0.2% *w*/*v*). Column chromatography was performed on silica gel 60 (230–400 mesh; Merck, Darmstadt, Germany). Analytical and preparative high-performance liquid chromatography (HPLC) (Young Lin Instruments, Anyang, South Korea) were performed on LUNA C18 preparative (10 µm, 100 × 30 mm) or analytical (5 µm, 250 × 4.6 mm) columns, both from Phenomenex, Inc. (Torrance, CA, USA). HPLC purification was carried out with an increasing linear gradient of CH_3_CN in H_2_O. The purity of the synthesized compounds was confirmed by HPLC analysis and elemental analysis. Analytical thin-layer chromatography (TLC) was carried out on pre-coated silica gel 60 F254 (Merck, Darmstadt, Germany) sheets using UV absorption and iodine physical adsorption for visualization. Mass spectra were recorded on a Finnigan Model 400 instrument using a QToF microspectrometer (Micromass, Milford, MA, USA), using electrospray ionization (ESI) in the positive ion mode. Data were processed using mass L-ynX ver. 4.1 calculation and deconvolution software (Waters Corp., Milford, MA, USA). High-resolution mass spectra (HRMS) were obtained using an LTQ Orbitrap XL (Thermo Scientific, Waltham, MA, USA). Melting points were measured with a Fisher-Johns melting point apparatus (Waltham, MA, USA). The ^1^H NMR and ^13^C NMR were recorded at room temperature on a Bruker AVANCE NMR spectrometer (Vernon Hills, IL, USA) operating at 300, 400, 600, and 700 MHz and were in accordance with the assigned structures. Chemical shift values were reported relative to tetramethylsilane (TMS), which was used as an internal standard. Chemical shifts were expressed in δ (ppm) and coupling constants (*J*) were in hertz. The splitting pattern abbreviations are as follows: s, singlet; d, doublet; t, triplet; q, quartet; quint, quintet; m, unresolved multiplet due to the field strength of the instrument; dd, doublet of doublets. 

### 4.1. In Vivo Study

#### 4.1.1. Mice

Female BALB/c mice, 6–8 weeks old, were purchased from Envigo (Jerusalem, Israel). 

#### 4.1.2. Murine Model of LPS Induced ARDS

The murine model of LPS-induced ARDS was established as reported^19^. Briefly, female BALB/c mice (*n =* 8 per group) were anesthetized and orally intubated with a sterile plastic catheter and challenged with an intratracheal instillation of 800 μg of LPS (*E. coli* 055:B5; Merck, Rehovot, Israel) dissolved in 50 μL of normal PBS. Naïve mice (without LPS instillation) were injected with the same volume of pyrogen-free PBS to serve as controls. Mice were euthanized 3 d after LPS challenge to collect tissues for analysis.

All animals from the treatment group were administered 20 μL of GT-73 at a 10 mg/mL concentration in 1% methyl cellulose by intranasal route 3 times a day 3 h after LPS application. All animals from the vehicle control group were administered 20 μL of 1% methyl cellulose by intranasal route 3 times a day 3 h after LPS application. Finally, animals from the LPS control group were used as an experimental nontreated model control. Naïve animals were used as a baseline control. The drug candidate was administered at a constant volume dosage for 72 h following LPS administration.

#### 4.1.3. Tissue Collection

Samples of Bronchial Alveolar Lavage fluid (BALF) were collected from each mouse from all groups; they were centrifuged for supernatant and cell pellet separation. The supernatant was snap-frozen to determine the cytokine levels, and cells were used for FACS white cell differentiation. Lungs were collected and filled with 10% buffered formalin, embedded in paraffin, and cut into 4 μm thick sections. Sections were stained with hematoxylin/eosin (H&E), and images were taken with an Olympus BX53 microscope (400×). For the lung injury score, images were evaluated by an investigator who was blinded to the identity of the slides. In brief, the extent of the pathological lesions was graded from 0 to 3 (see Table 1). The score for each animal was calculated by dividing the total score by the number of sections observed.

#### 4.1.4. Histological Evaluation of the Lung Inflammation Level

The histological analysis was conducted based on the criteria listed in Table 1 [32]. 

### 4.2. In Vitro Human Monocyte Trafficking Assay

Leukocyte trafficking assays were conducted as described previously. [10] Briefly, human umbilical vein endothelial cells (HUVECs) were isolated by collagenase treatment of umbilical veins and maintained (passages 2–4) in Medium 199 containing 10% FCS, 15 μg/mL endothelial cell growth supplement (Upstate Biotechnology, Lake Placid, NY, USA), 100 μg/mL heparin, and 50 μM hydrocortisone. For the flow assays, HUVECs were cultured after a maximum of 3–5 passages in chamber slides (m-slide VI 0.1 ibiTreat, IBIDI), previously coated with 0.2% gelatin (Merck, Darmstadt, Germany) and 1 mg/mL Collagen G (Biochrom AG, Berlin, Germany) for 2–3 days and then treated for 24 h with acute or chronic inflammation protocols using tumor necrosis factor α (TNF-α) at 1000 U/mL alone or in combination with interferon γ(IFN γ), at 500 U/mL for chronic inflammatory protocols. [10] The compounds to be tested and the leukocytes were added at 30 μM. Monocytes were purified from EDTA blood collected from healthy donors using Ficoll gradients and negative selection kits (Miltenyi Biotec, Bergisch Gladbach, Germany) for those types of cells. The slides containing the cultured HUVEC monolayers were then mounted to a flow system set at 37 °C, and flow was generated over the HUVEC monolayer by perfusing a wash medium or a medium containing the leukocyte suspension using a calibrated pump. The flow rate, expressed as a measurement of shear stress, represented small venules/capillaries (0.05 Pascal (Pa)). The first stage of the assay was a washing procedure, whereby the wash buffer was perfused over the HUVECs for 10 min. Wash buffer was then perfused over the HUVECs for 10 min to remove unbound chemokines, before purified leukocytes were perfused over the HUVECs for 5 min, followed by 45 min with wash buffer. 

Adhesion events were recorded as the total number of cells per unit field (mm^2^). Transmigration events are presented as a percentage of the total leukocytes captured from flow per unit field. All experiments were carried out using triplicate fields and are presented as a mean value with +standard error measurements (+SEM). Statistical analyses assume a parametric distribution and were conducted using Student’s *t*-test. 

### 4.3. Cytokine Level Determination

The levels of IL-1β, IL-6, and MCP-1 were determined using commercially available ELISA analytical kits (Merck, Rehovot, Israel). The assays were conducted according to the protocols supplied by the manufacturer. 

### 4.4. Synthetic Chemistry

The synthesis and the analytical data for the obtained molecules are presented in the Supplementary Materials.

### 4.5. Mass Spectroscopy

Human recombinant PECAM-1(Seale, WA, USA) (25 µg) was dissolved in PBS buffer, (Merck, Rehovot, Israel) (30 µL). GT-73 was added to the solution in 0.5 µL of DMSO to reach a final concentration of 30 µM. The reaction was incubated under gentle agitation for 40 min at 37 °C. Then, NaBH_4_ in PBS (20 µL) was added to obtain a final concentration of 100 mM, and the reaction mix was kept for an additional incubation time (1 h, at 37 °C). The sample was digested by trypsin, analyzed by LC MS/MS on Q-Exactive (Thermo Fisher, Waltham, MA, USA), and identified by Discoverer software against Uniprot unspecific and specific databases. All the identified peptides were filtered with high confidence, top rank, and mass accuracy. High confidence peptides passed the 1% FDR threshold (*FDR = false discovery rate; it is the estimated fraction of false positives in a list of peptides). Semiquantitation was performed by calculating the peak area of each peptide. 

## 5. Conclusions

We recently developed an inhibitor of leukocyte transmigration from the blood to the peripheral tissues (GT-73). Here, we showed that GT-73 has a unique structure, which is responsible for its biological activity, and two of its chemical moieties (*tert*-butyl and a methyl ester) are critical for this effect. In addition, we showed that GT-73 is a prodrug, and its lipophilic tail covalently binds to PECAM-1 via Lys^536^, forming an imine bond. 

In an LPS-induced ARDS mouse model, GT-73 was given intranasally during the induction phase of ARDS (3 h after the intranasal administration of LPS), with encouraging results. Specifically, all specimens collected from treated animals by the compound group showed a diffuse distribution of ill-defined low-density inflammatory lesions, with mild to moderate neutrophilic infiltrate into the alveolar space and interstitial septa, but without neutrophilic debris deposits or lymphocytic infiltrates, as well as a higher number of foaming macrophages (an indication of a repair phase). In contrast, specimens collected from untreated mice exhibited huge amounts of neutrophilic debris and lymphocytic infiltrates and almost no macrophages. In addition, the treated mice exhibited only minimal amounts of reactive pneumocytes with focal desquamation into the alveolar space, compared with untreated mice, where the number of reactive pneumocytes and desquamation was much higher. Finally, GT-73 significantly reduced the number of infiltrating leukocytes and reduced the level of a “cytokine storm”, manifested by lower levels of IL-1β, IL-6, and MCP-1 in bronchial lavage fluid. 

## 6. Patent

The GT-73 structure and its use as a potential therapeutic agent are protected by a patent: Gruzman et al. (2019). “Novel barbituric acid-based leucocyte transmigration inhibitors as drug candidates for treating inflammatory diseases, autoimmune diseases and cancer”. WO2019043706A1.

## Figures and Tables

**Table 1:** Histological score.

Score	Alveolar Septae	Alveolar Hemorrhage	Intra-Alveolar Fibrin	Intra-Alveola Infiltrations per Field
0	All are thin and delicate	No hemorrhage	No intra-alveolar fibrin	Less than 5 intra-alveolar cells
1	Congested alveolar septae in less than 1/3 of the field	Erythrocytes per alveolus in 1 to 5 alveoli	Fibrin strands in less than 1/3 of the field	5 to 10 intra-alveolar cells
2	Congested alveolar septae in 1/3 to 2/3 of the field	At least 5 erythrocytes per alveolus in 5 to 10 alveoli	Fibrin strands in 1/3 to 2/3 of the field	10 to 20 intra-alveolar cells
3	Congested alveolar septae in more than 2/3 of the field	At least 5 erythrocytes per alveolus in more than 10 alveoli	Fibrin strands in more than 2/3 of the field	More than 20 intra-alveolar cells

## Data Availability

All supplemental data can be found at MDPI. In addition, we will share milligram amounts of GT-73 for biological tests. Any other data will also be made available to any investigator upon request.

## Supplementary Materials

The following are available online. The synthetic chemistry part, PECAM-1 covalent modification by GT-73, analytical chemistry data, and the purity level of the synthesized compounds are available online, Scheme S1: Synthetic pathway of ester derivative 3 (EB-237), Scheme S2: Synthetic pathway of fluorinated derivative 6 (EB-251), Scheme S3: Synthetic pathway of carboxylic acid derivative 8 (EB-269), Chart S1: Schematic representation of the formation of an imine bond between GT-73 and Lys^536^ in PECAM-1, Figure S1: Mass spec determination of the covalent addition of a GT-73 fragment to PECAM-1.

## Sample Availability

All supplemental data can be found at MDPI. In addition, we will share milligram amounts of GT-73 for biological tests. Any other data will also be made available to any investigator upon request. All other samples of the synthetized compounds also are available from the authors.
