# Supplementary material for: A Potent Leukocyte Transmigration Blocker: GT-73 Showed a Protective Effect against LPS-Induced ARDS in Mice
Source: Molecules. 2021 Jul 29;26(15):4583. doi: 10.3390/molecules26154583 (PMC8348436; doi:10.3390/molecules26154583)
Supplement: Supplementary file 1 [file molecules-26-04583-s001.zip › molecules-1146847-supplementary.pdf]

Supplementary materials

A Potent Leukocyte Transmigration Blocker: GT-73 Showed a Protective Effect against LPS-Induced ARDS in Mice

Eliav Blum [1], Raanan Margalit [2], Laura Levy [1], Tamar Getter [1], Ron Lahav [3], Sofia Zilber [4], Paul Bradfield [5], Beat A. Imhof [6], Evgenia Alpert [3,*] and Arie Gruzman [1,*]

[1] Department of Chemistry, Faculty of Exact Sciences, Bar-Ilan University, Campus Ramat-Gan, Ramat-Gan, 5290002, Israel; eliavblum@gmail.com (E.B.); levylaura55@hotmail.com (L.L.); tamargetter25@gmail.com (T.G.)

[2] “Science in Action”, 3 Pinchas Sapir Street, Weizmann Science Park, Ness-Ziona, 7403650, Israel; raanan.margalit@gmail.com

[3] “AltA-ZuZ Therapeutics“, 3 Pinchas Sapir Street, Weizmann Science Park, Ness-Ziona, 7403650, Israel; lahav.r@gmail.com

[4] Department of Pathology, Shaare Zedek Medical Center, 12 Shmuel Bait Street, Jerusalem, 9103102, Israel; sofiazilber@szmc.org.il

[5] “MesenFlow Technologies“,Chemin des Aulx, 14 , Geneva, CH-1228, Switzerland; paul.bradfield@mesenflow.com

[6] Department of Pathology and Immunology, University of Geneva, Rue Michel-Servet , Geneva, CH-1211, Switzerland; beat.imhof@unige.ch

***** Correspondence: genia.alp@gmail.com (E.A.); gruzmaa@biu.ac.il (A.G.)

Table of Contents:

Synthetic chemistry part (Supplementary Schemes S1-3)……………………………..………..S3

PECAM-1 covalent modification (Supplementary Chart S1 and Supplementary Figure S1)….S10

Analytical chemistry; NMR spectra images..……………………………..…..…........................S12

Analytical chemistry Mass spectroscopy data…......………………...…..…..............................S52

Analytical chemistry HPLC data (purity)…………..……………..…………….......................S57

References……………………………………………………...……………………….S58

Synthetic Chemistry part

The compounds **1**, **2**, and **3** (**EB-237**) inSupplementary Scheme S1 were synthesized in a procedure similar to that of Getter et al. [10] but in higher yields. 4-(chloromethyl) benzoic acid was esterified with MeOH in the presence of H_2SO_4 under reflux using the Fischer-Speir esterification reaction to obtain methyl 4-(chloromethyl)benzoate (**1**) in 97% yield. The higher yields obtained (97 % vs. 48% of methyl 4-(bromomethyl)benzoate [10]) are probably due to the use of 4-(chloromethyl) benzoic acid instead of 4-(bromomethyl) benzoic acid. In this reaction, a side product was obtained (methyl 4-(methoxymethyl)benzoate) by the SN_2 reaction of MeOH with 4-(bromomethyl) benzoic acid. Since the chloride ion is less reactive as a leaving group, compared with the bromide ion, this side product was not obtained by using 4-(chloromethyl) benzoic acid as starting material. In **step b,** the obtained ester (**1**) was reacted with 2-hydroxybenzaldehyde using K_2CO_3 and KI in acetonitrile at 70 ^{O}C overnight to yield **2** (91%). Again, the yields increased from 60% [10] to 91%, probably because the reaction was stirred overnight instead of for 5 h. The barbituric acid was coupled with the resulting aldehyde, **2**, in EtOH/H_2O (1:1) under reflux overnight using Knoevenagel condensation to obtain **3** in 88 % yield.

Supplementary Scheme S1: Synthetic pathway of ester derivative 3 (EB-237)[a]

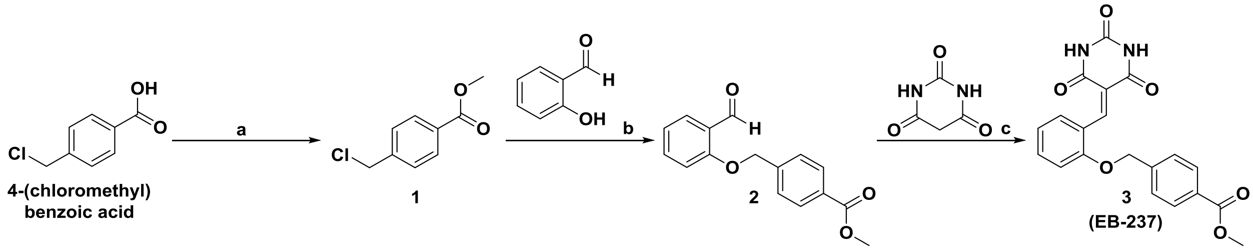

[a]Reagents and conditions: (a) H_2SO_4, MeOH, under reflux for 4 h; (b) K_2CO_3, KI, and CH_3CN, at 70 ^{O}C overnight; (c) EtOH/H_2O 1:1 under reflux overnight.

The next compounds **4**, **5**, and **6** (**EB-251**) [10] were synthesized, starting with 4-(bromomethyl)-2-fluorobenzoic acid, following a similar synthetic pathway (see).

Supplementary Scheme S2: Synthetic pathway of fluorinated derivative 6 (EB-251)[a]

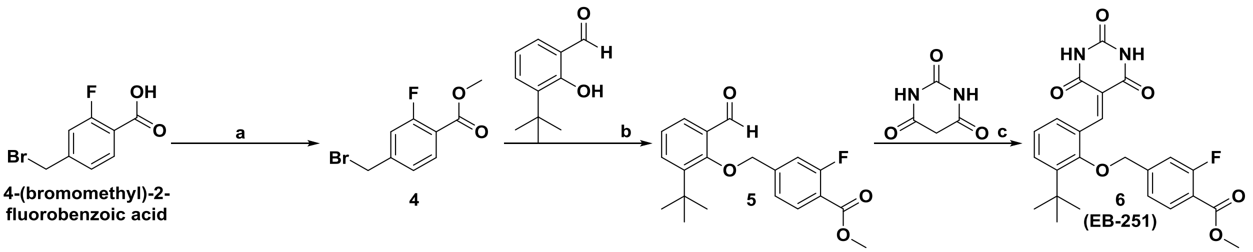

[a]Reagents and conditions: (a) H_2SO_4, MeOH, under reflux overnight; (b) K_2CO_3, KI, and CH_3CN, at 70 ^OC overnight; (c) EtOH/H_2O 1:1 under reflux overnight.

In Supplementary Scheme S3 we started with the synthesis of **GT-73**, as described in Getter et al. [10]. The hydrolysis reaction in **step a**, which was first considered as a simple step, was found to be a challenging one. Instead of the hydrolysis reaction that would yield compound **8** (**EB-269**), a new compound, **7**, was obtained (97% yield) using basic conditions with NaOH in MeOH:1,4 dioxan:H_20 (1:1:1) under reflux due to retro Knoevenagel condensation [33]. Many attempts were carried out to avoid the obtained aldehyde moiety **7**: (1) Using smaller amounts of NaOH (2 eq and even less, instead of 3 eq) for 5 h or less, (2) Using different methods of hydrolysis: (a) Potassium trimethylsilanolate in THF, (b) $AlCl_3$ in Ethanethiol [34], and (c) 4 M HCl in 1,4 dioxane. All these attempts resulted in Product **7**. In light of this, it was decided to react compound 7 again with barbituric acid (1.2 eq) to yield the desired compound 8 (see

, step **b)**. In a first trial, a mixture of the starting material **7** and product **8** were obtained in a 6:4 ratio, respectively. Separation attempts by column flash chromatography (FC) or HPLC failed. It was then decided to react compound **7** with 2 eq of barbituric acid, resulting in product **8**. Compound **8** was not stable during FC or HPLC and could therefore not be purified in this way. Finally, the purified **8** was obtained by trituration in acetone followed by filtration.

Supplementary Scheme S3**: Synthetic pathway of carboxylic acid derivative 8 (EB-269)**[a]

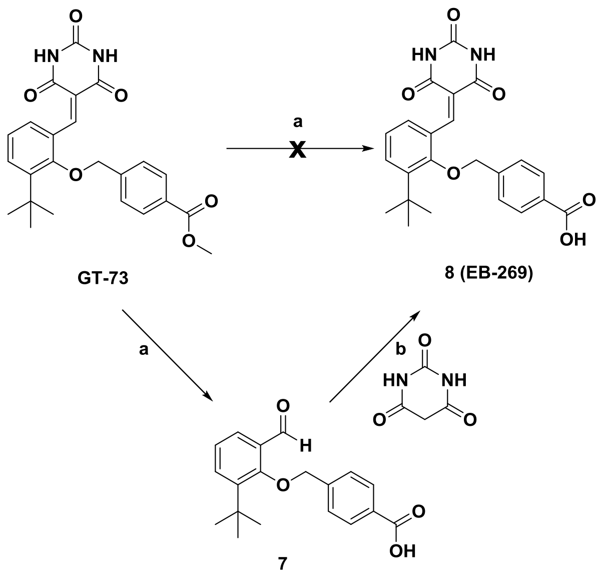

[a]Reagents and conditions: (a) 2M NaOH, MeOH:1,4 dioxan:H_20 1:1:1, under reflux for 5 h; (b) EtOH/H_2O 1:1 under reflux overnight.

Synthetic procedures

Synthesis of methyl 4-(chloromethyl)benzoate (1): Based on the previously published article [10]. Concentrated sulfuric acid (0.71 mL) was added to a solution of 4-(chloromethyl)benzoic acid (2g, 11.72 mmol) in MeOH (30 mL) and refluxed for 4 h. The resulting mixture was cooled to room temperature and concentrated under reduced pressure. H_2O (100 mL) was added to the crude, extracted with EtOAc (3 X 30 mL), washed with brine (30 mL), dried over Na_2SO_4, and concentrated under reduced pressure to yield **1** (2.09 g, 97%), affording a white solid. ^{1}H NMR (300 MHz, $CDCl_3$): δ_H (ppm): 8.03 (d, *J*=8.44 Hz, 2H), 7.46 (d, *J*=8.44 Hz, 2H), 4.62 (s, 2H), 3.92 (s, 3H. ^{13}C NMR (75 MHz, $CDCl_3$) δ, 166.59, 142.22, 130.02, 129.71, 128.49, 52.23, 45.37 ppm. HRMS (ESI): calcd for $C_9H_9ClO_2$ $[M + H]^+$, 185.0364; found, 185.0365.

Synthesis of methyl 4-((2-formylphenoxy)methyl)benzoate (2): Based on the previously published article [10]. A suspension of **1** (0.6 g, 3.2 mmol), 2-hydroxybenzaldehyde (0.35 mL), K_2CO_3 (6.4 mmol), and KI (3.9 mmol) in acetonitrile (10 mL) was heated at 70 ºC overnight. The resulting mixture was cooled to room temperature and concentrated under reduced pressure. The residue was dissolved in EtOAc (25 mL), washed with saturated aqueous $NaHCO_3$ solution (2x10 mL), H_2O (2x10 mL), and brine (2x5 mL), dried over Na_2SO_4, and concentrated under reduced pressure. The crude was purified by flash column chromatography using EtOAc/hexane as an eluent to yield **2** (0.805 g, 91%), as a yellow powder. ^{1}H NMR (400 MHz, $CDCl_3$): δ_H (ppm): 10.57 (s, 1H), 8.08 (d, *J*=8.28 Hz, 2H), 7.86 (dd, *J*=7.65 Hz, *J*=1.69 Hz, 1H), 7.55 (d, *J*=1.69 Hz, 1H), 7.52 (d, *J*=8.28 Hz, 2H), 7.06 (t, *J*=7.65 Hz, 1H), 7.01 (d, *J*=8.30 Hz, 1H), 5.25 (s, 2H), 3.92 (s, 3H). ^{13}C NMR (100 MHz, $CDCl_3$) δ, 189.4, 166.6, 160.7, 141.2, 135.9, 130.0, 128.8, 126.9, 125.3, 121.3, 113.0, 69.9, 52.2 ppm. HRMS (ESI): calcd for $C_{16}H_{14}O_4$ $[M + Na]^+$, 293.0784; found, 293.0778.

Synthesis of methyl 4-((2-((2,4,6-trioxotetrahydropyrimidin-5(2H)-ylidene) methyl) phenoxy)methyl)benzoate (3, EB-237): Based on the previously published article [10]. A suspension of barbituric acid (192 mg, 1.5 mmol) and **2** (473 mg, 1.7 mmol) in EtOH (10 mL), and distilled water (10 mL) was refluxed overnight. The formed solid was collected by sucking filtration and washed with boiling water (3x15 mL), EtOH (3x15 mL), and ether (3x15 mL). The resulting solid was dried under vacuum to yield **3** (**EB-237,** 550 mg, 88%), as a yellow powder. ^{1}H NMR (600 MHz, DMSO-d6): δ_H (ppm): 11.36 (s, 1H), 11.18 (s, 1H), 8.03-7.94 (m, 3H), 7.59 (d, *J*=8.08 Hz, 2H), 7.49 (t, *J*=7.55 Hz, 1H), 7.16 (d, *J*=8.08 Hz, 1H), 7.01 (t, *J*=7.47 Hz, 1H), 5.34

(s, 2H), 3.86 (s, 3H). ^{13}C NMR (150 MHz, DMSO-d6) δ, ppm, 165.8, 163.2, 161.3, 157.6, 150.2, 149.6, 142.1, 133.7, 132.4, 129.3, 129.1, 127.3, 122.0, 119.9, 118.9, 112.3, 69.2, 52.1. HRMS (APCI): calcd for $C_{20}H_{16}N_2O_6$ $[M + H]^+$, 379.0935; found, 379.0927. Anal. Calcd for $C_{20}H_{16}N_2O_6$: C, 63.16; H, 4.24; N, 7.37. Found: C, 62.67; H, 4.23; N, 7.25.

Synthesis of methyl 4-(bromomethyl)-2-fluorobenzoate (4): Based on the previously published article [10]. Concentrated sulfuric acid (0.52 mL) was added to a solution of 4-(bromomethyl)-2-fluorobenzoic acid (2g, 8.58 mmol) in MeOH (22 mL) and refluxed overnight. The resulting mixture was cooled to room temperature and concentrated under reduced pressure. H_2O (100 mL) was added to the crude, extracted with EtOAc (3 X 30 mL), washed with brine (30 mL), dried over Na_2SO_4, and concentrated under reduced pressure. The crude was purified by flash column chromatography using EtOAc/hexane as an eluent to yield **4** (762.6 mg, 36 %), as a colorless oil. ^1H NMR (400 MHz, $CDCl_3$): δ_H (ppm): 7.92 (t, *J*=7.7 Hz, 1H), 7.27-7.14 (m, 3H), 4.44 (s, 2H), 3.93 (s, 3H). ^{13}C NMR (100 MHz, $CDCl_3$) δ, ppm, 164.4 (d, *J*=3.79 Hz), 161.7 (d, *J*=261.3 Hz), 144.7 (d, *J*=8.59 Hz), 132.6, 124.5 (d, *J*=3.36 Hz), 118.4 (d, *J*=10.05 Hz), 117.5 (d, *J*=23.5 Hz), 52.4, 31.0. ^{19}F (376 MHz, $CDCl_3$): δ, ppm, 109.09 (dd, *J*=11.09 Hz, *J*=7.47 Hz) HRMS (ESI): calcd for $C_9H_8BrFO_2$ $[M - H]^-$, 244.9618; found, 244.9612.

Synthesis of methyl 4-((2-(tert-butyl)-6-formylphenoxy)methyl)-2-fluorobenzoate (5): Based on on the previously published article [10]. A suspension of **4** (703 mg, 2.8 mmol), 3-(tert-butyl)-2-hydroxybenzaldehyde, (0.51mL), K_2CO_3 (5.6 mmol), and KI (3.4 mmol) in acetonitrile (10 mL) was heated at 70 ºC overnight. The resulting mixture was cooled to room temperature and concentrated under reduced pressure. The residue was dissolved in EtOAc (25 mL), washed with saturated aqueous $NaHCO_3$ solution (2x10 mL), H_2O (2x10 mL), and brine (2x5 mL), dried over Na_2SO_4, and concentrated under reduced pressure. The crude was purified by flash column chromatography using EtOAc/hexane as an eluent to yield **5** (714.4 mg, 70%) as a white powder, m.p. 85 ºC. ^1H NMR (400 MHz, $CDCl_3$): δ_H (ppm): 10.26 (s, 1H), 8 (t, *J*=7.77 Hz, 3H), 7.74 (dd, *J*=7.63 Hz, *J*=1.75 Hz, 1H), 7.65 (dd, *J*=7.87 Hz, *J*=1.75 Hz, 1H), 7.36 (d, *J*=11.33 Hz, 1H), 7.32 (d, *J*=8.11 Hz, 1H), 7.23 (t, *J*=7.6 Hz, 1H), 5.08 (s, 2H), 3.95 (s, 3H), 1.43 (s, 9H). ^{13}C NMR (100 MHz, $CDCl_3$) δ, ppm, 189.9, 164.6 (d, *J*=3.53 Hz), 162.1 (d, *J*=261.3 Hz), 160.7, 144.0 (d, *J*=8.61 Hz), 143.9, 133.8, 132.5, 129.9, 128.9, 124.5, 121.7 (d, *J*=3.49 Hz), 118.1 (d, *J*=10.17 Hz), 115.0

(d, *J*=23.88 Hz), 78.1, 52.4, 35.3, 30.8. ^{19}F (376 MHz, $CDCl_3$): δ, ppm, 109.02 (dd, *J*=11.35 Hz, *J*=7.38 Hz) HRMS (APCI): calcd for $C_{20}H_{21}FO_4$ $[M + H]^+$, 345.1496; found, 345.1497

Synthesis of methyl 2-(tert-butyl)-6-fluoro-4- ((2-((2,4,6-trioxotetrahydropyrimidin-5 (2H)-ylidene) methyl) phenoxy)methyl)benzoate (6, EB-251): Based on the previously published article [10]. A suspension of barbituric acid (155 mg, 1.21 mmol) and **5** (486 mg, 1.4 mmol) in EtOH (10 mL) and distilled water (10 mL) was refluxed overnight. The resulting mixture was cooled to room temperature and concentrated under reduced pressure. The residue was dissolved in DCM (30 mL), washed with H_2O (20 mL), and brine (20 mL), dried over Na_2SO_4, and concentrated under reduced pressure. The crude was purified by flash column chromatography using EtOAc/hexane as an eluent to yield **6** (**EB-251**, 220 mg, 40%), as a yellow powder, m.p. 220-223 °C. ^1H NMR (400 MHz, DMSO-d6): δ_H (ppm): 11.32 (s, 1H), 11.19 (s, 1H), 8.30 (s, 1H), 7.93 (t, J=7.73, 1H), 7.69 (d, *J*=7.63 Hz, 1H), 7.47 (dd, *J*=7.88 Hz, *J*=1.2 Hz, 1H), 7.45-7.36 (m, 2H), 7.12 (t, *J*=7.83 Hz, 1H), 4.93 (s, 2H), 3.87 (s, 3H), 1.37 (s, 9H). ^{13}C NMR (100 MHz, DMSO-d6) δ, ppm, 163.6 (d, *J*=3.28 Hz), 162.9, 161.1, 160.8 (d, *J*=257.93 Hz), 157.6 (d, *J*=8.61 Hz), 150.2, 150.1, 144.7 (d, *J*=9.29 Hz), 141.6, 131.9, 130.2, 127.7, 122.9, 119.8, 117.26 (d, *J*=10.70 Hz), 115.4 (d, *J*=23.25 Hz), 75.9, 52.3, 34.6, 30.5. ^{19}F (376 MHz, $CDCl_3$): δ, ppm, 110.47 (dd, *J*=11.47 Hz, *J*=7.38 Hz) HRMS (ESI): calcd for $C_{24}H_{23}FN_2O_6$ $[M - H]^-$, 453.1467; found, 453.1476.

Synthesis of 4-((2-(tert-butyl)-6-formylphenoxy)methyl)benzoic acid (7): Based on the previously published article [10]. 2 M NaOH (0.86 mL, 1.72 mmol) was added dropwise to a stirred solution of **GT-73** (0.57 mmol) in 1,4-dioxane/EtOH/water (1:1:1) (4.5 mL). After stirring overnight, the mixture was acidified to pH 3 using 1 M HCl. The organic layer was concentrated under reduced pressure and the residue was dissolved in EtOAc (20 mL), washed with 10% citric acid (2x5 mL) and brine (2x5 mL), dried over Na_2SO_4, and concentrated under reduced pressure to yield **7** (174 mg, 97%), as a yellow powder, m.p. 178-180. ^1H NMR (400 MHz, DMSO-d6): δ_H (ppm): 13.00 (bs, 1H), 10.22 (s, 1H), 8.02 (d, *J*=8.03 Hz, 2H), 7.71 (t, *J*=7.91 Hz, 2H), 7.66 (d, *J*=8.2 Hz, 2H), 7.30 (t, *J*=7.54 Hz, 1H), 5.15 (s, 2H), 1.38 (s, 9H). ^{13}C NMR (100 MHz, DMSO-d6) δ, ppm, 189.9, 166.9, 160.3, 143.2, 141.5, 133.6, 130.2, 129.6, 129.4, 128.3, 126.8, 124.3, 78.5, 34.8, 30.5. HRMS (ESI): calcd for $C_{19}H_{20}O_4$ $[M + Na]^+$, 335.1254; found, 335.1252.

Synthesis of methyl 4-((2-((2,4,6-trioxotetrahydropyrimidin-5(2H)-ylidene) methyl) phenoxy)methyl)benzoate (8, EB-269): Based on the previously published article [10]. A

suspension of **7** (173 mg, 0.55 mmol) and barbituric acid (142 mg, 1.11 mmol) in EtOH (10 mL) and distilled water (10 mL) was refluxed overnight. The organic layer was concentrated under reduced pressure and the residue was dissolved in EtOAc (20 mL), washed with 10% citric acid (2x5 mL) and brine (2x5 mL), dried over Na_2SO_4, and concentrated under reduced pressure. The crude was triturated with acetone (5 mL) at 40 ^oC for 1 h, then filtrated, washed with acetone at 40^oC (3x1 mL) and dried over vacuum to yield **8** (**EB-269**, 154.53 mg, 66%), as a yellow powder, m.p. 276-280. ^1H NMR (700 MHz, DMSO-d6): δ_H (ppm): 12.96 (bs, 1H), 11.32 (s, 1H), 11.17 (s, 1H), 8.36 (s, 1H), 7.97 (d, *J*=8.17 Hz, 2H), 7.70 (dd, *J*=7.79 Hz, *J*=1.19 Hz, 1H), 7.58 (d, *J*=8.17 Hz, 2H), 7.48 (dd, *J*=7.91 Hz, J=1.19 Hz, 1H), 7.12 (t, *J*=7.79 Hz, 1H), 4.92 (s, 2H), 1.38 (s, 9H). ^{13}C NMR (176 MHz, DMSO-d6) δ, ppm, 166.9, 162.9, 161.1, 157.9, 150.3, 150.2, 141.6, 141.5, 130.3, 130.2, 129.3, 127.7, 127.2, 122.7, 119.6, 77.1, 34.6, 30.5. HRMS (ESI): calcd for $C_{23}H_{22}N_2O_6$ $[M - H]^-$, 421.1405; found, 421.1404.

PECAM-1 covalent modification

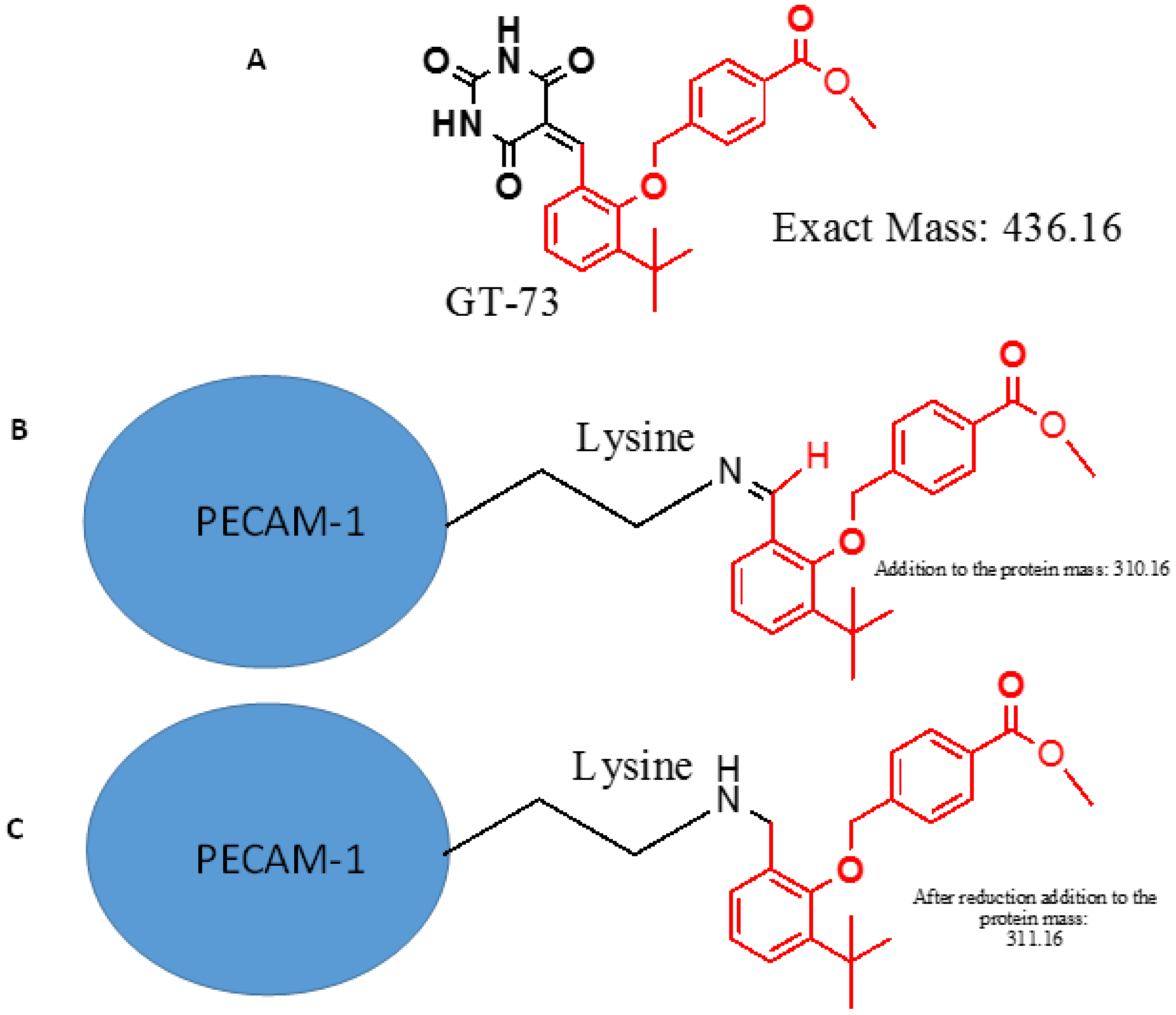

Supplementary Chart S1. Schematic representation of the formation of an imine bond between GT-73 and Lys^{536} in PECAM-1. **A.** GT-73 structure. **B.** Formation of the imine bond. **C.** Formation of the secondary amine after the reduction of the imine bond by sodium borohydride.

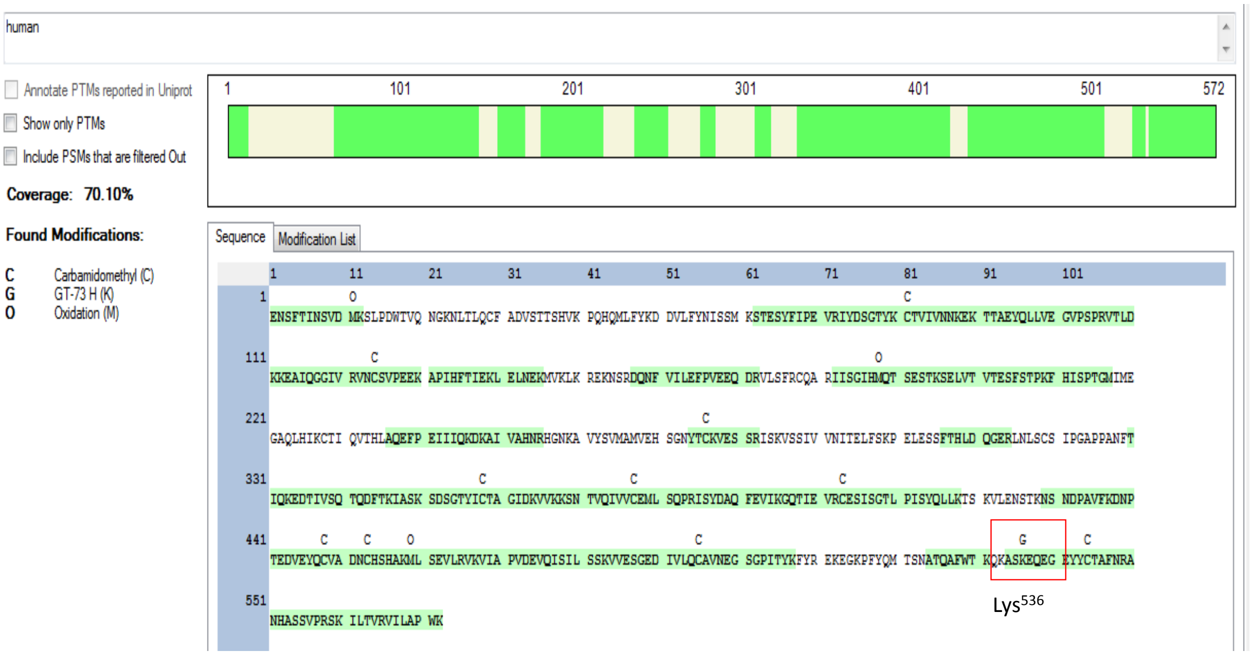

Supplementary Figure S1. Mass spec determination of the covalent addition of a GT-73 fragment to PECAM-1. Human recombinant PECAM1 (25 µg) was mixed with GT-73 (30 µM). The reaction was incubated for 40 min at 37 ºC. Then, $NaBH_4$ in PBS was added to obtain the final concentration as 100 mM and the reaction mix was kept for an additional incubation time (1h, at 37 ºC). The sample was digested by trypsin, analyzed by LC MS/MS; Discoverer software was used. Three covalent modifications were identified: the carbamidomethylation of cysteines, the oxidation of methionines, and the alkylation of lysines by GT-73.

Analytical chemistry

1. NMR spectra images

^{1}H-NMR (300 MHz, $CDCl_3$) - **1**

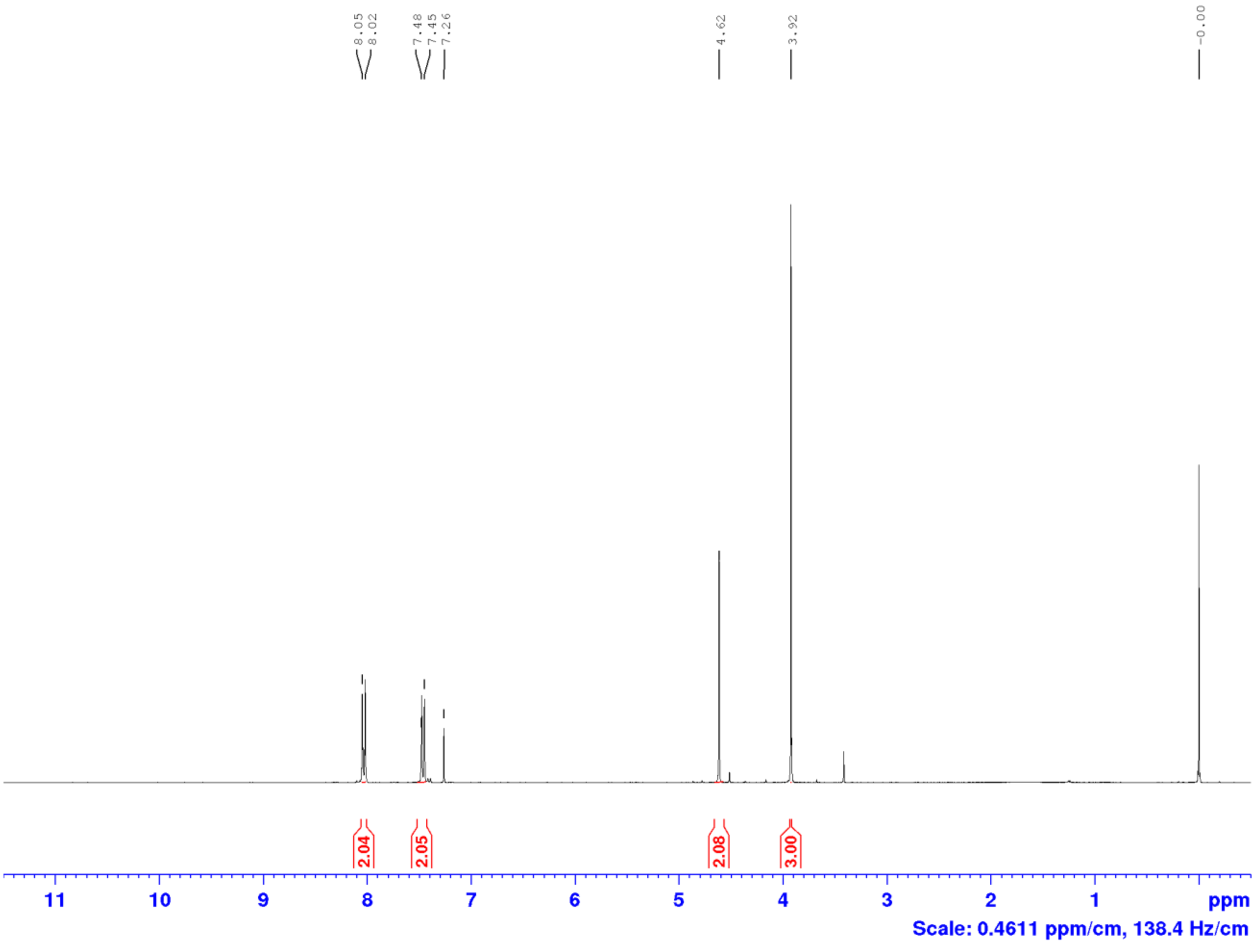

^{13}C-NMR (75 MHz, $CDCl_3$) – **1**

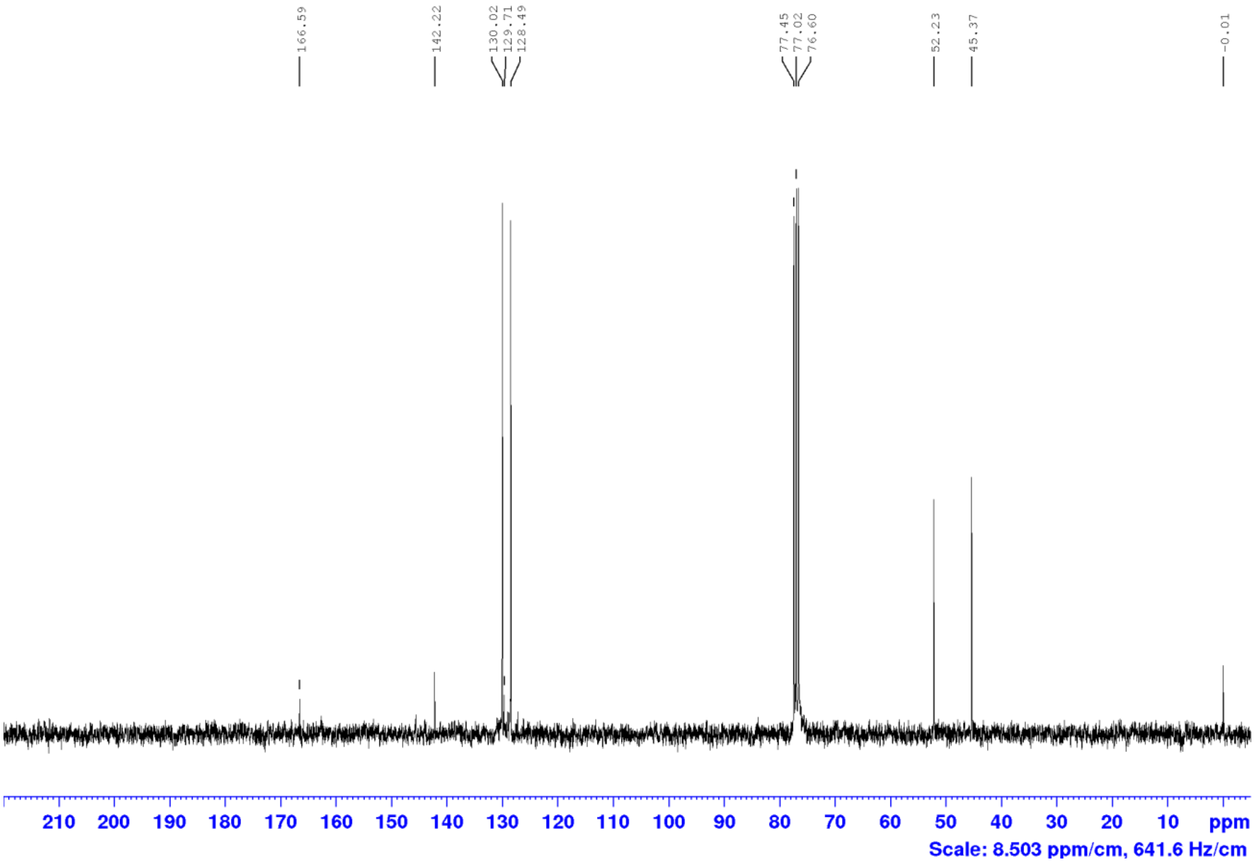

DEPT-NMR (75 MHz, $CDCl_3$) – **1**

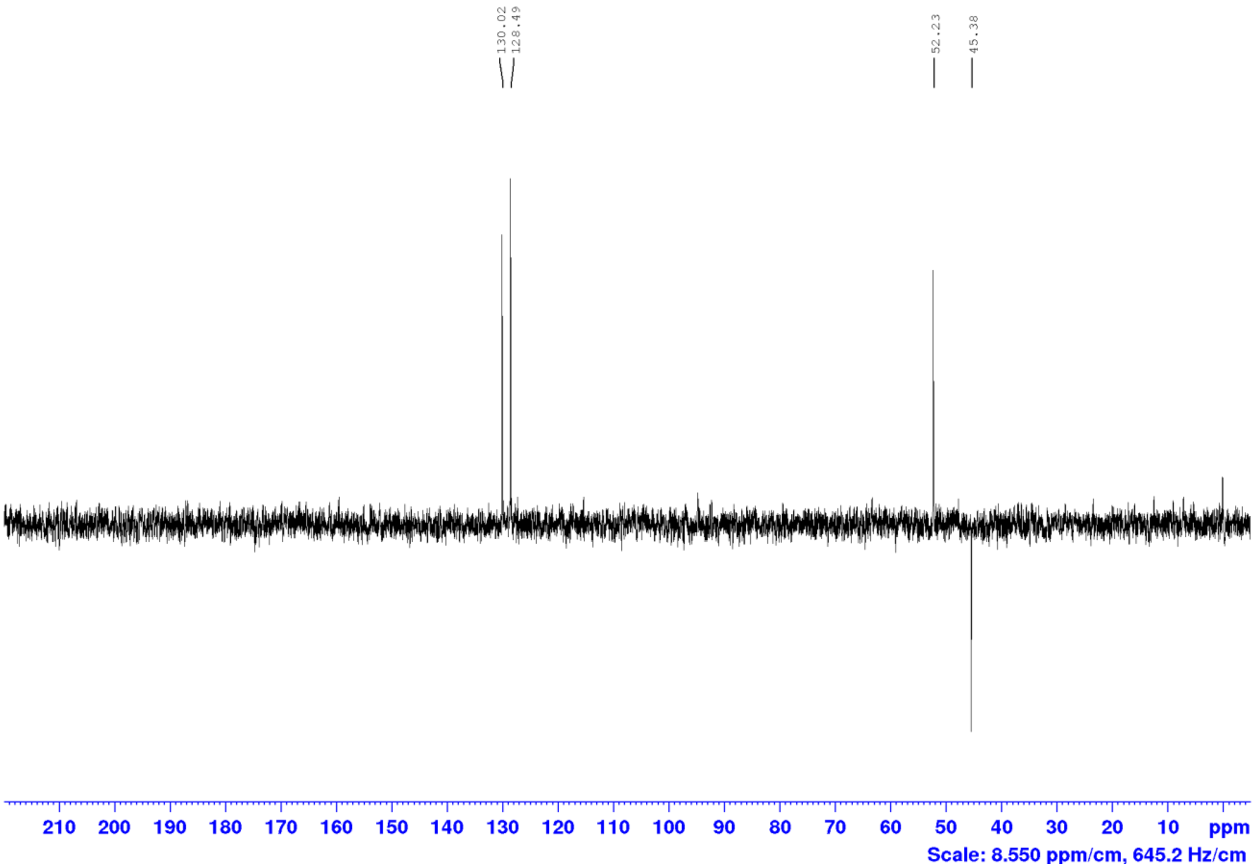

^{1}H-NMR (400 MHz, $CDCl_3$) – **2**

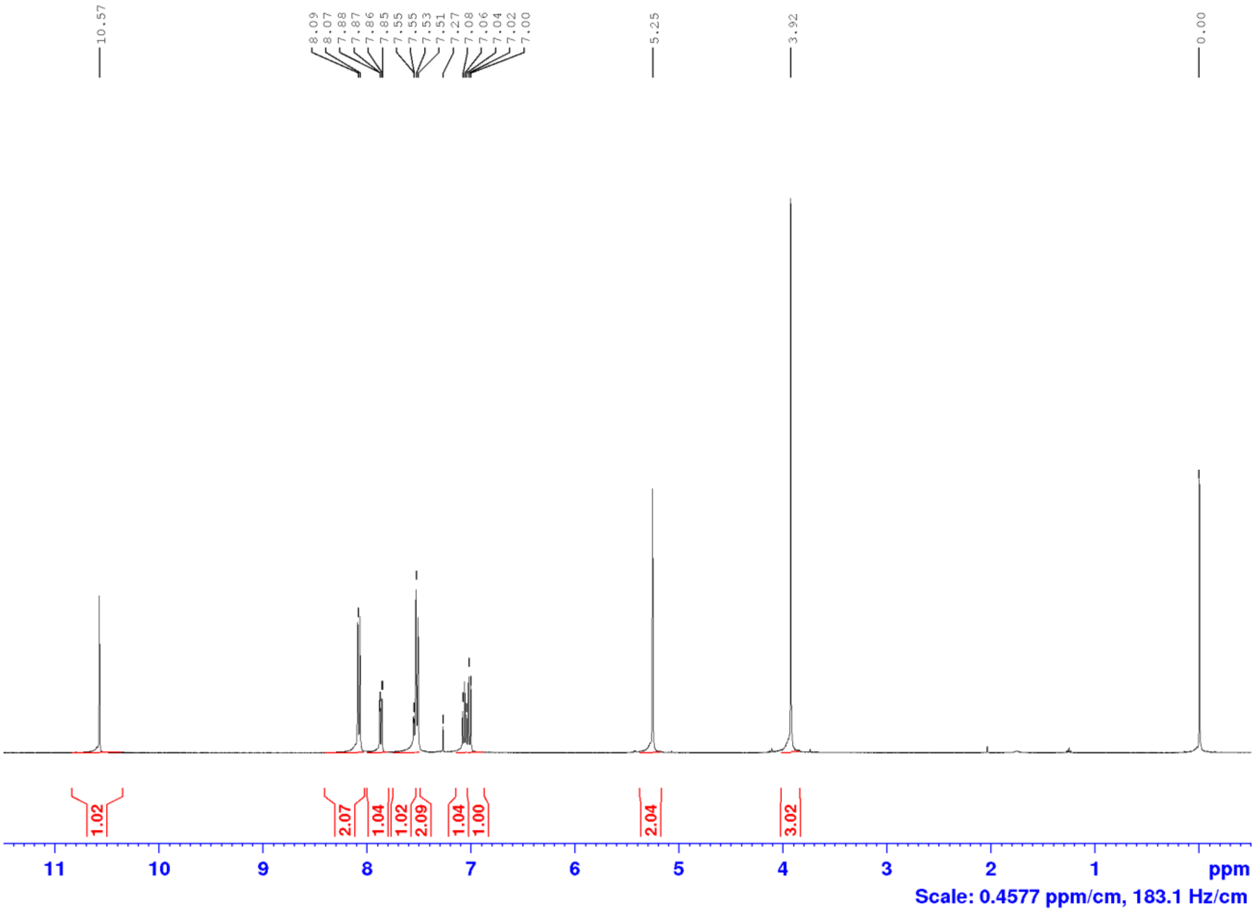

^{13}C-NMR (100 MHz, $CDCl_3$) - **2**

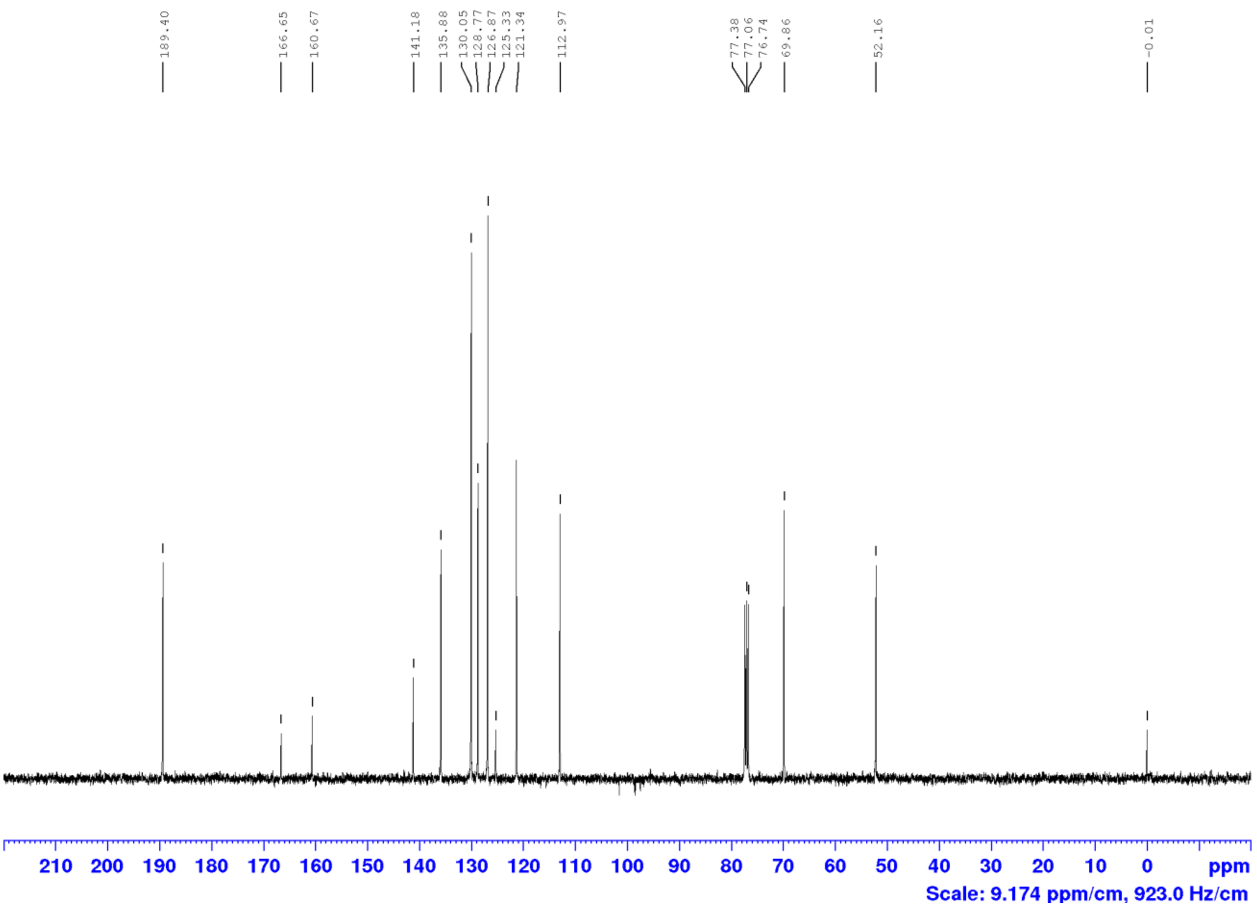

DEPT-NMR (100 MHz, $CDCl_3$) - **2**

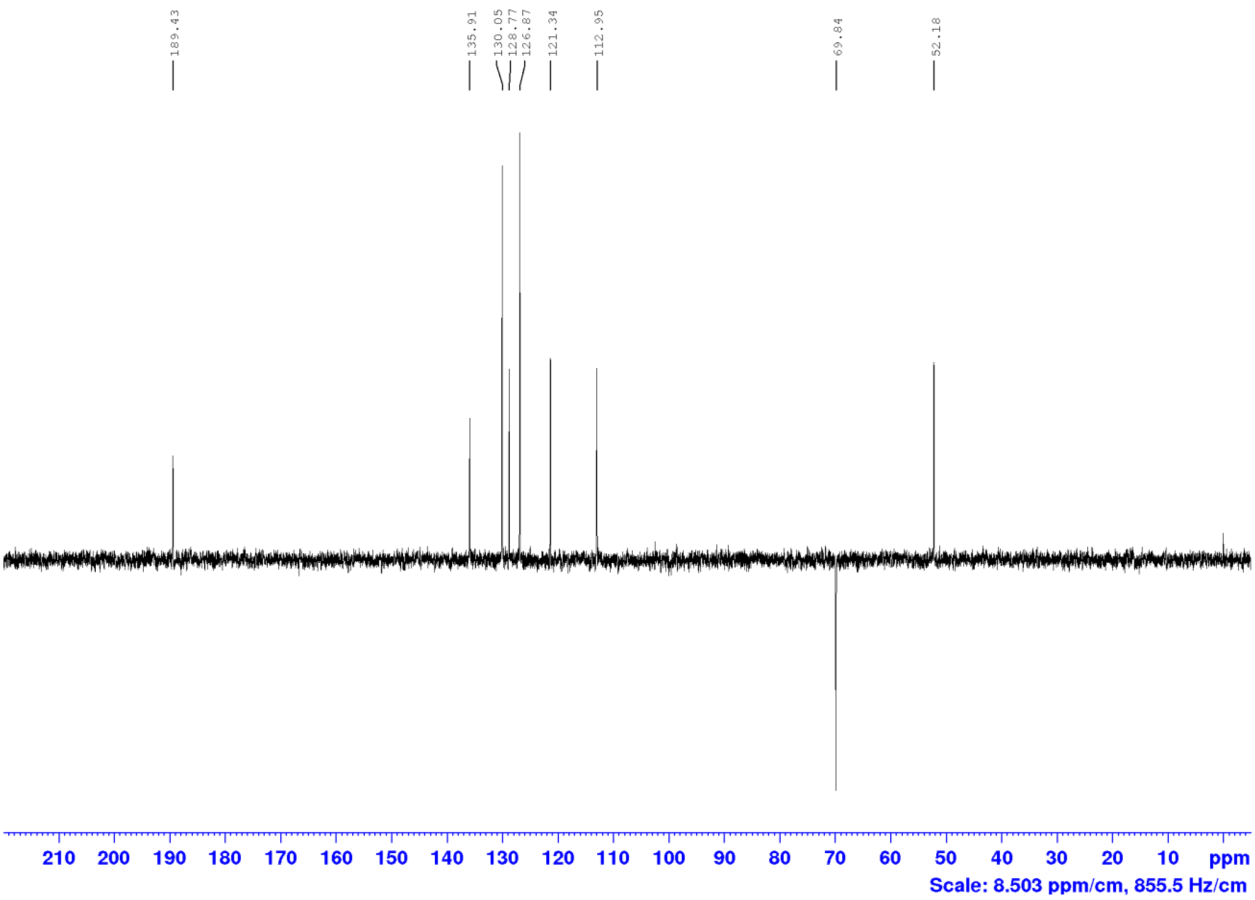

^{1}H-NMR (600 MHz, DMSO-d6) – **3 (EB-237)**

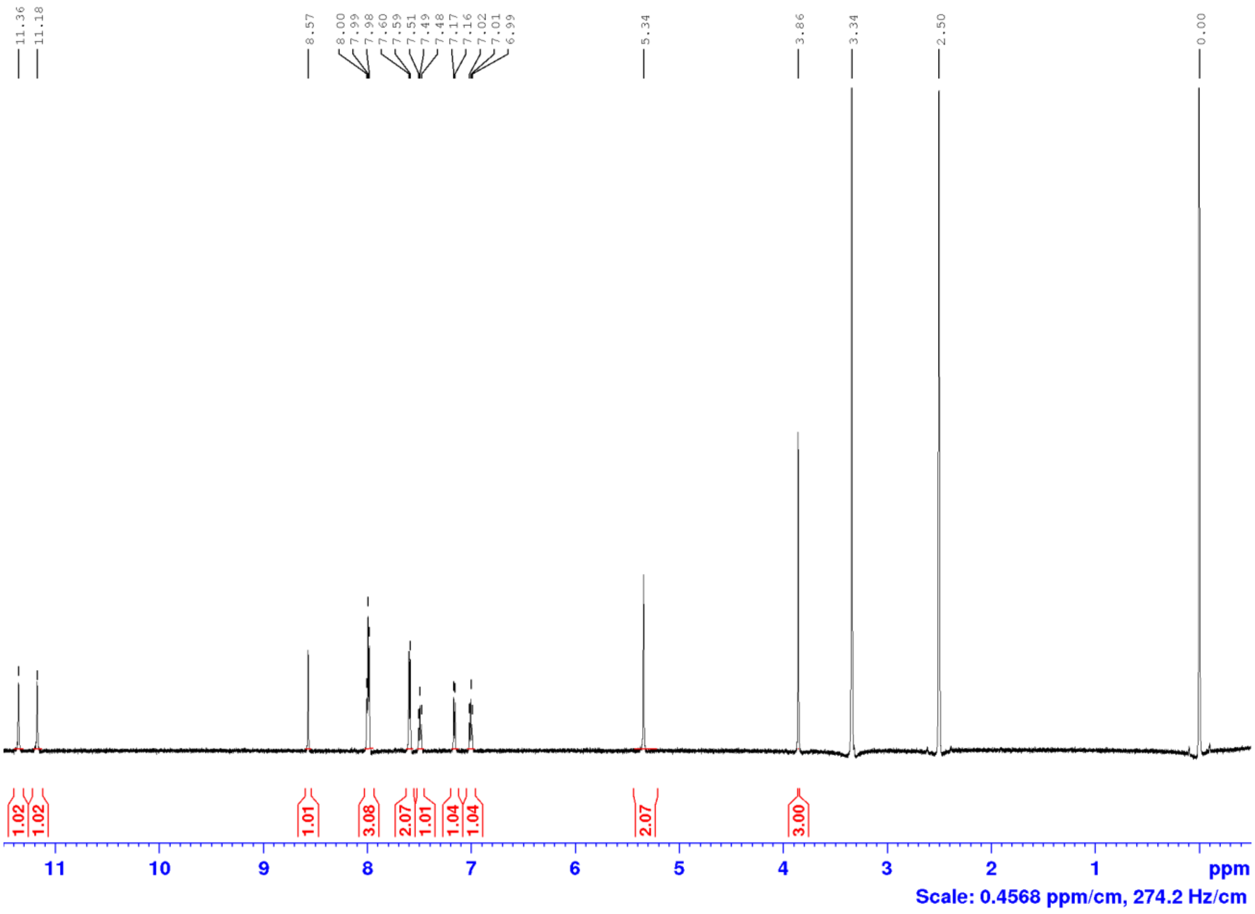

^{13}C-NMR (150 MHz, DMSO-d6) – **3 (EB-237)**

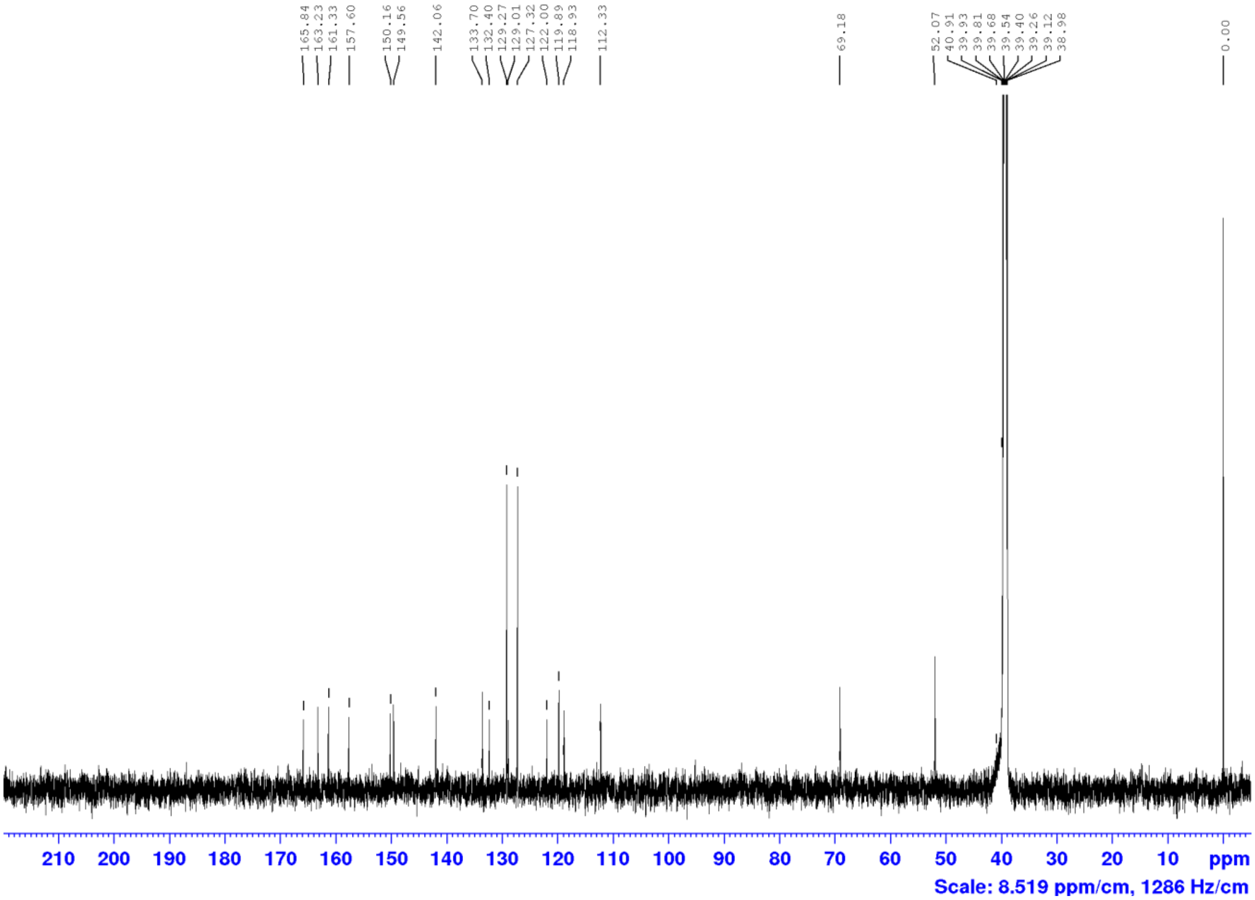

DEPT (150 MHz, DMSO-d6) – **3 (EB-237)**

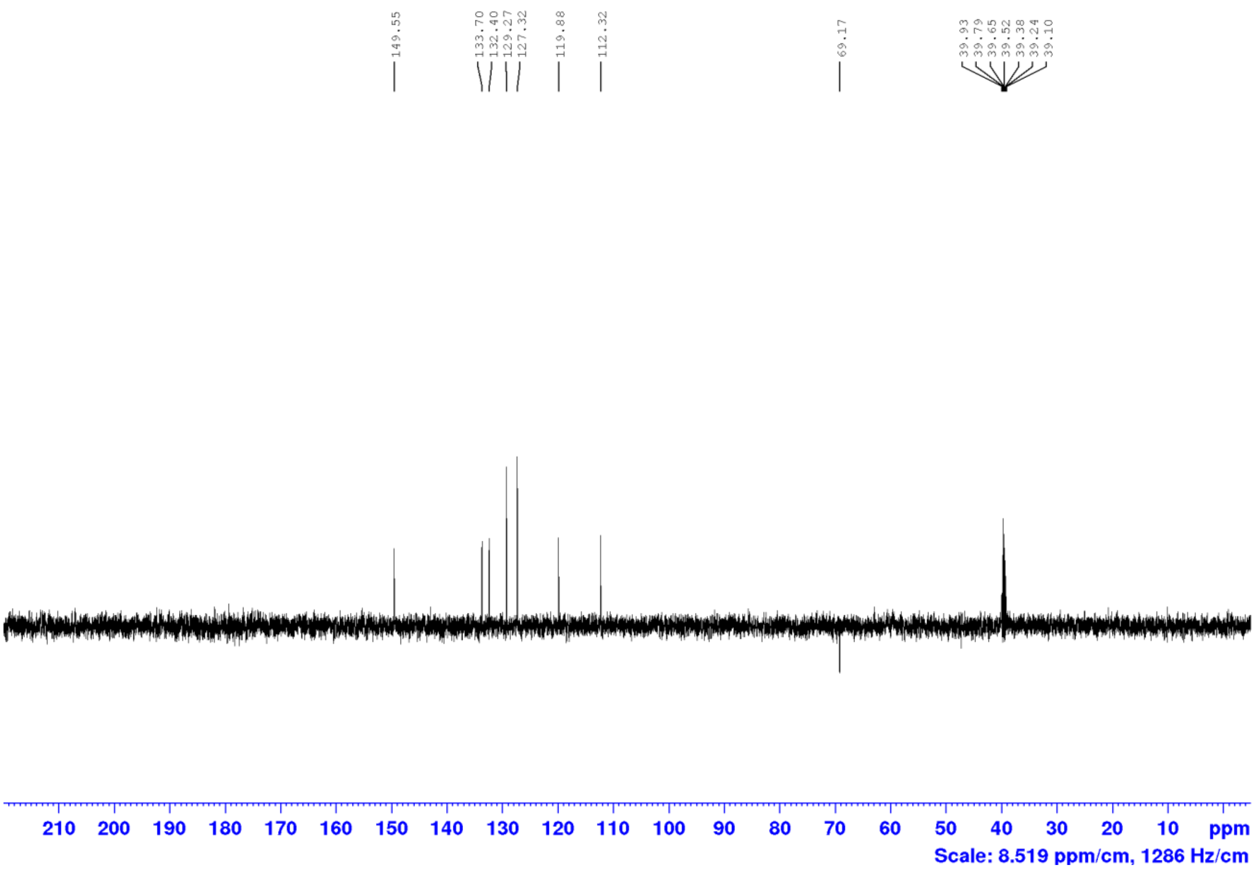

^{1}H-NMR (400 MHz, $CDCl_3$) – **4**

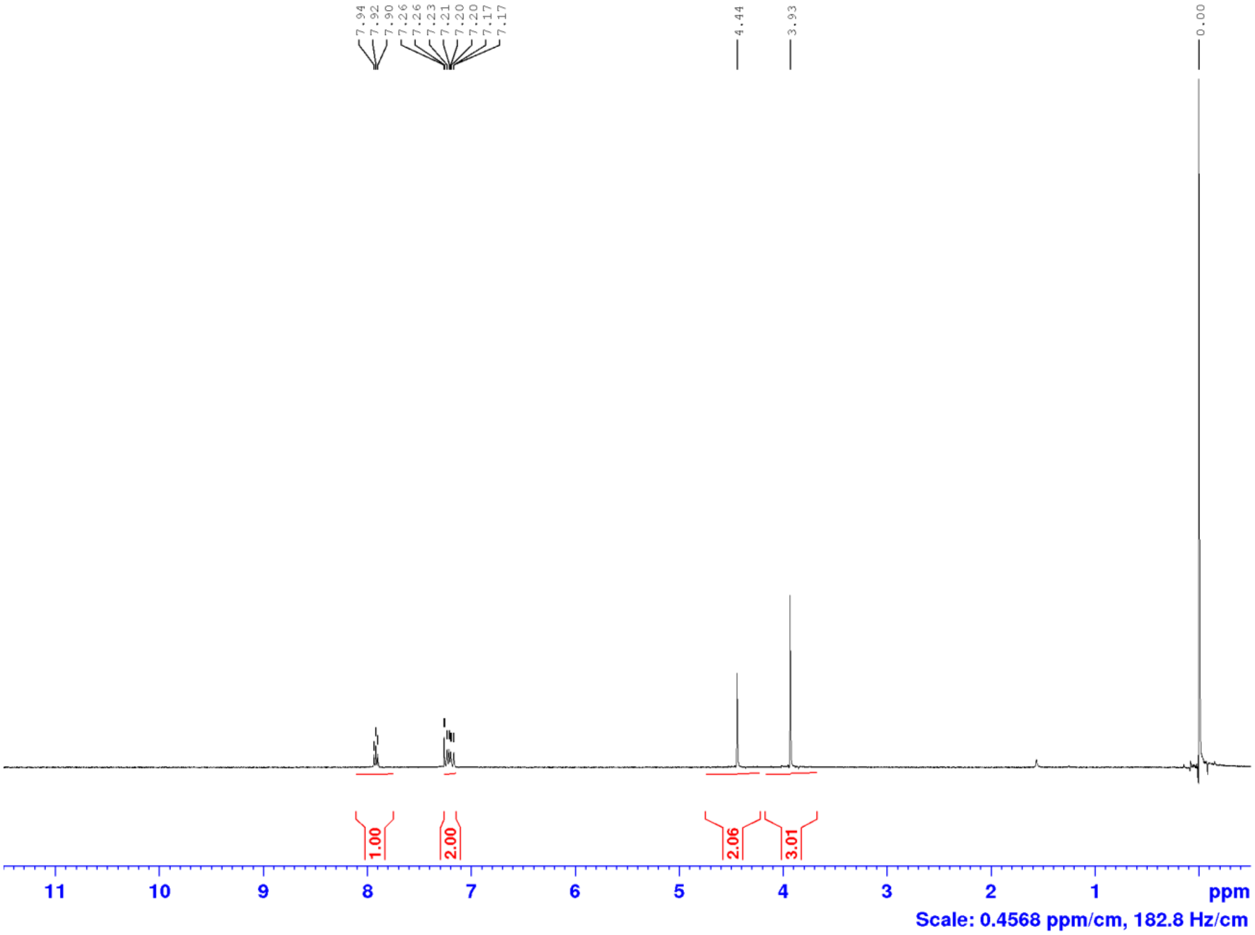

^{13}C-NMR (100 MHz, $CDCl_3$) – **4**

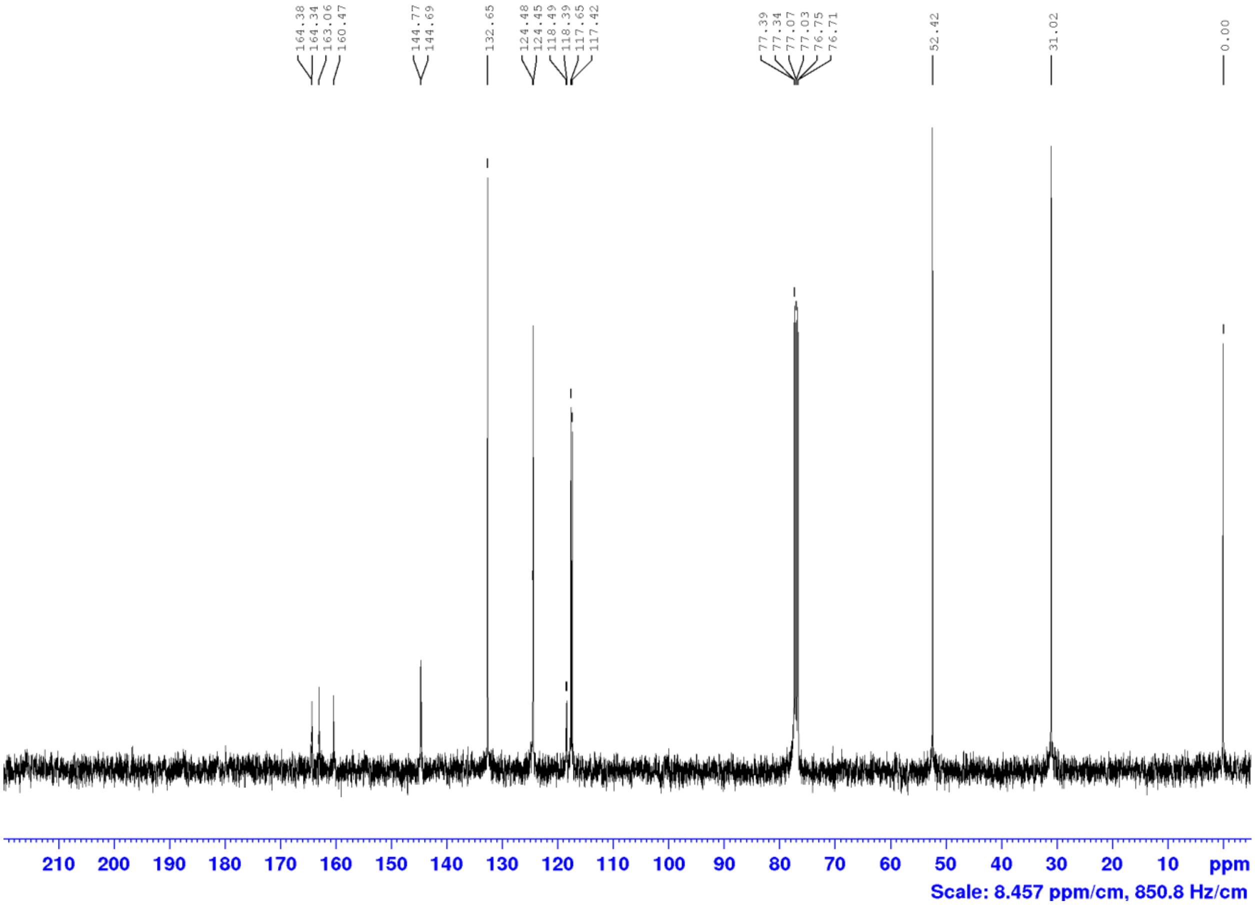

DEPT (100 MHz, $CDCl_3$) - **4**

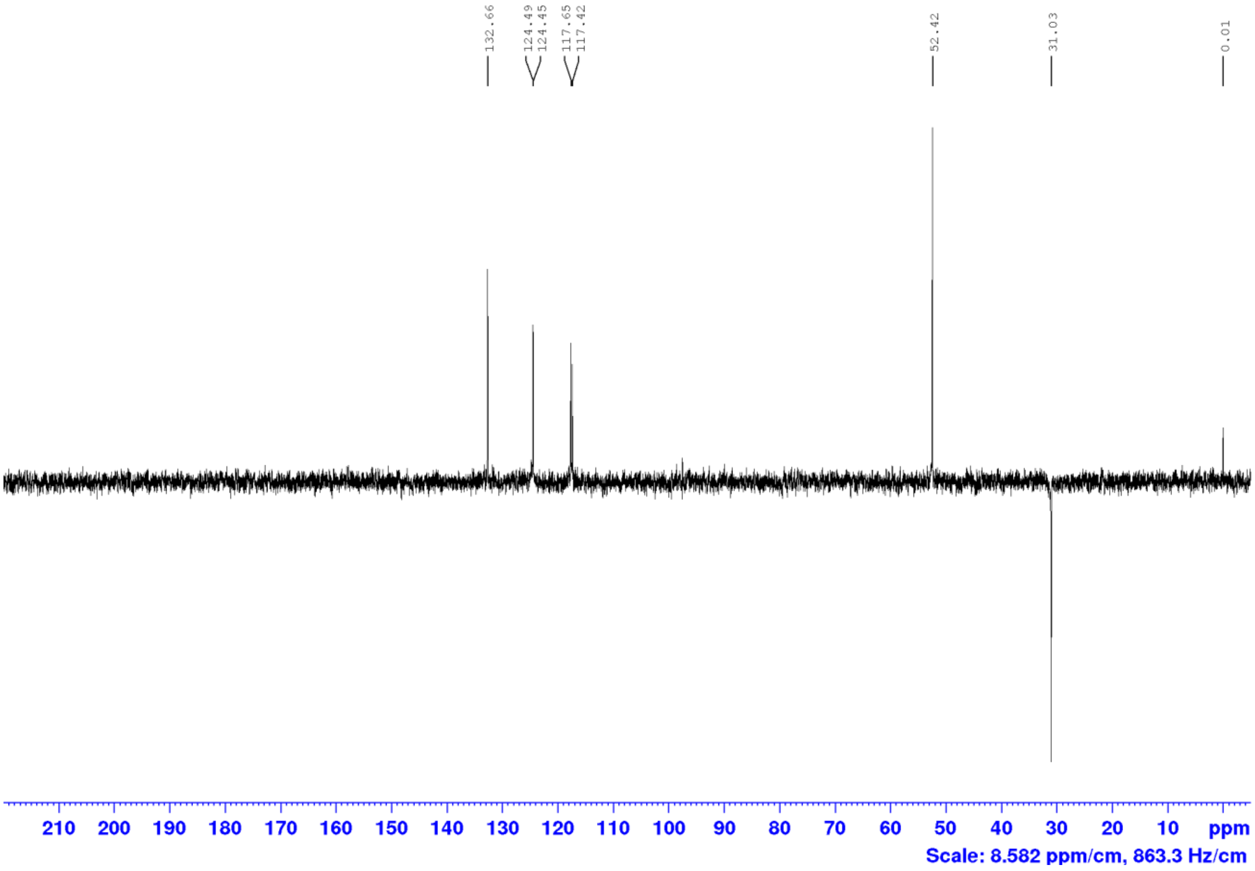

^{19}F-NMR (376 MHz, CDCl3) – **4**

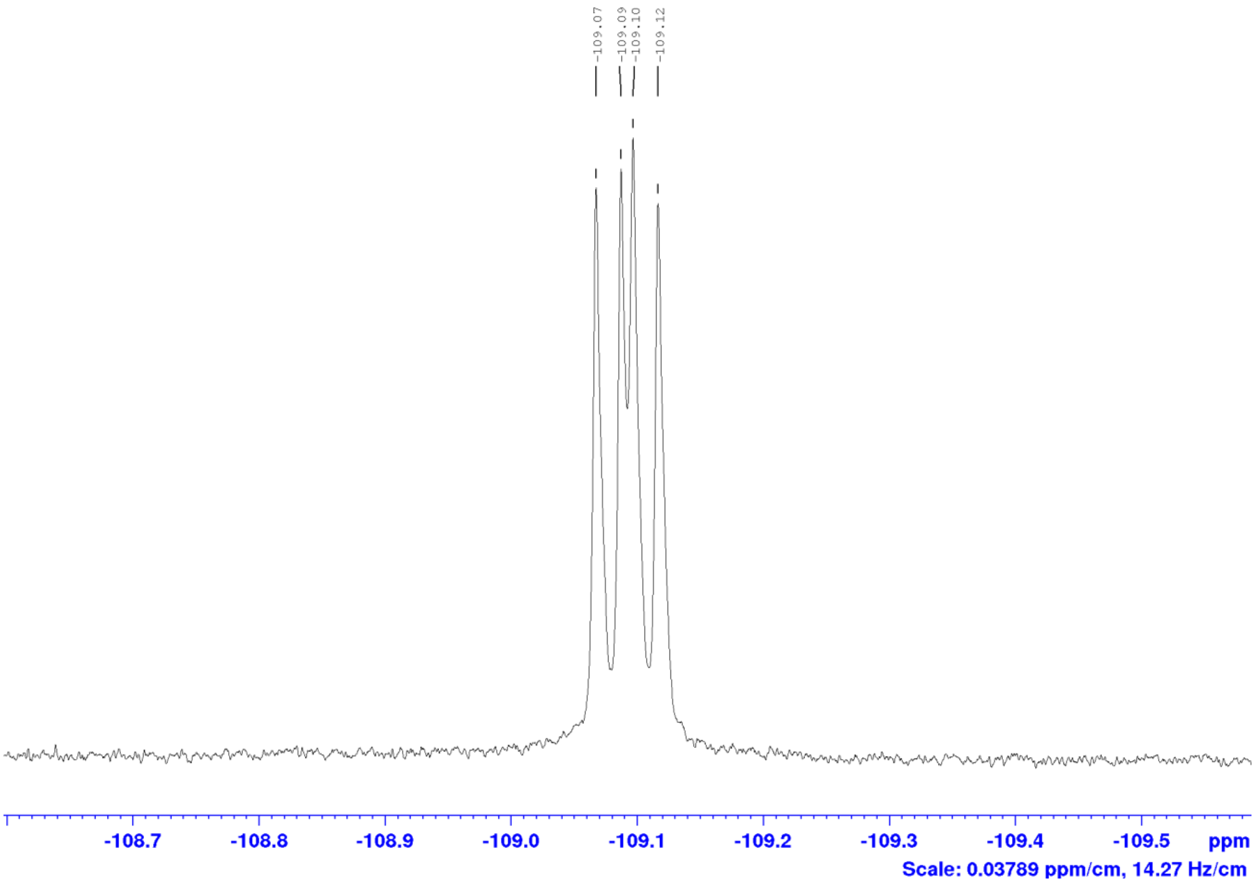

COSY (400 MHz, CDCl3) – **4**

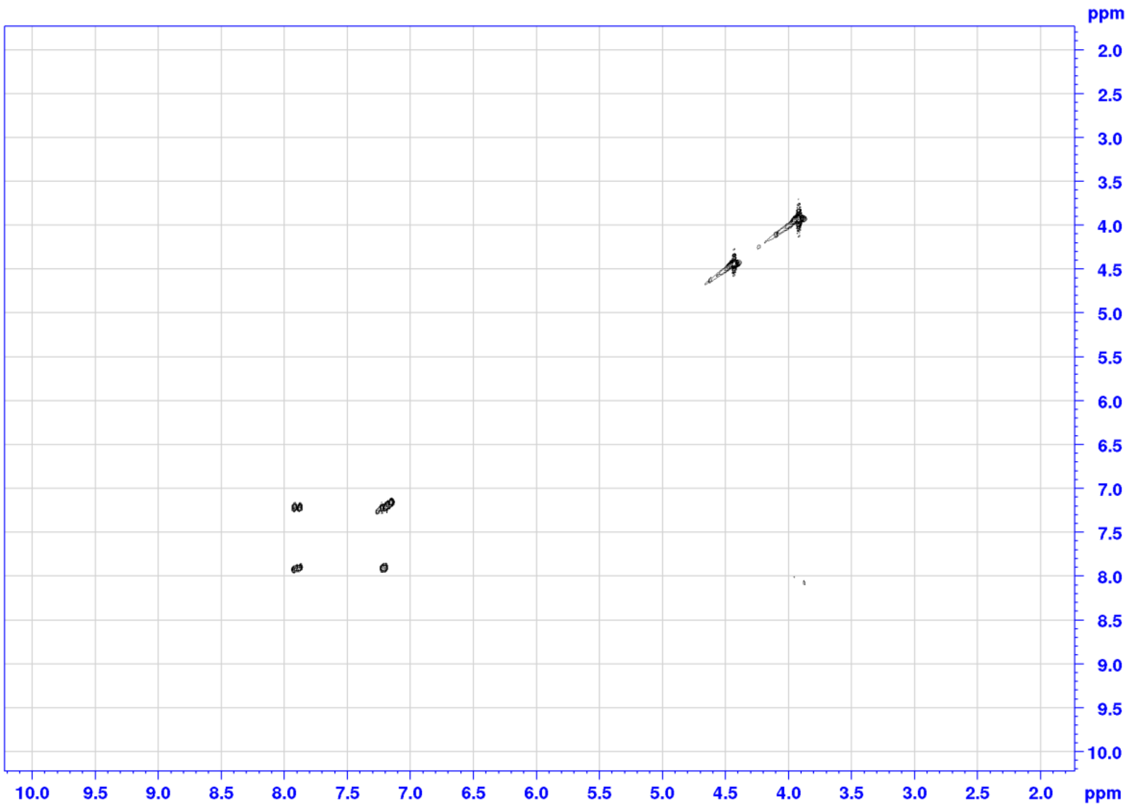

HMQC (100 MHz, CDCl3) – **4**

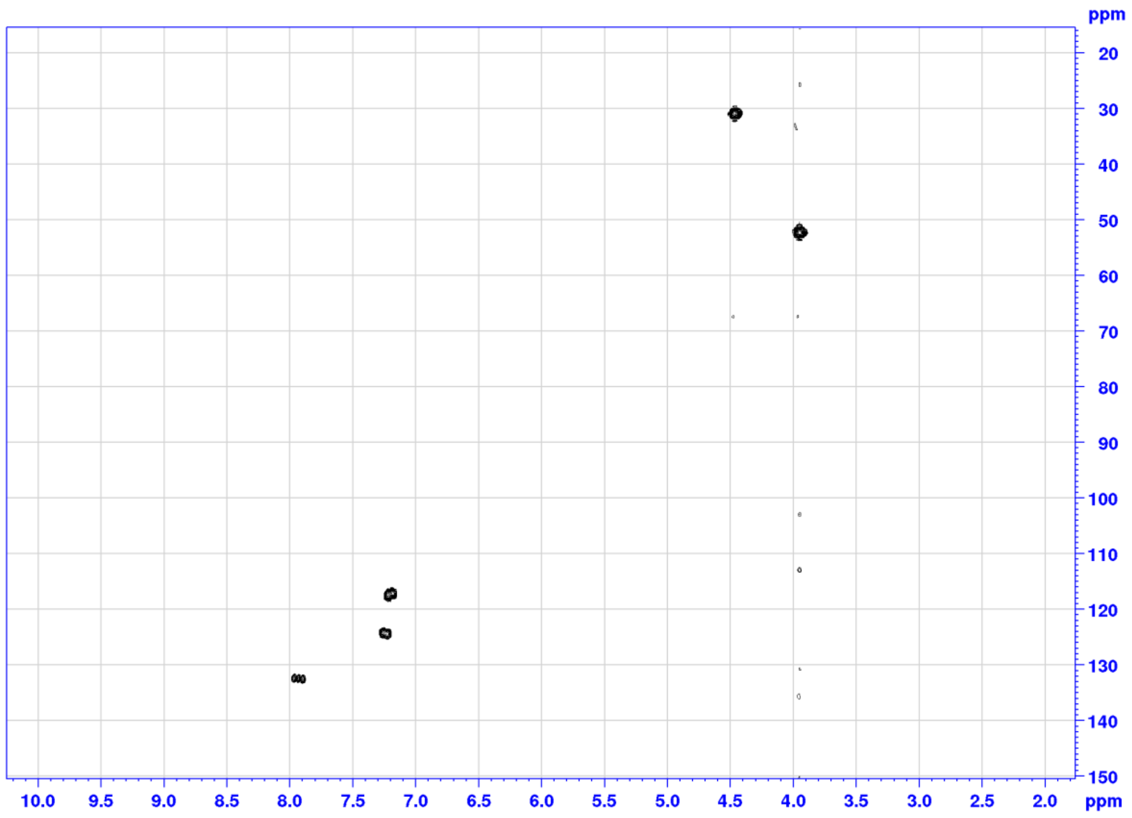

HMBC (100 MHz, CDCl3) – **4**

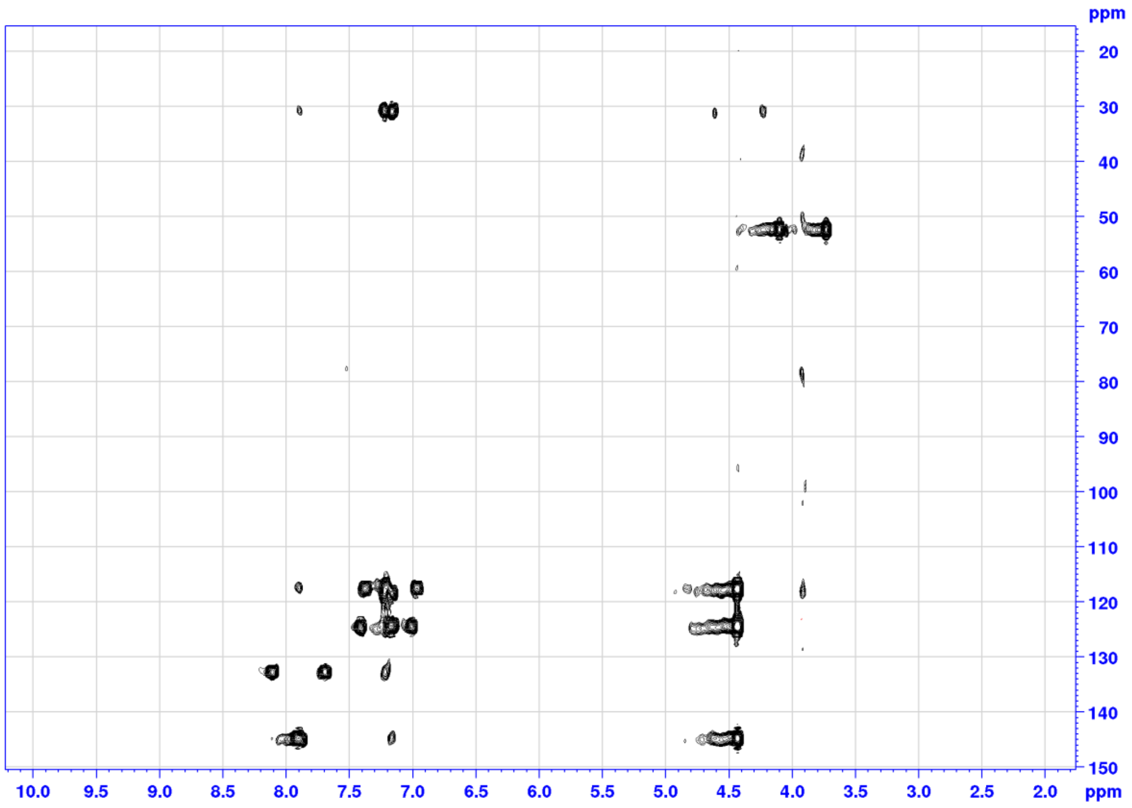

^{1}H-NMR (400 MHz, $CDCl_3$) – **5**

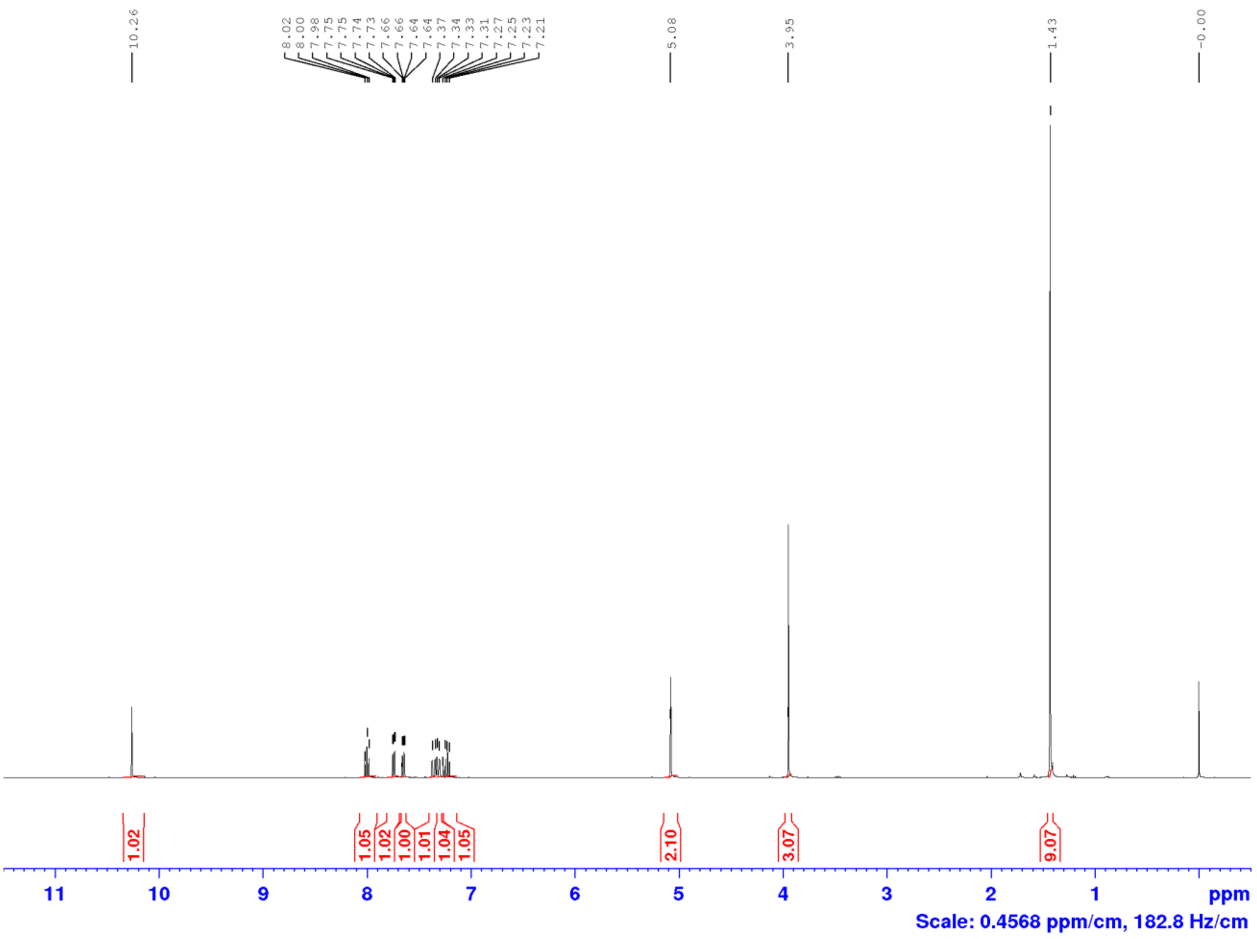

^{13}C-NMR (100 MHz, $CDCl_3$) – **5**

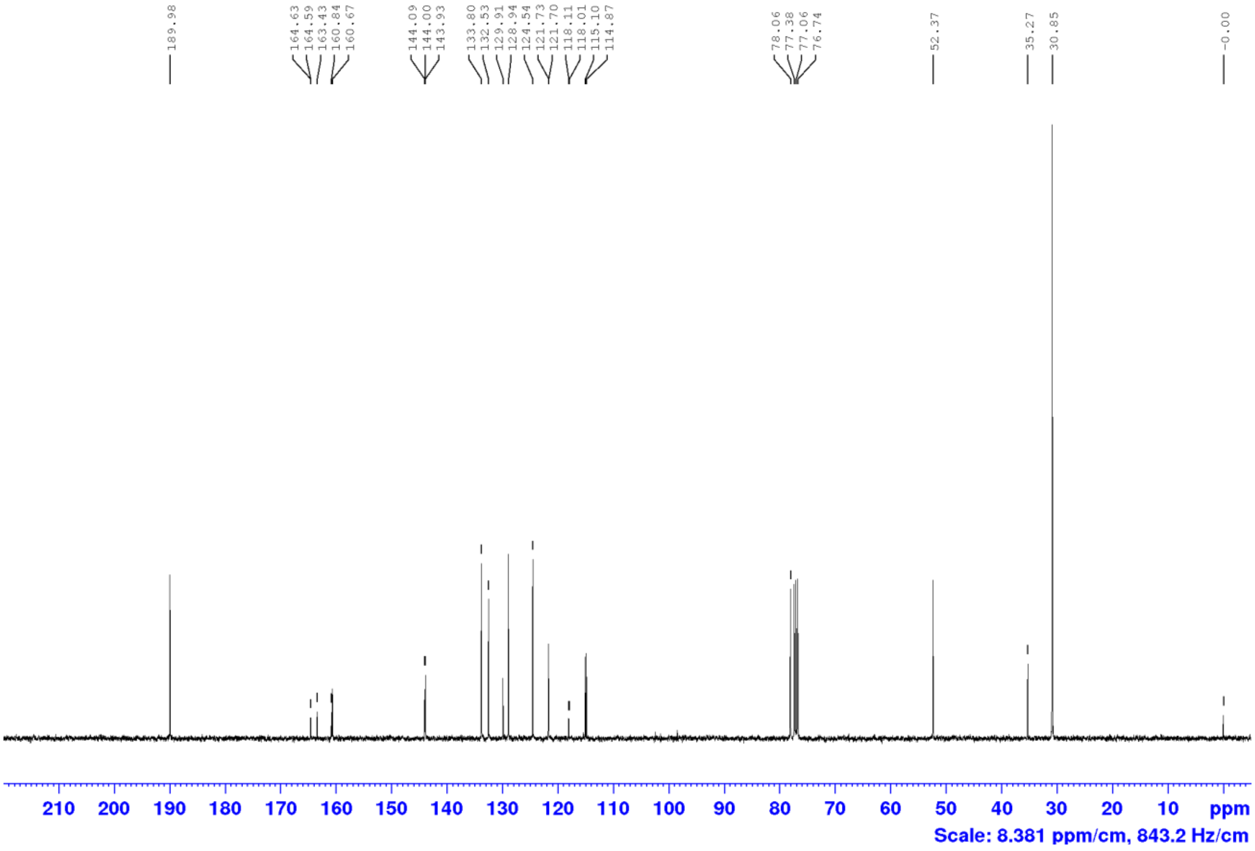

DEPT (100 MHz, $CDCl_3$) – **5**

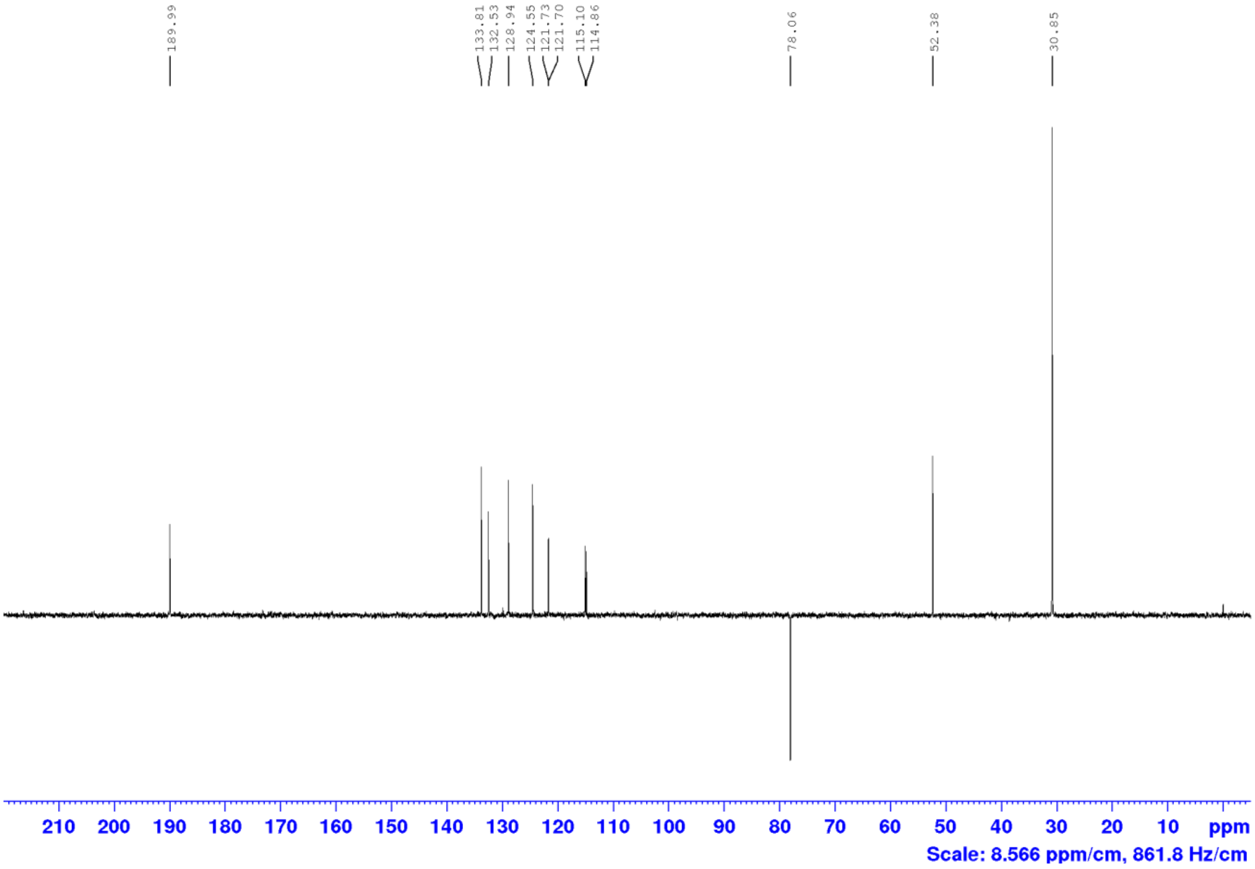

^{19}F-NMR (376 MHz, CDCl3) – **5**

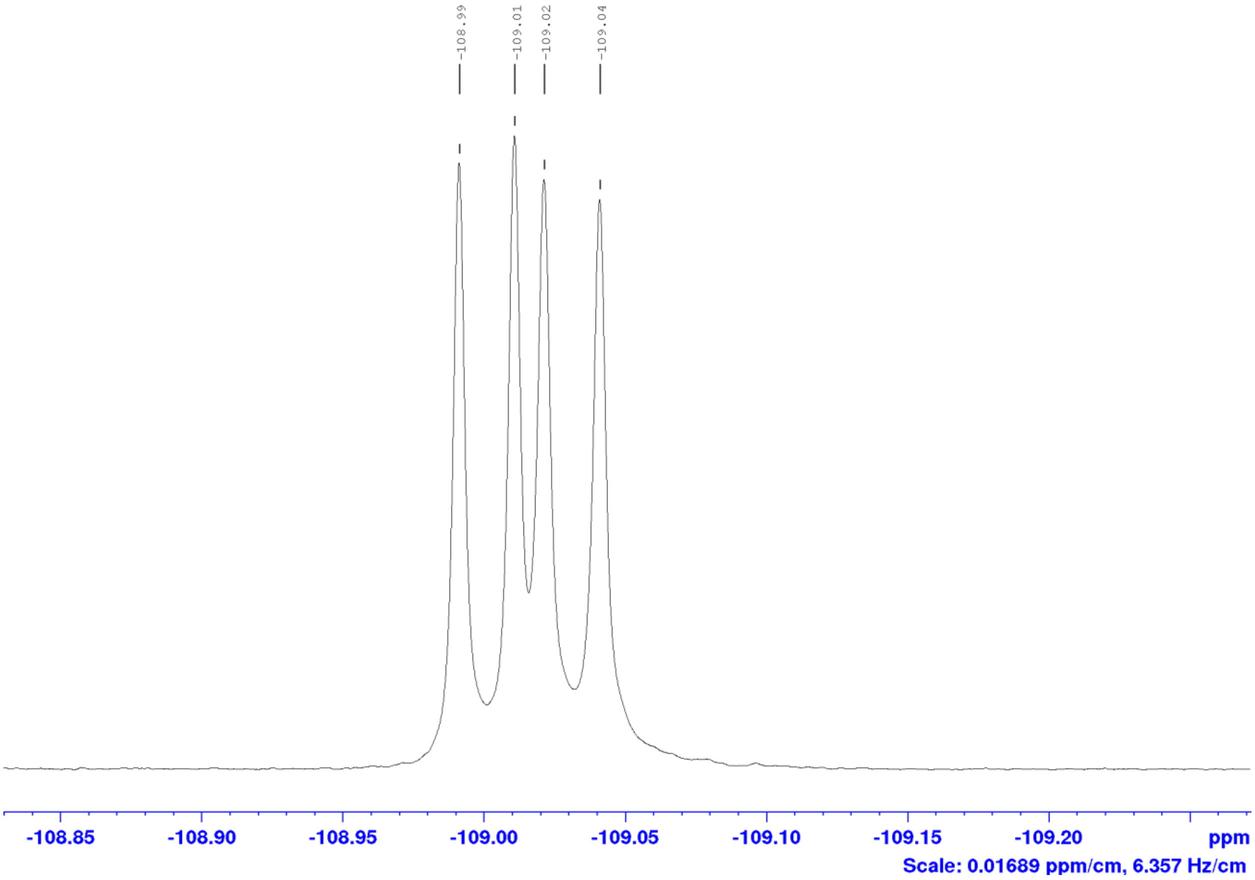

COSY (400 MHz, CDCl3) – **5**

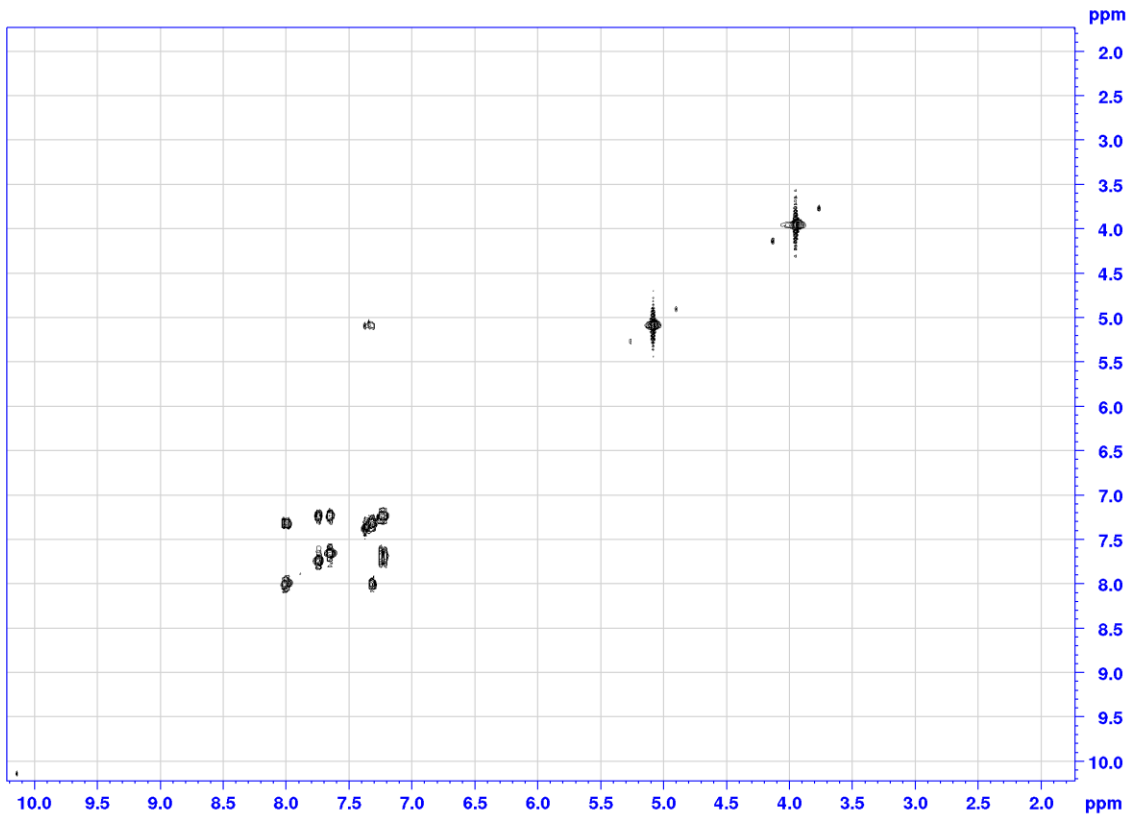

HMQC (100 MHz, CDCl3) – **5**

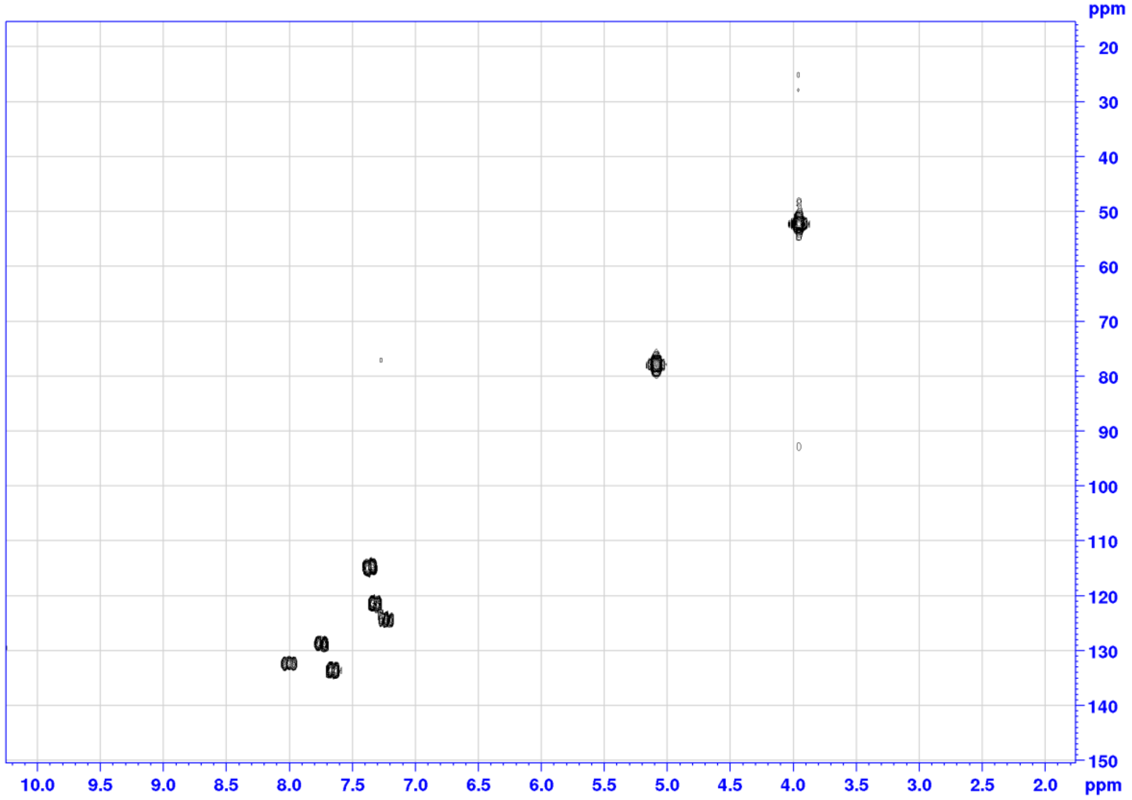

HMBC (100 MHz, CDCl3) – **5**

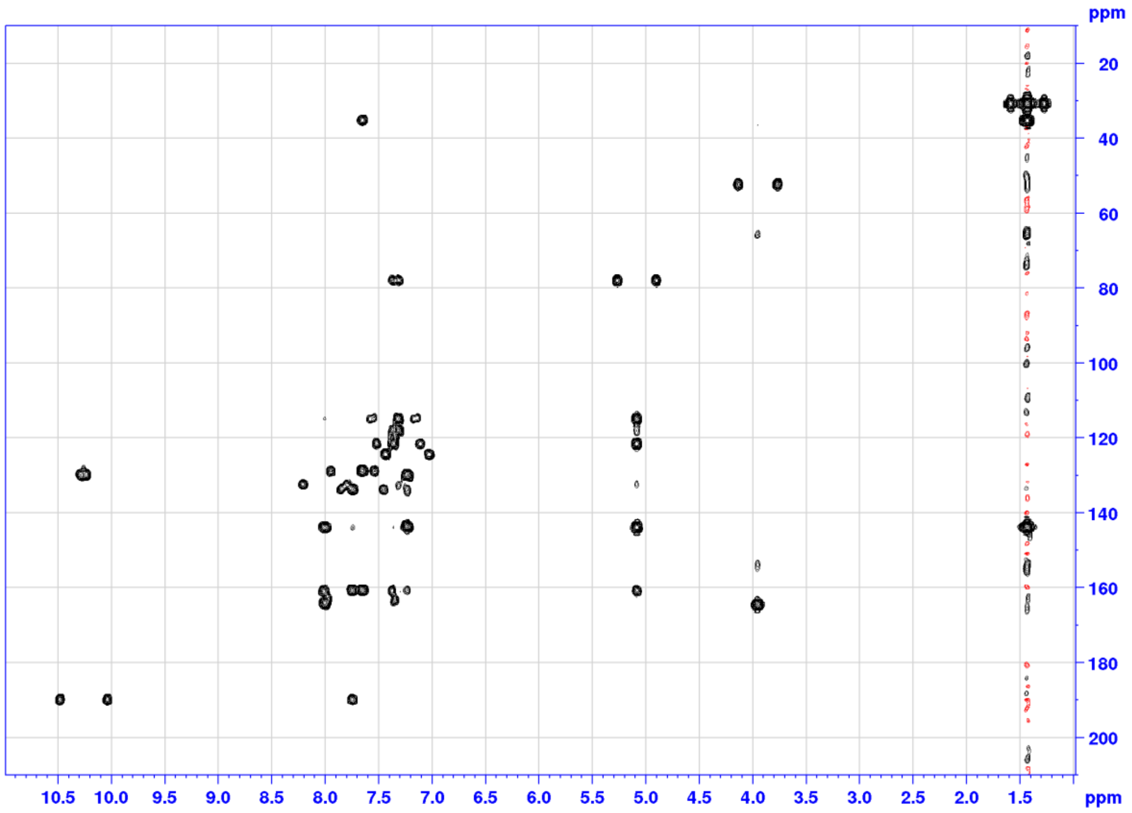

^{1}H-NMR (400 MHz, DMSO-d6) – **6 (EB-251)**

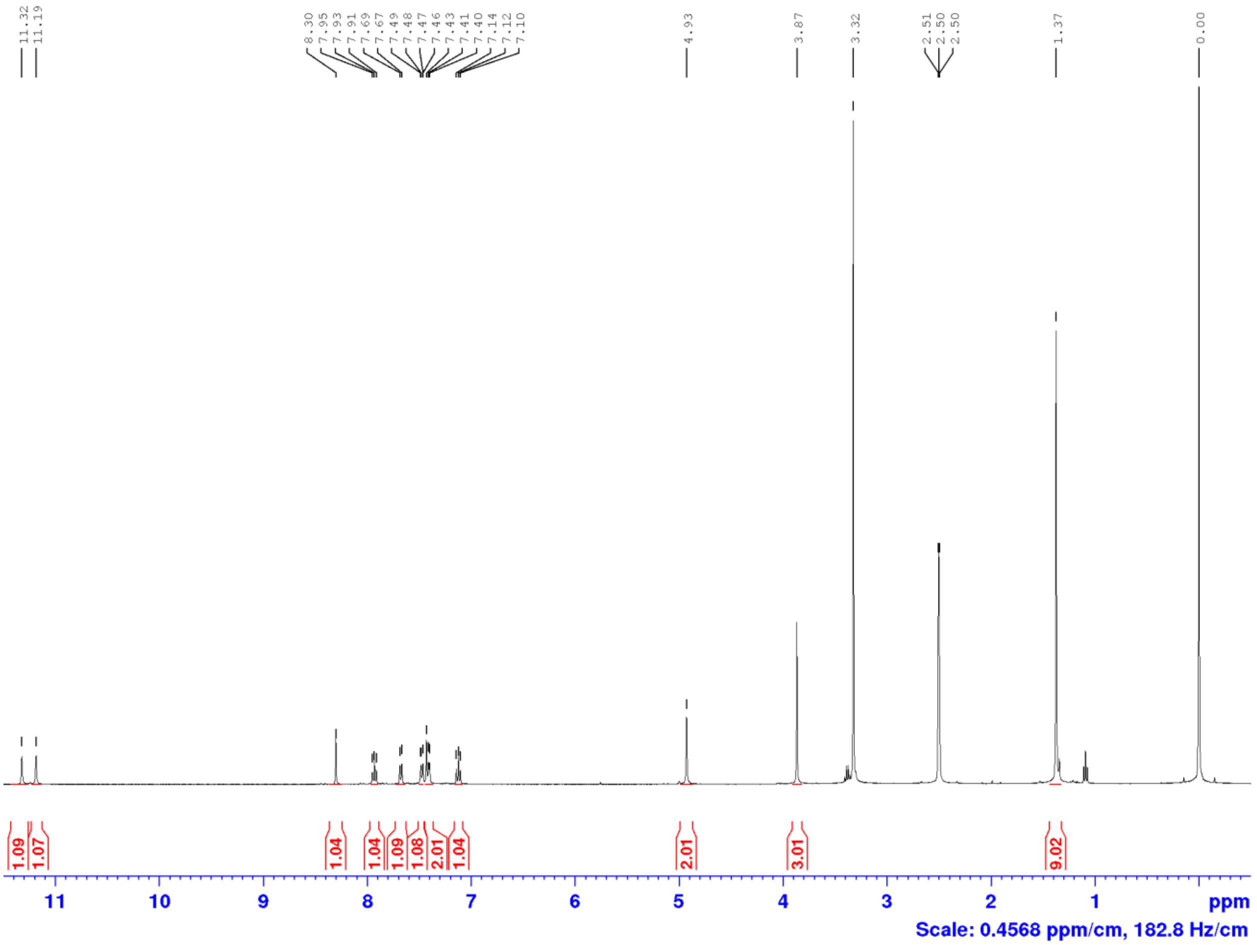

^{13}C-NMR (100 MHz, DMSO-d6) – **6 (EB-251)**

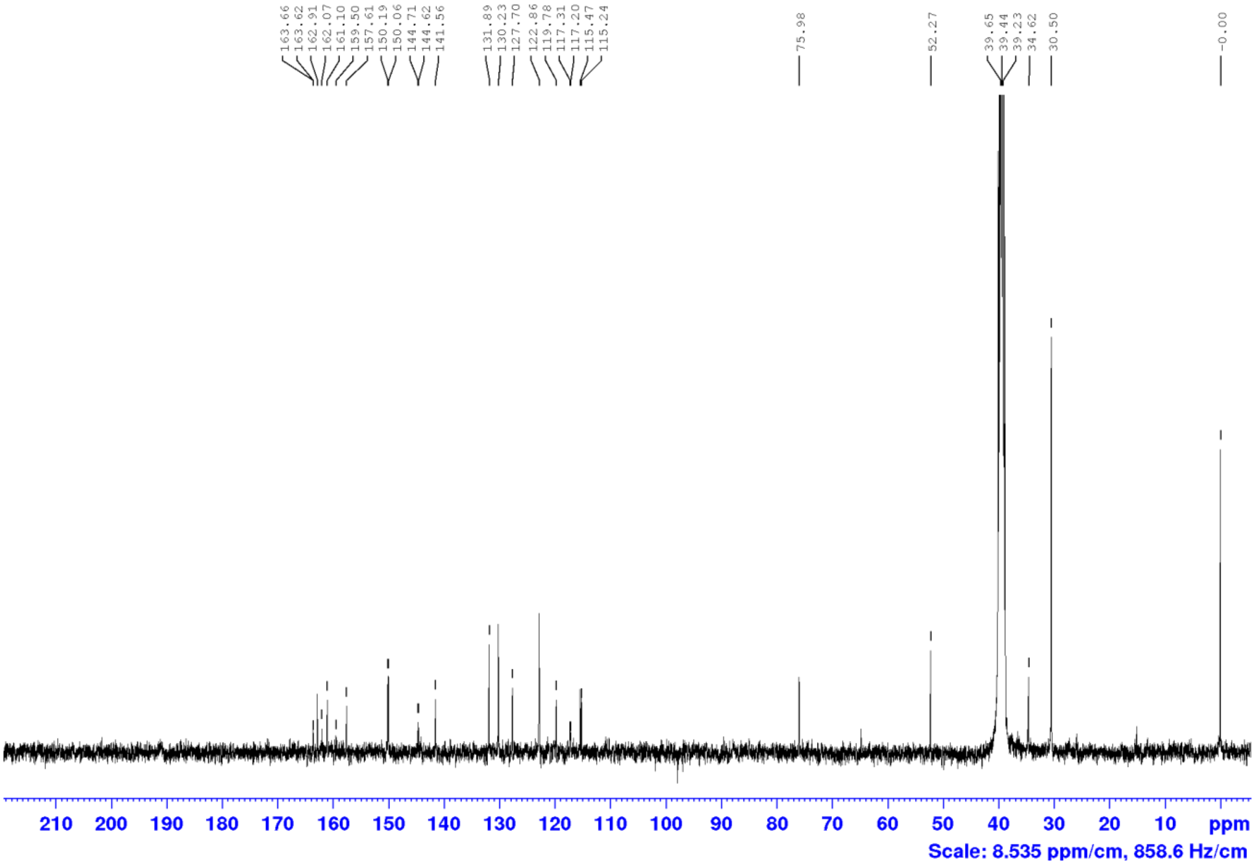

DEPT (100 MHz, DMSO-d6) – **6 (EB-251)**

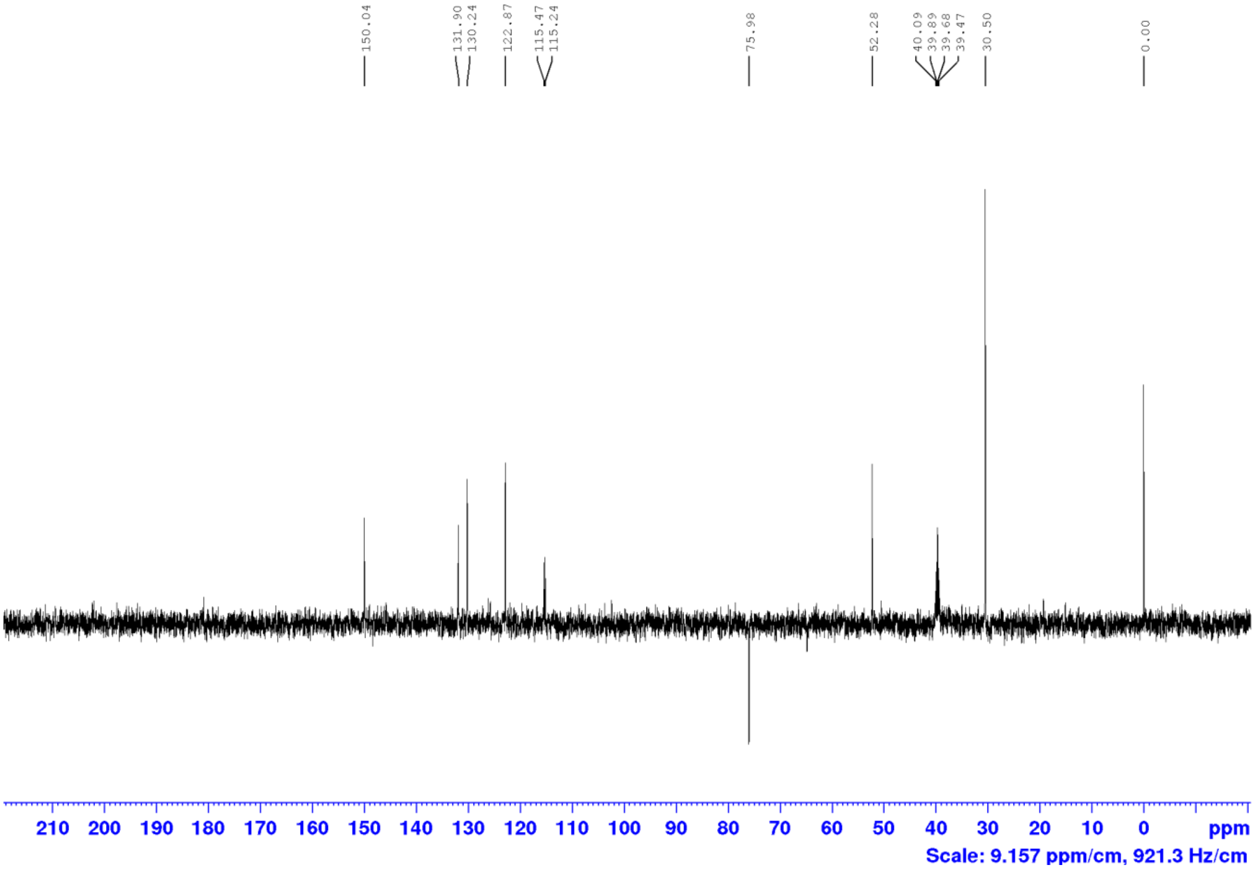

^{19}F-NMR (376 MHz, DMSO-d6) – **6 (EB-251)**

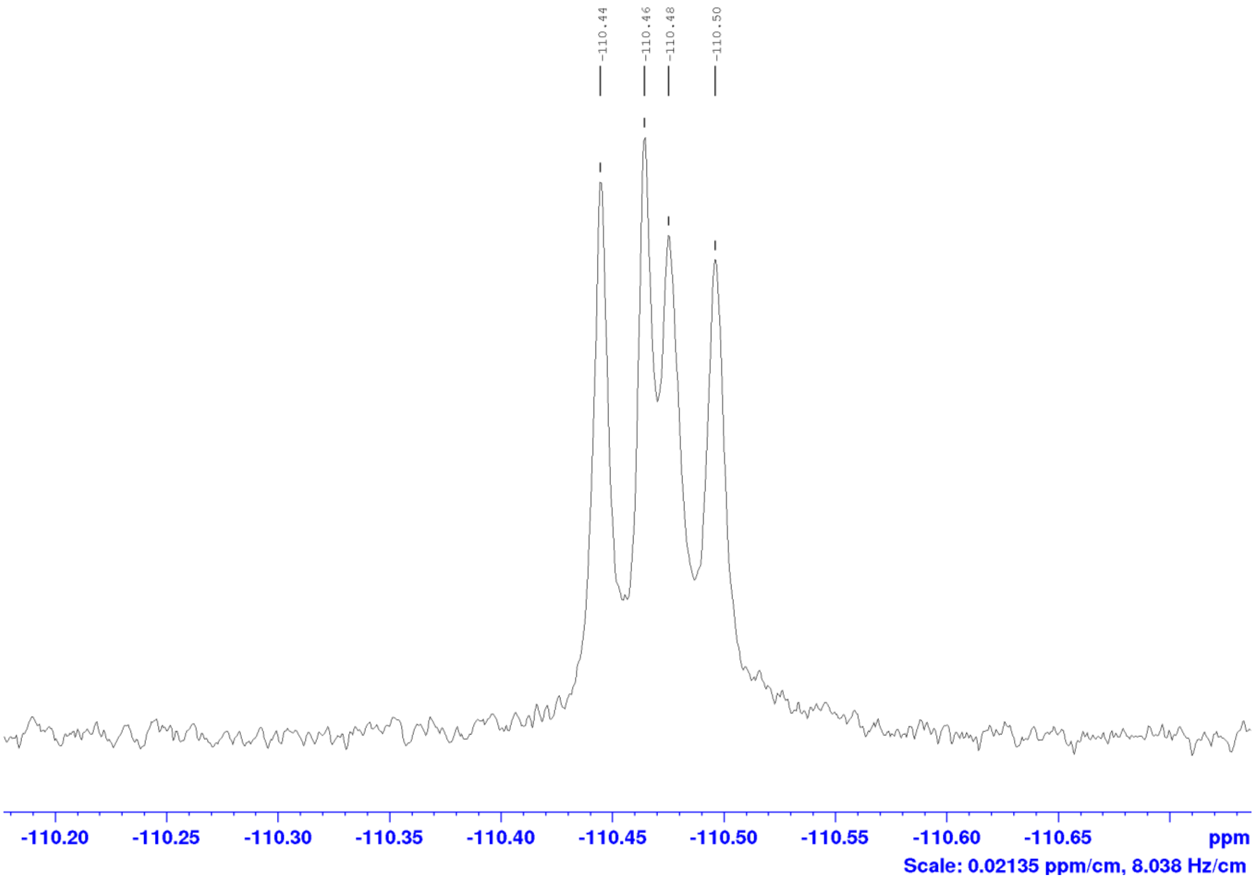

COSY (400 MHz, DMSO-d6) – **6 (EB-251)**

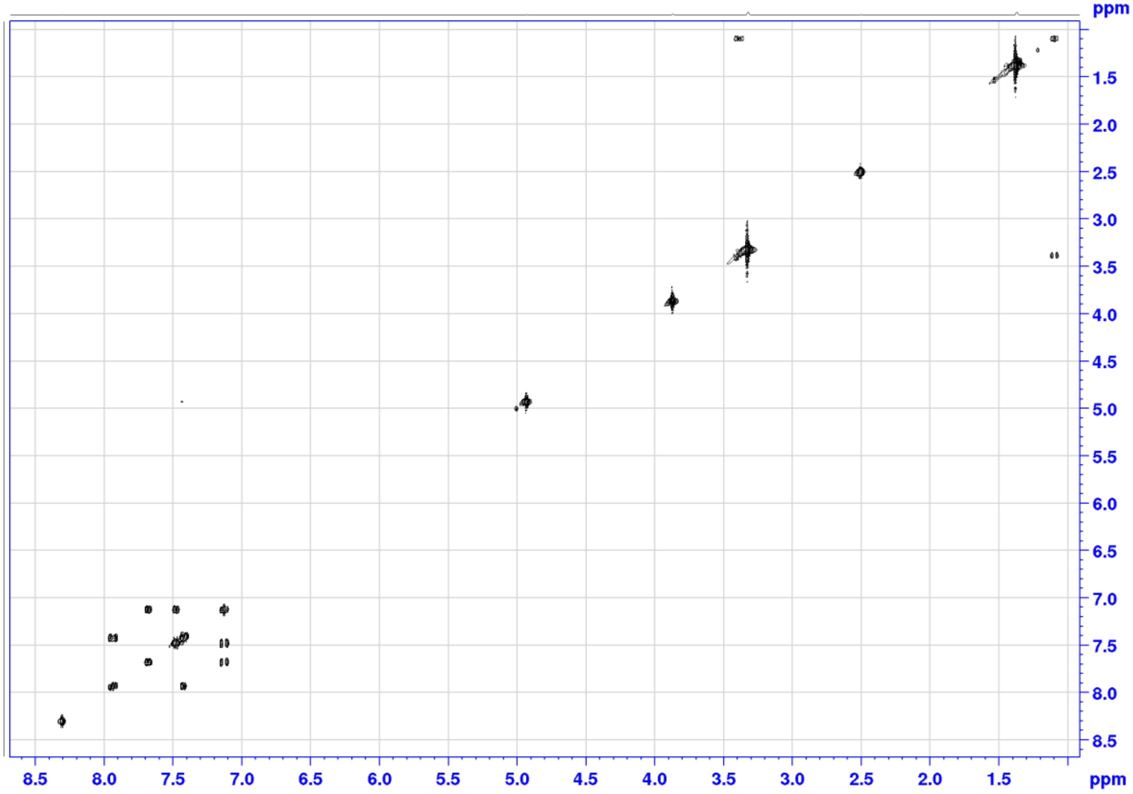

HMQC (100 MHz, DMSO-d6) – **6 (EB-251)**

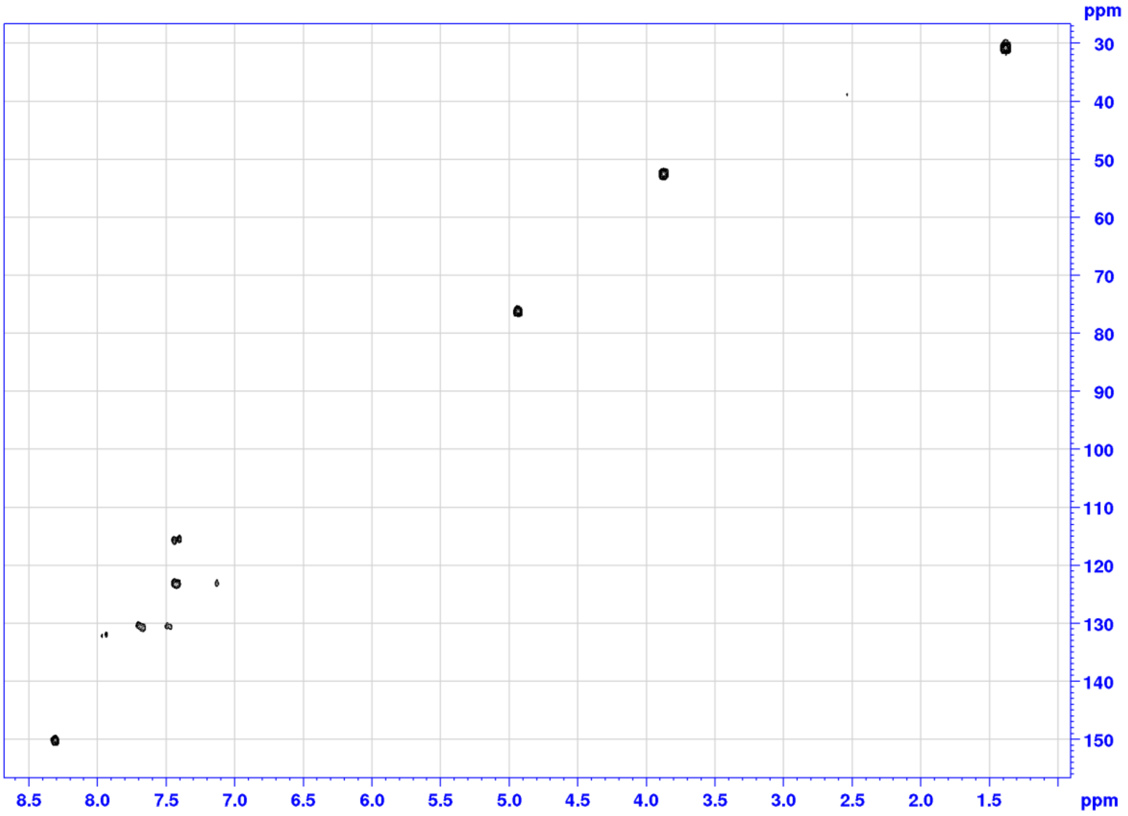

HMBC (100 MHz, DMSO-d6) – **6 (EB-251)**

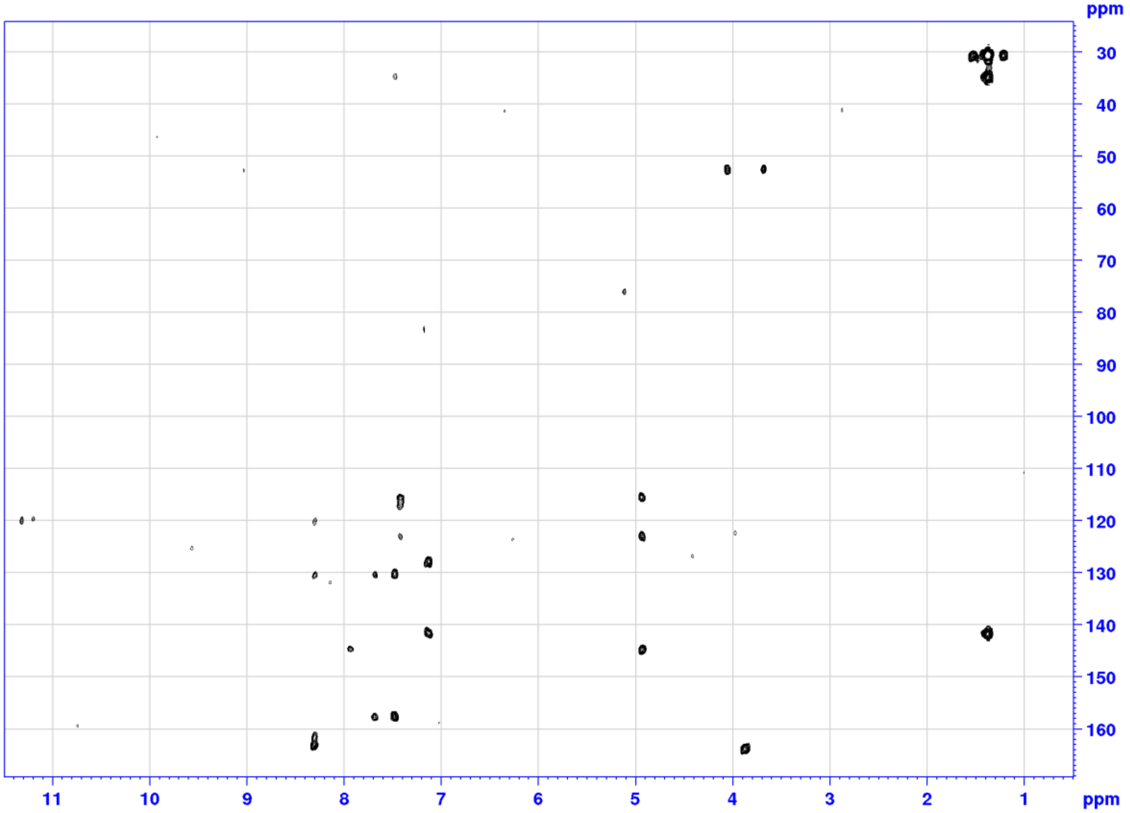

^{1}H-NMR (400 MHz, DMSO- d6) - **7**

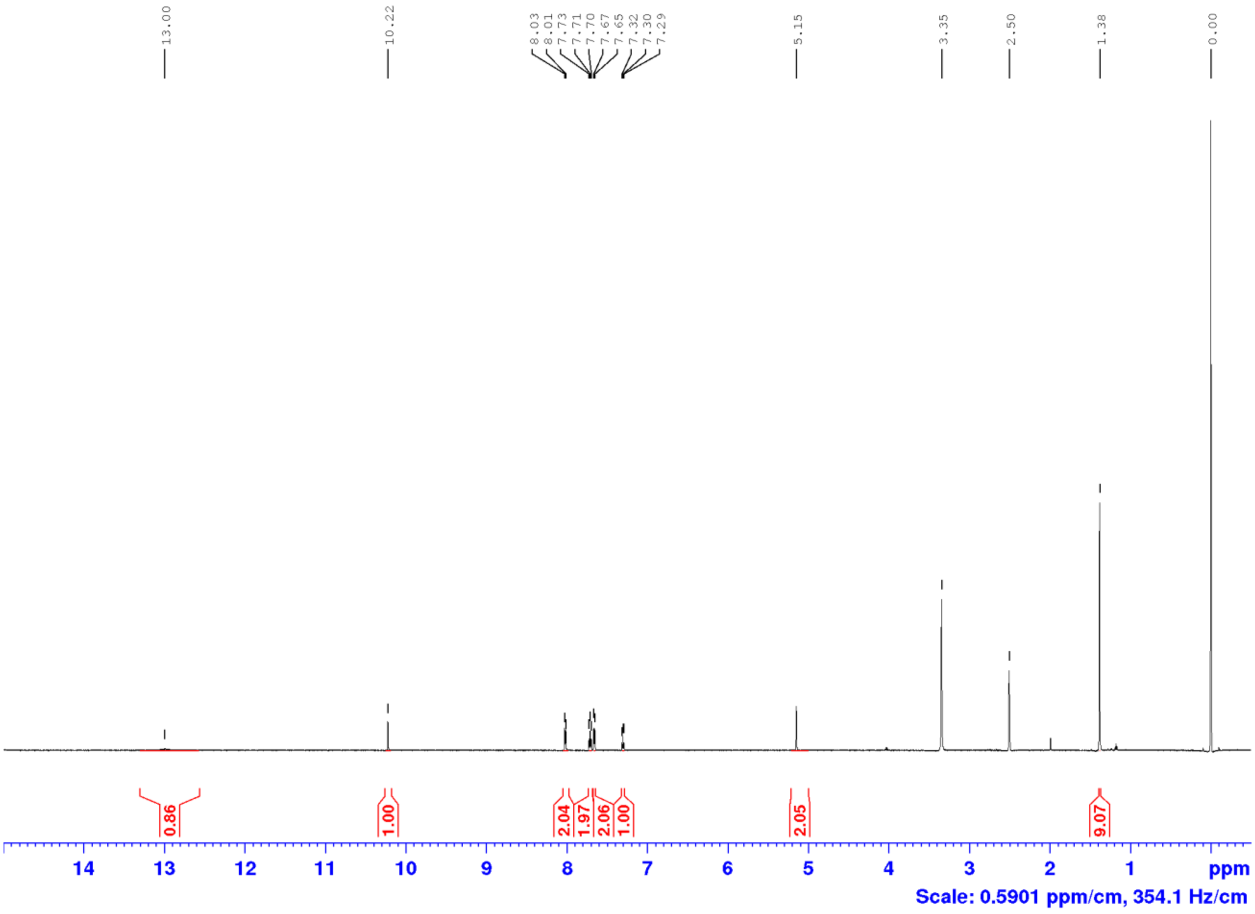

^{13}C-NMR (100 MHz, DMSO- d6) - **7**

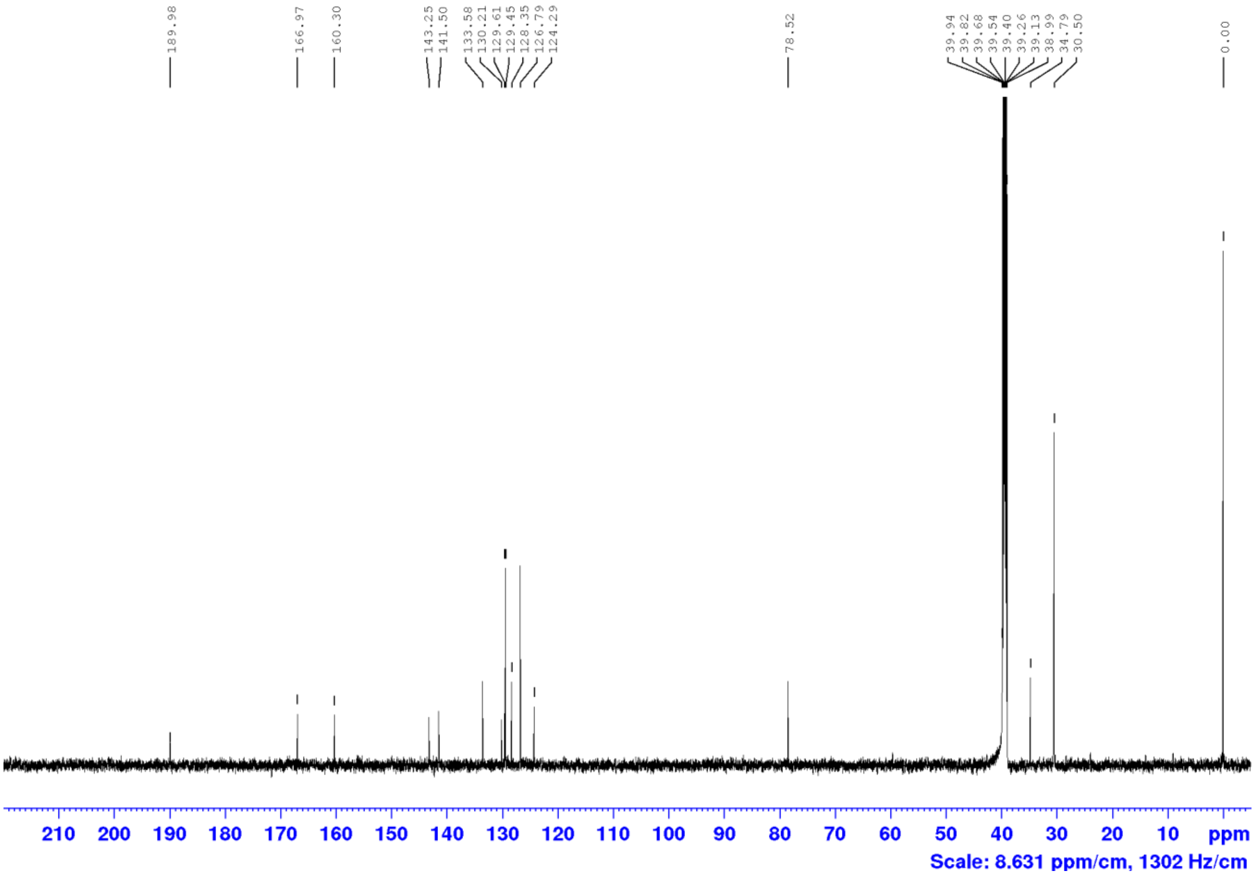

DEPT (100 MHz, DMSO-d6) - **7**

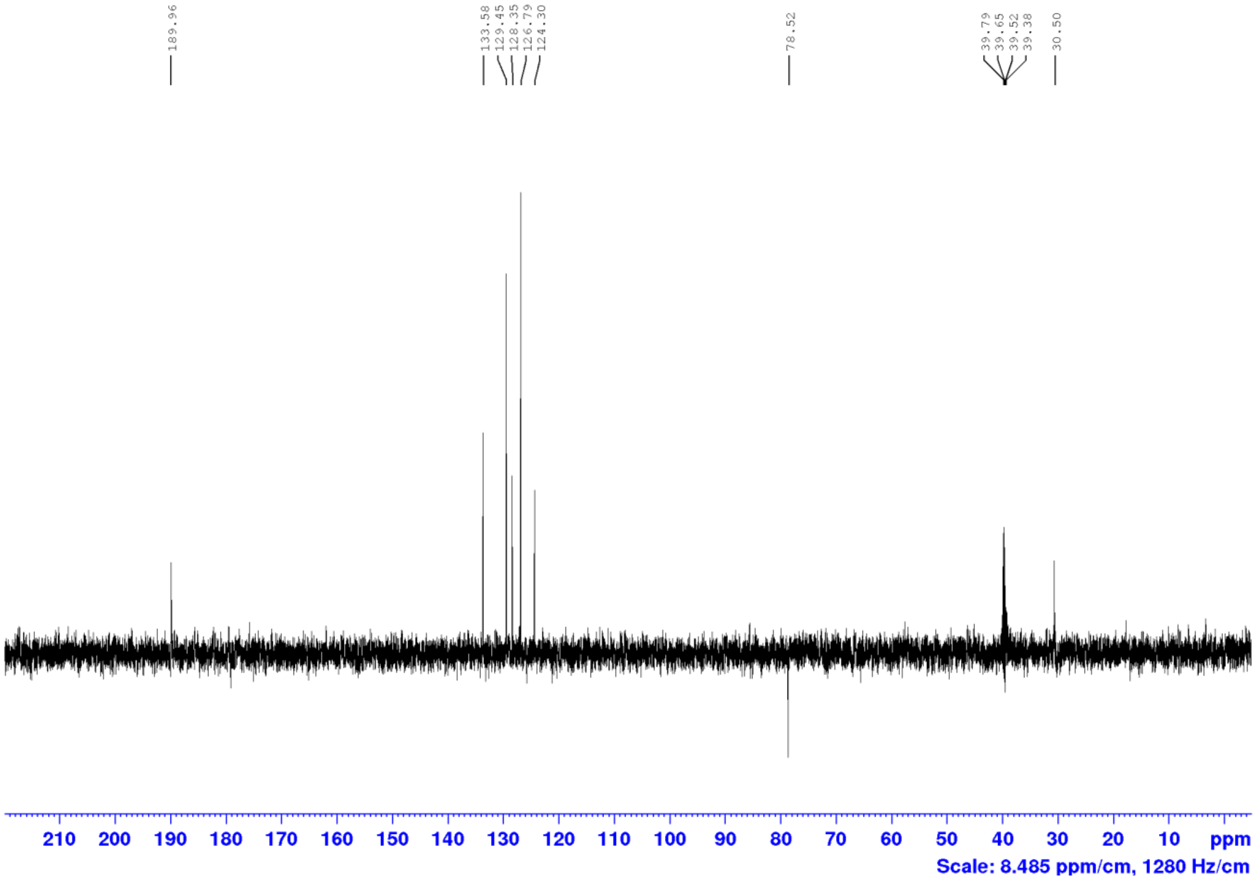

^{1}H-NMR (700 MHz, DMSO-d6) – **8 (EB-269)**

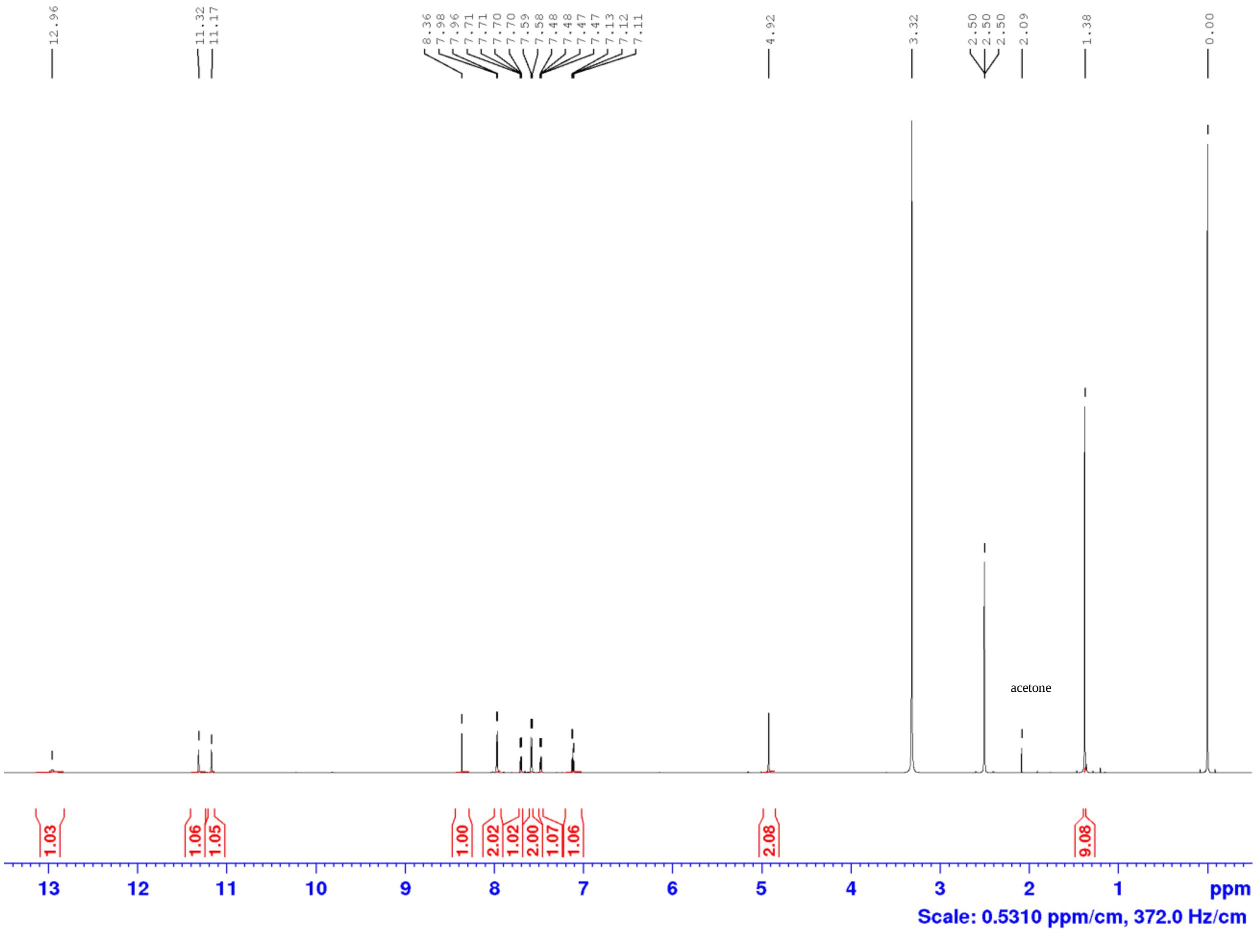

^{13}C-NMR (176 MHz, DMSO-d6) – **8 (EB-269)**

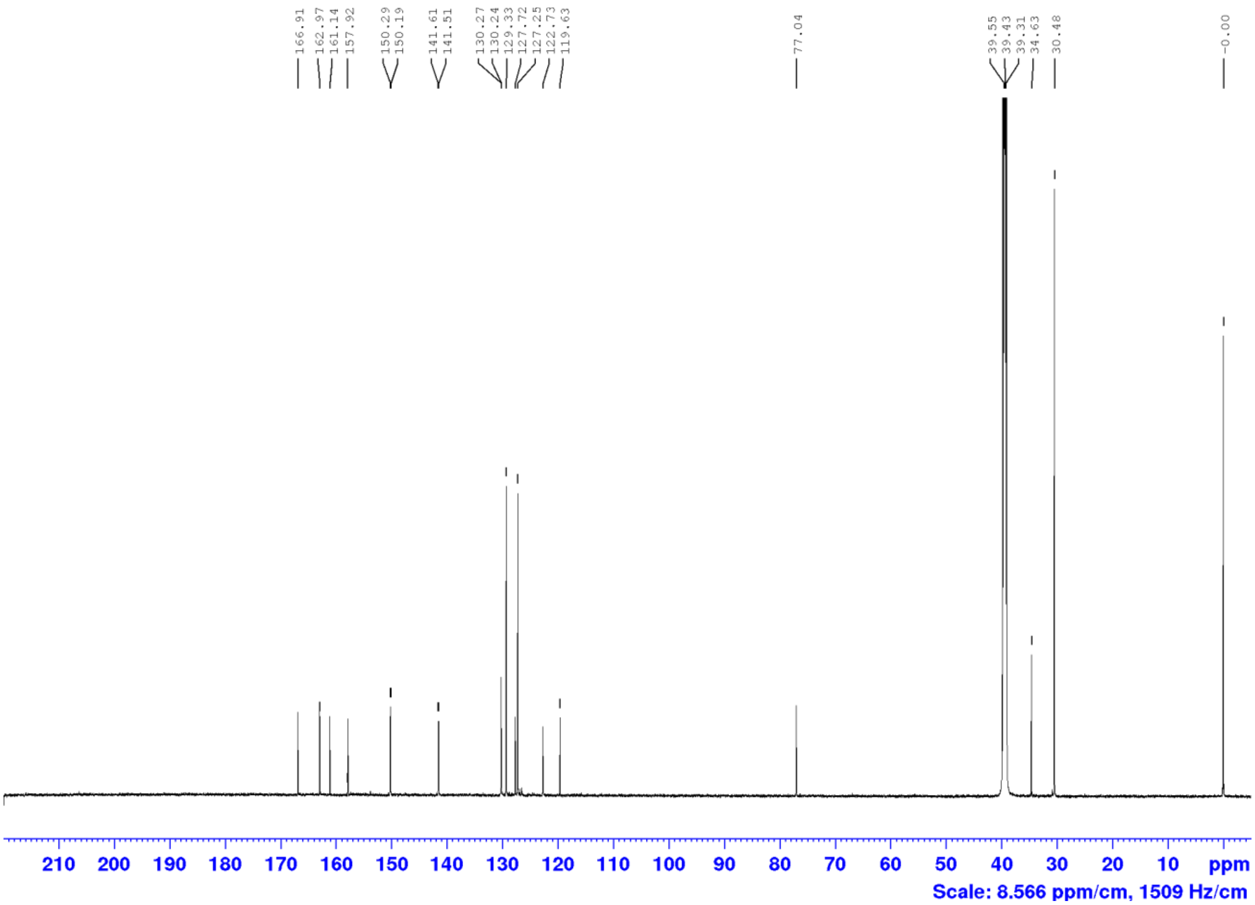

DEPT (176 MHz, DMSO- d6) – **8 (EB-269)**

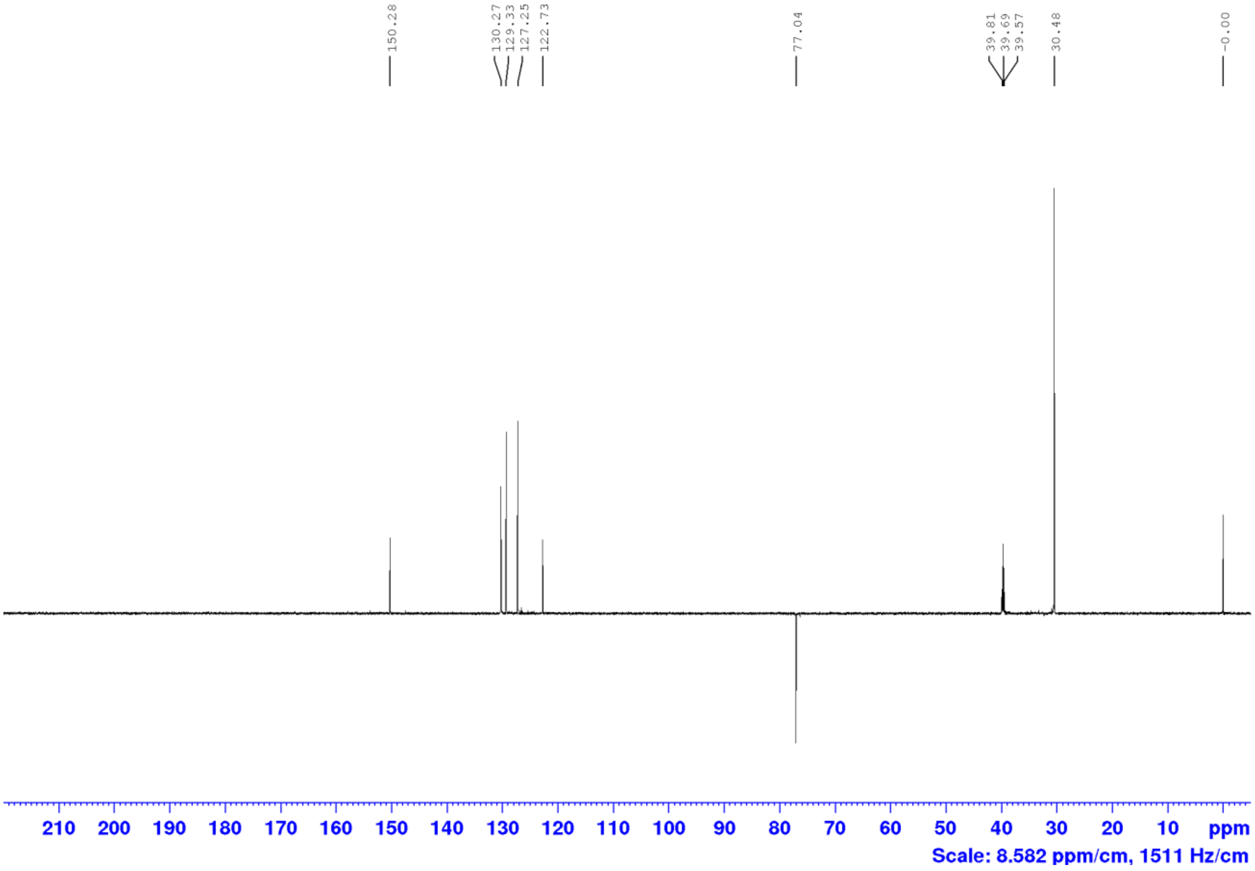

COSY (700 MHz, DMSO- d6)– **8 (EB-269)**

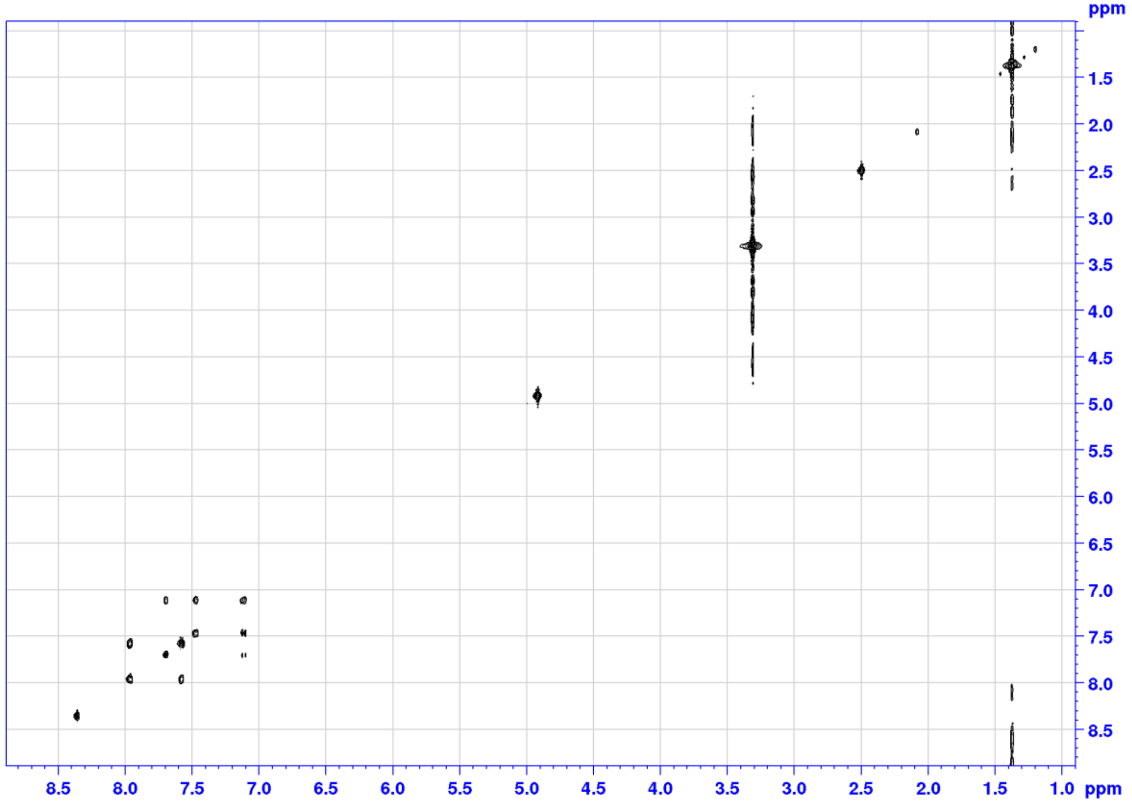

HMQC (176 MHz, DMSO- d6) – **8 (EB-269)**

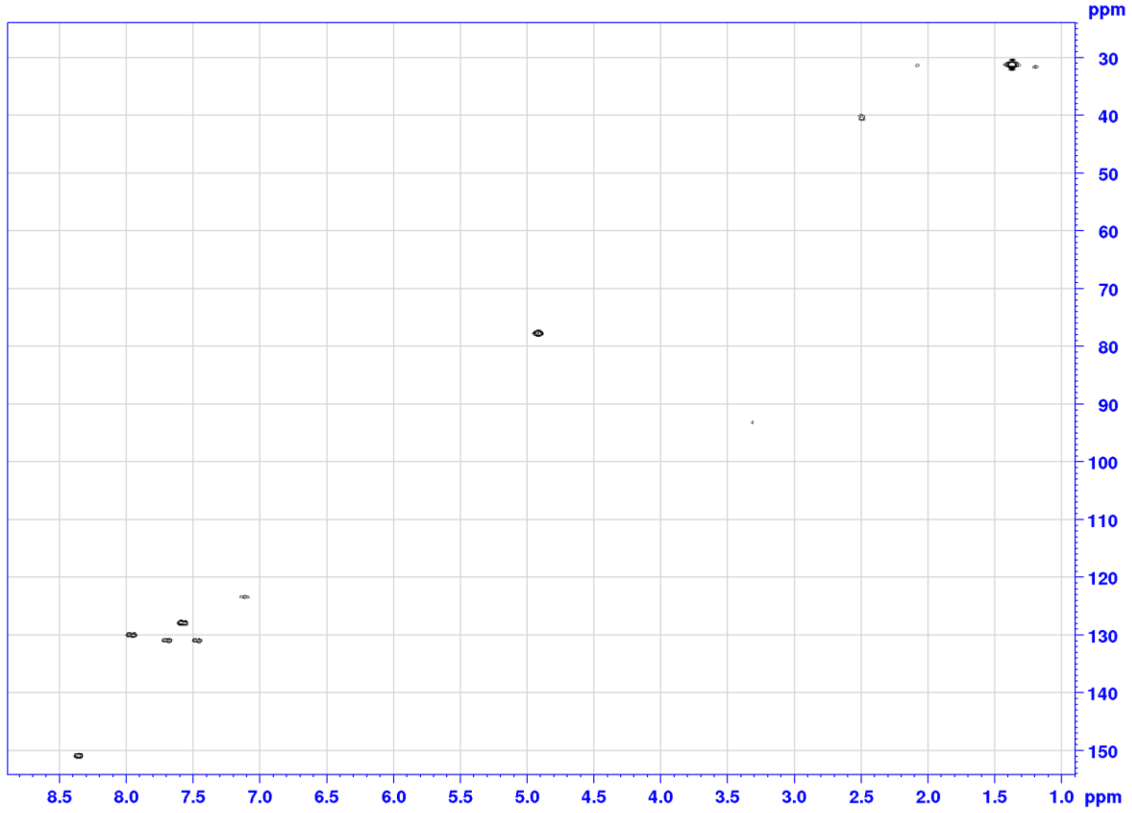

HMBC (176 MHz, DMSO-d6)– **8 (EB-269)**

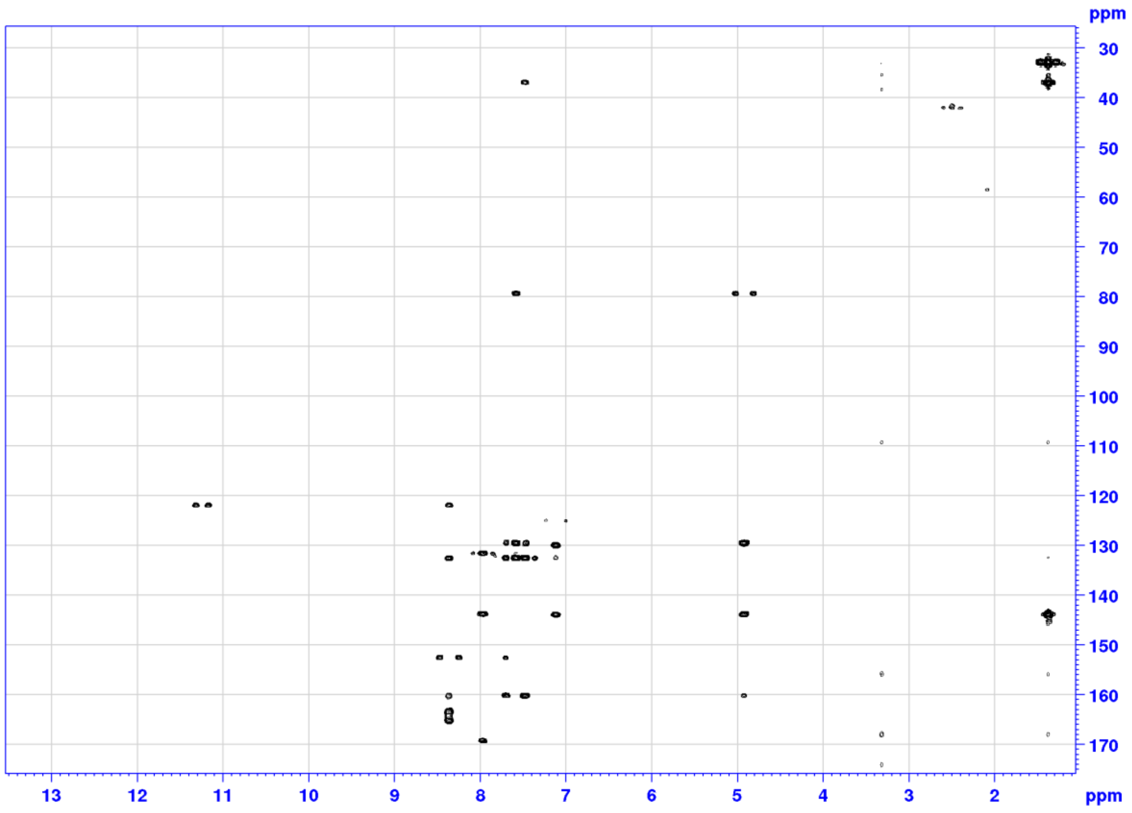

HMBC-N (700 MHz, DMSO-d6) – **8 (EB-269)**

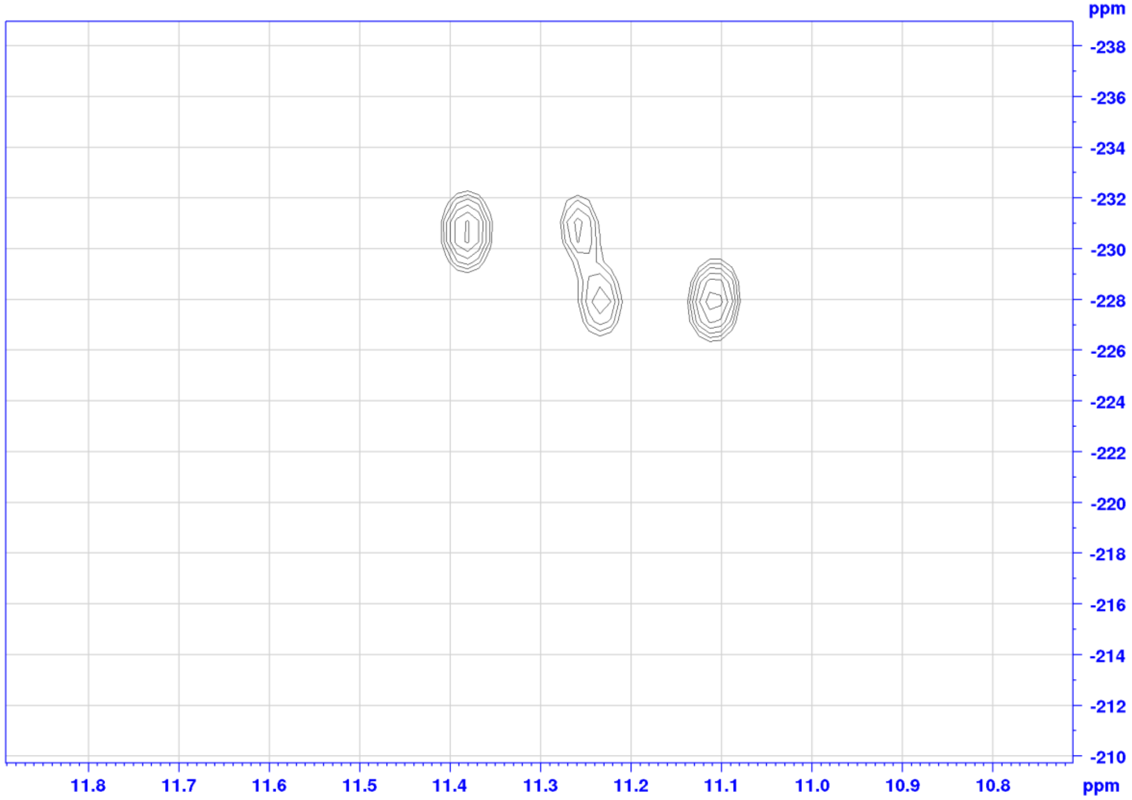

Analytical chemistry: Mass spectroscopy data

1

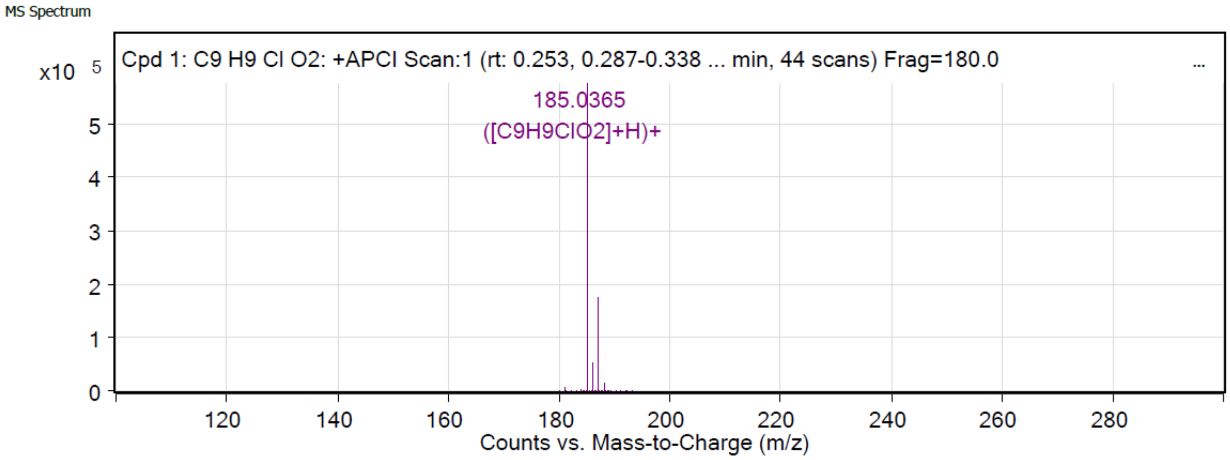

MS Spectrum Peak List

m/z	Calc *m/z*	Diff(ppm)	z	Abund	Formula	Ion
185.0365	185.0364	-0.62	1	575498.13	C9H9ClO2	(M+H)+
186.0395	186.0398	1.54	1	52743.98	C9H9ClO2	(M+H)+
187.0335	187.0337	0.58	1	173196.84	C9H9ClO2	(M+H)+
188.0365	188.037	2.54	1	16467.58	C9H9ClO2	(M+H)+
189.0386	189.0391	2.29	1	1450.51	C9H9ClO2	(M+H)+

2

MS Spectrum Peak List

m/z	Calc *m/z*	Diff(ppm)	z	Abund	Formula	Ion
271.0958	271.0965	2.41	1	309444.03	C16H14O4	(M+H)+
272.099	272.0999	3.19	1	50296.23	C16H14O4	(M+H)+
273.1014	273.1024	3.52	1	5547.79	C16H14O4	(M+H)+
274.1038	274.105	4.41	1	501.74	C16H14O4	(M+H)+
293.0778	293.0784	2.28	1	675643.75	C16H14O4	(M+Na)+
294.081	294.0818	2.72	1	101156.87	C16H14O4	(M+Na)+
295.0834	295.0843	3.14	1	12159.47	C16H14O4	(M+Na)+
296.0864	296.0869	1.97	1	1245.39	C16H14O4	(M+Na)+
558.2094	558.2122	5.12	1	171.77	C16H14O4	(2M+NH4)+
559.2182	559.2156	-4.68	1	55.78	C16H14O4	(2M+NH4)+
563.1673	563.1676	0.57	1	761669.19	C16H14O4	(2M+Na)+
564.1708	564.171	0.39	1	252038.8	C16H14O4	(2M+Na)+
565.1731	565.1739	1.34	1	48307.5	C16H14O4	(2M+Na)+
566.1756	566.1766	1.81	1	7528.32	C16H14O4	(2M+Na)+
567.1778	567.1793	2.72	1	1052.7	C16H14O4	(2M+Na)+
568.1455	568.1819	64.19	1	2225.03	C16H14O4	(2M+Na)+

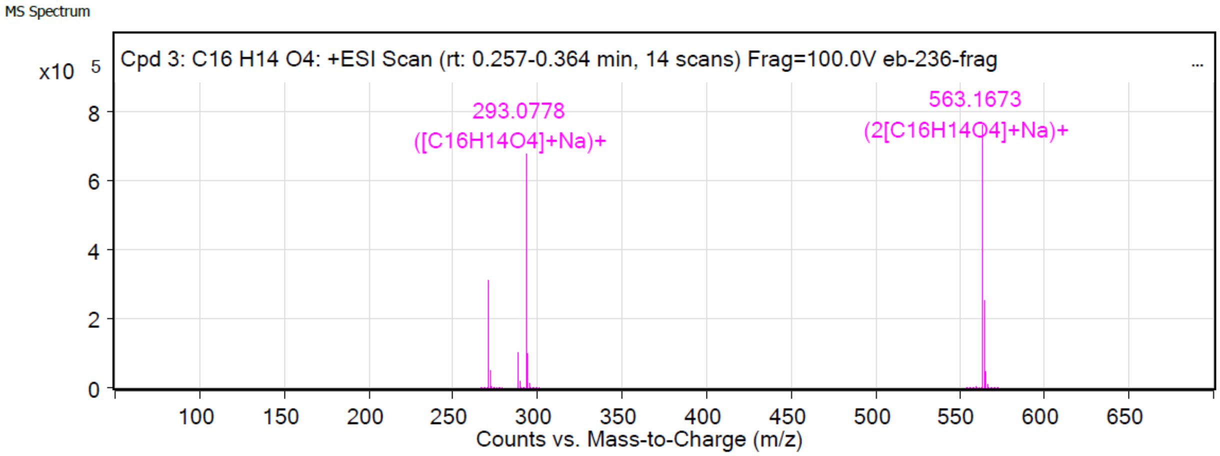

3 (EB-237)

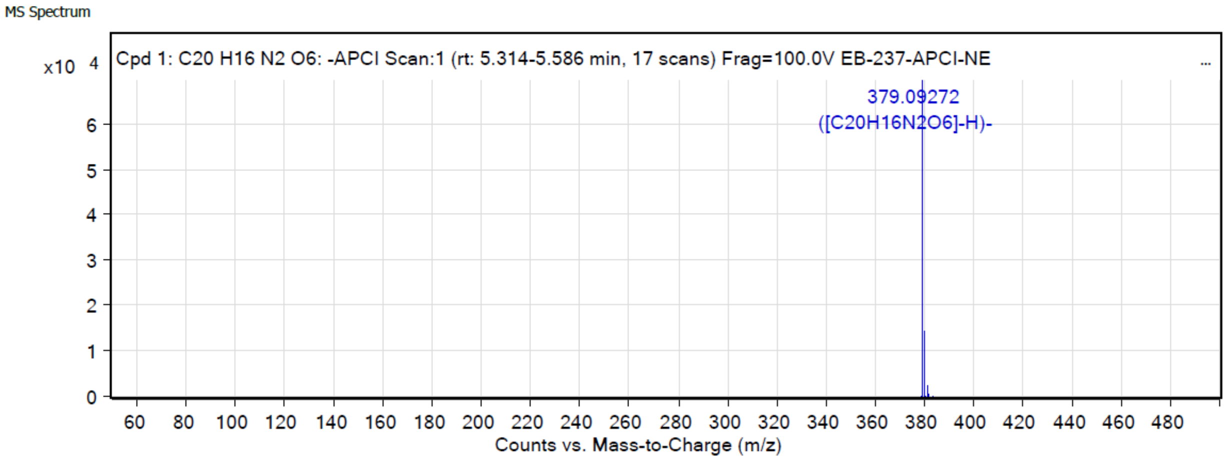

MS Spectrum Peak List

m/z	Calc *m/z*	Diff(ppm)	z	Abund	Formula	Ion
379.09272	379.09356	2.21	1	69552.61	C20H16N2O6	(M-H)-
380.09589	380.09674	2.23	1	14256.08	C20H16N2O6	(M-H)-
381.09818	381.09921	2.72	1	2165.63	C20H16N2O6	(M-H)-
382.10135	382.10178	1.11	1	216.35	C20H16N2O6	(M-H)-

4

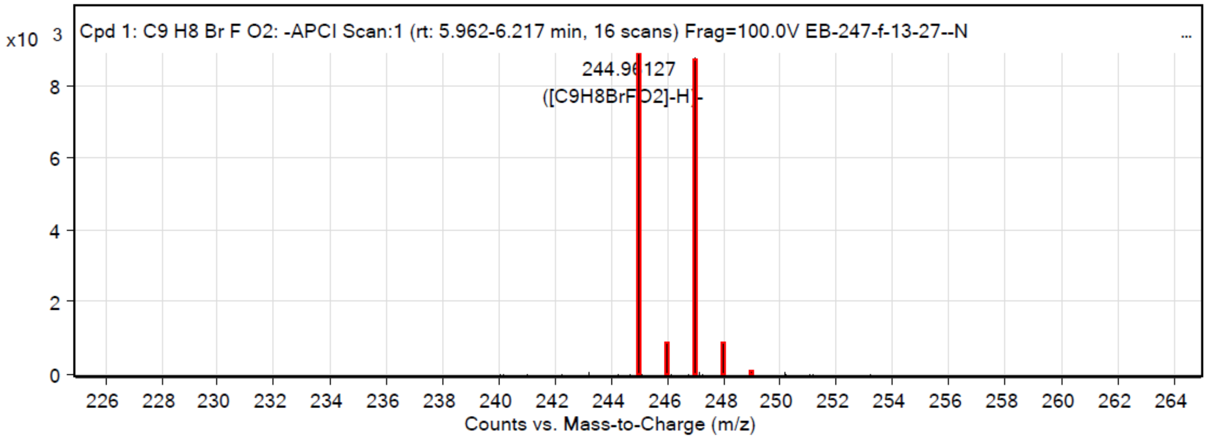

MS Spectrum Peak List

m/z	Calc *m/z*	Diff(ppm)	z	Abund	Formula	Ion
244.96127	244.96189	2.56	1	8899.18	C9H8BrFO2	(M-H)-
245.96506	245.96528	0.89	1	843.28	C9H8BrFO2	(M-H)-
246.95976	246.95991	0.61	1	8763.95	C9H8BrFO2	(M-H)-
247.96325	247.96327	0.09	1	868.66	C9H8BrFO2	(M-H)-
248.96441	248.96542	4.06	1	52.15	C9H8BrFO2	(M-H)-

5

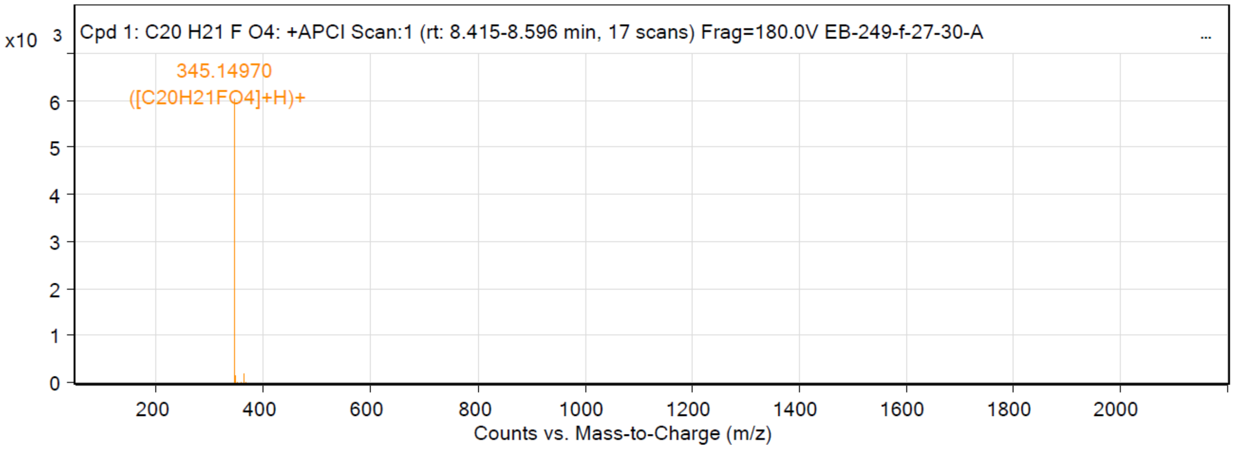

MS Spectrum Peak List

m/z	Calc *m/z*	Diff(ppm)	z	Abund	Formula	Ion
345.1497	345.14966	-0.11	1	6034.93	C20H21FO4	(M+H)+
346.15284	346.15306	0.63	1	1268.16	C20H21FO4	(M+H)+
347.15593	347.15579	-0.41	1	141.26	C20H21FO4	(M+H)+
362.17617	362.17621	0.11	1	193.17	C20H21FO4	(M+NH4)+
363.17984	363.17951	-0.9	1	25.09	C20H21FO4	(M+NH4)+

6 (EB-251)

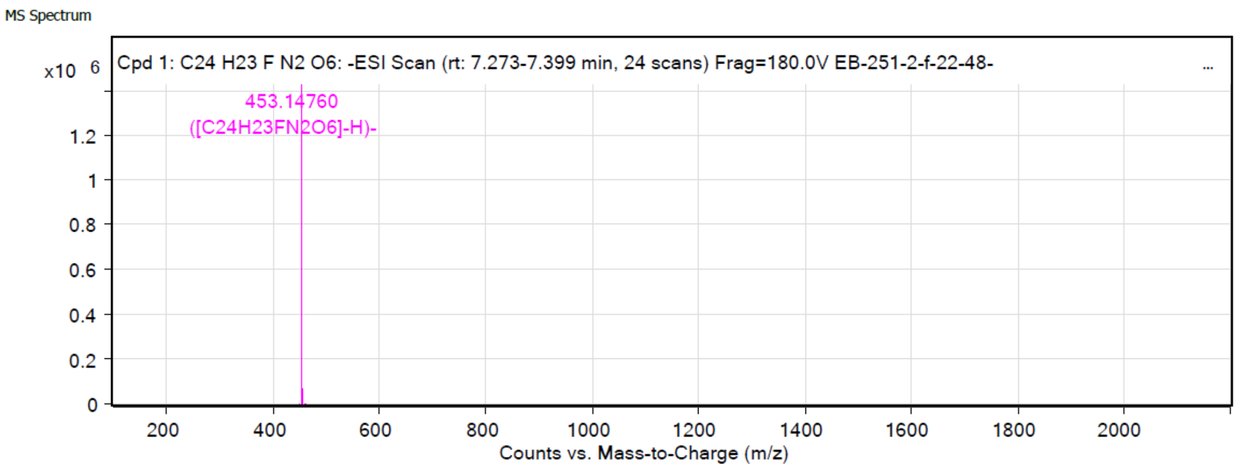

MS Spectrum Peak List

m/z	Calc *m/z*	Diff(ppm)	z	Abund	Formula	Ion
453.1476	453.14674	-1.9	1	1427860.13	C24H23FN2O6	(M-H)-
454.14996	454.14996	-0.01	1	414236.88	C24H23FN2O6	(M-H)-
455.15265	455.15261	-0.1	1	64288.95	C24H23FN2O6	(M-H)-
456.1548	456.15522	0.92	1	7635.9	C24H23FN2O6	(M-H)-
457.15683	457.15778	2.08	1	861.81	C24H23FN2O6	(M-H)-

7

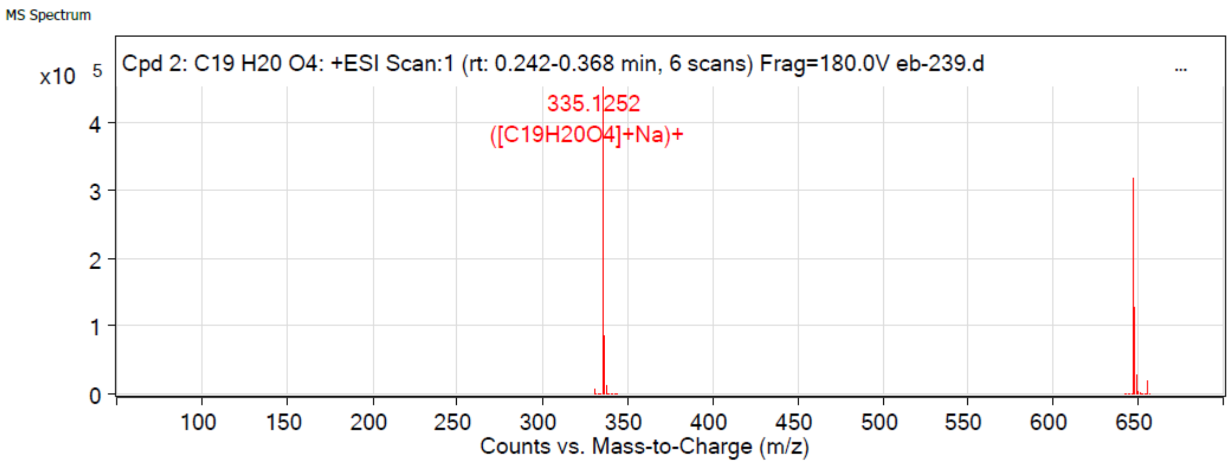

MS Spectrum Peak List

m/z	Calc *m/z*	Diff(ppm)	z	Abund	Formula	Ion
335.1252	335.1254	0.66	1	452433.75	C19H20O4	(M+Na)+
336.1285	336.1288	0.83	1	87885.07	C19H20O4	(M+Na)+
337.1316	337.1314	-0.52	1	11433.36	C19H20O4	(M+Na)+
338.135	338.1341	-2.64	1	982.89	C19H20O4	(M+Na)+
339.1582	339.1367	-63.2	1	867.98	C19H20O4	(M+Na)+
647.2616	647.2615	-0.12	1	317261.56	C19H20O4	(2M+Na)+
648.265	648.2649	-0.08	1	127525.91	C19H20O4	(2M+Na)+
649.2678	649.2679	0.12	1	28849.68	C19H20O4	(2M+Na)+
650.2707	650.2708	0.16	1	4752.04	C19H20O4	(2M+Na)+
651.2749	651.2735	-2.09	1	886.33	C19H20O4	(2M+Na)+

8 (EB-269)

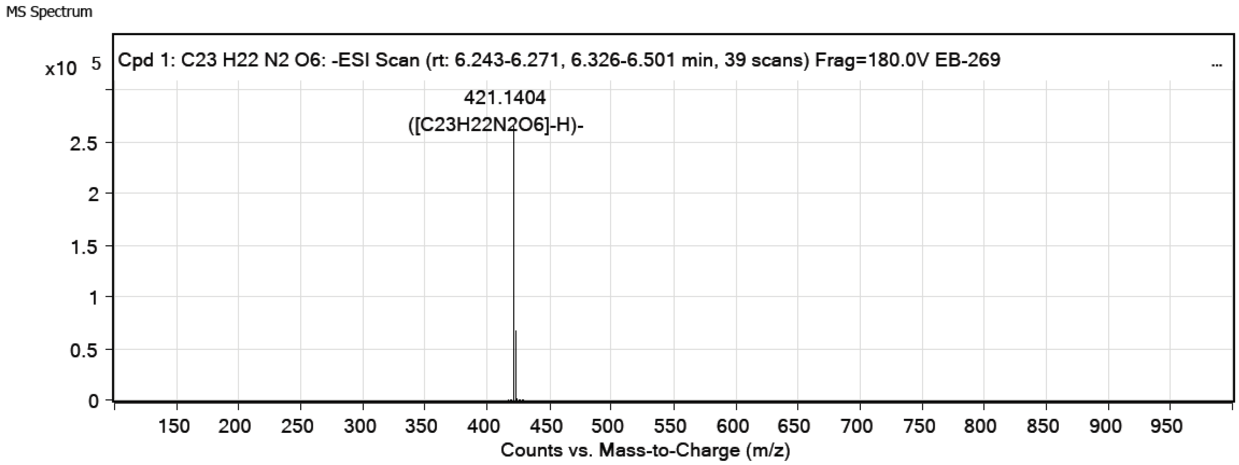

MS Spectrum Peak List

m/z	Calc *m/z*	Diff(ppm)	z	Abund	Formula	Ion
421.1404	421.1405	0.2	1	265944.19	C23H22N2O6	(M-H)-
422.1438	422.1437	-0.08	1	66999.08	C23H22N2O6	(M-H)-
423.1464	423.1463	-0.16	1	10558.53	C23H22N2O6	(M-H)-
424.1488	424.1489	0.28	1	1473.47	C23H22N2O6	(M-H)-
425.1513	425.1515	0.35	1	159.79	C23H22N2O6	(M-H)-

Analytical chemistry: HPLC data (purity)

6 (EB-251)

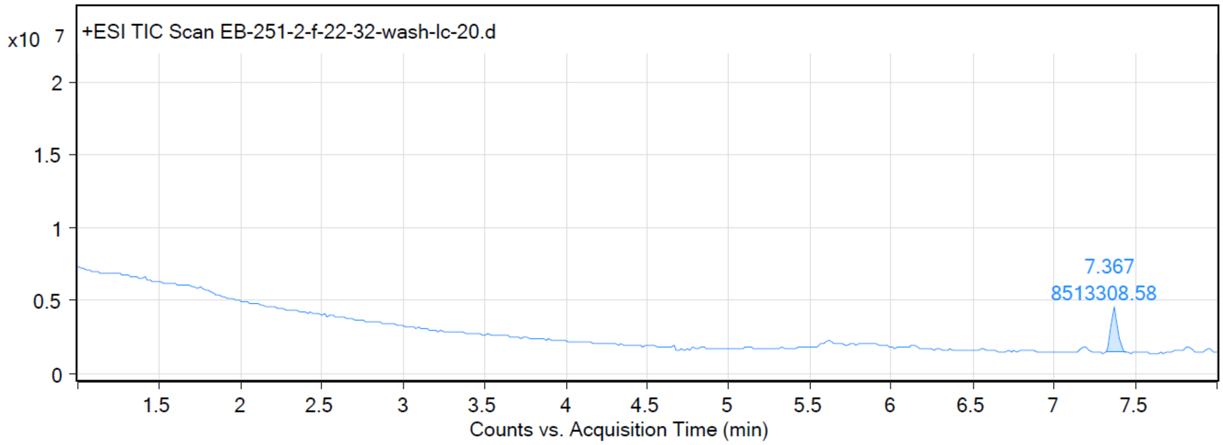

Integration Peak List

Peak	Start	RT	End	Height	Area	Area %
1	7.32	7.367	7.442	3051238.47	8513308.58	100

8 (EB-269)

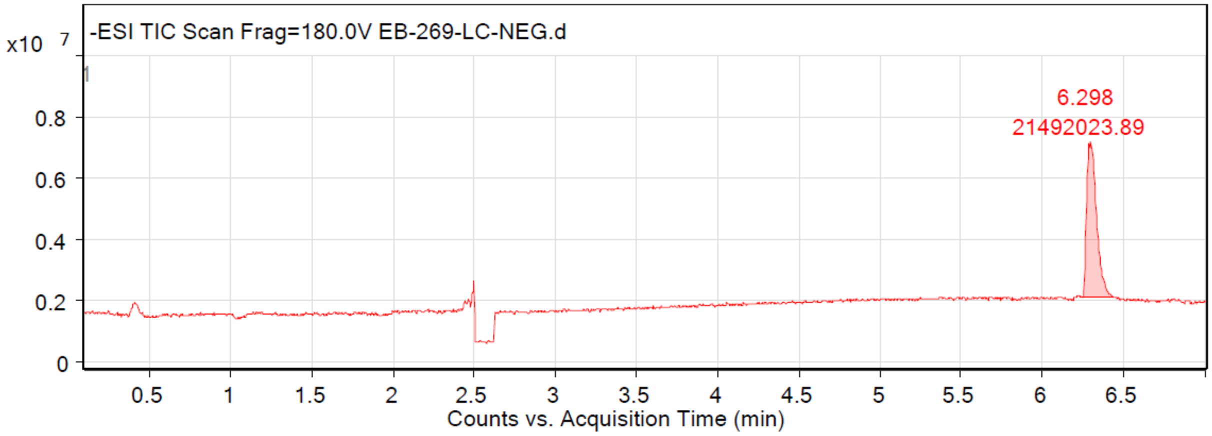

Integration Peak List

Peak	Start	RT	End	Height	Area	Area %
1	6.243	6.298	6.438	5074261.08	21492023.89	100

References:

10. Getter, T., et al., *Novel inhibitors of leukocyte transendothelial migration.* Bioorganic Chemistry, 2019. **92**: p. 103250.
33. Knoevenagel, E., *Condensation von Malonsäure mit aromatischen Aldehyden durch Ammoniak und Amine.* Berichte der deutschen chemischen Gesellschaft, 1898. **31**(3): p. 2596-2619.
34. Node, M., et al., *Hard acid and soft nucleophile systems. 3. Dealkylation of esters with aluminum halide-thiol and aluminum halide-sulfide systems.* The Journal of Organic Chemistry, 1981. **46**(10): p. 1991-1993.
